# Visual Word Segmentation Cues in Tibetan Reading: Comparing Dictionary-Based and Psychological Word Segmentation

**DOI:** 10.3390/jemr18040033

**Published:** 2025-08-04

**Authors:** Dingyi Niu, Zijian Xie, Jiaqi Liu, Chen Wang, Ze Zhang

**Affiliations:** 1 School of Education, Xizang University, Lhasa 850000, China; xiezijian08@163.com (Z.X.); kiki13132024@126.com (J.L.); 15869192822@163.com (C.W.); 2 School of Mathematics, Yunnan Normal University, Kunming 650500, China; zhangze@ynnu.edu.cn

**Keywords:** word segmentation, Tibetan reading, eye movement, psychological word

## Abstract

This study utilized eye-tracking technology to explore the role of visual word segmentation cues in Tibetan reading, with a particular focus on the effects of dictionary-based and psychological word segmentation on reading and lexical recognition. The experiment employed a 2 × 3 design, comparing six conditions: normal sentences, dictionary word segmentation (spaces), psychological word segmentation (spaces), normal sentences (green), dictionary word segmentation (color alternation), and psychological word segmentation (color alternation). The results revealed that word segmentation with spaces (whether dictionary-based or psychological) significantly improved reading efficiency and lexical recognition, whereas color alternation showed no substantial facilitative effect. Psychological and dictionary word segmentation performed similarly across most metrics, though psychological segmentation slightly outperformed in specific indicators (e.g., sentence reading time and number of fixations), and dictionary word segmentation slightly outperformed in other indicators (e.g., average saccade amplitude and number of regressions). The study further suggests that Tibetan reading may involve cognitive processes at different levels, and the basic units of different levels of cognitive processes may not be consistent. These findings hold significant implications for understanding the cognitive processes involved in Tibetan reading and for optimizing the presentation of Tibetan text.

## 1. Introduction

Reading is fundamentally a cognitive activity that involved decoding written symbols into meaningful language, integrating prior knowledge, and constructing meaning through engagement with the text [1]. It is a complex process that encompassed both automatic components, such as recognizing words quickly (fluency), and effortful components, like understanding the deeper meaning of the text (comprehension) [2,3]. Reading plays a critical role in cognitive development, fosters personal growth, and contributes to academic success [4,5,6]. Additionally, reading has profound social and cultural implications [5,7]. Consequently, research on reading holds significant importance.

Word segmentation is an indispensable part of the reading process. Multiple studies have demonstrated that it plays a critical role across various stages of reading comprehension and information processing. Research confirmed that word segmentation, which entailed breaking words in sentences into morpheme units, marks the first step in the reading comprehension process [8]. This indicated word segmentation serves as a foundational cognitive step, assisting readers in parsing vocabulary structures and acquiring semantics, thus laying the groundwork for higher-level understanding. Other findings explicitly noted that word segmentation is essential for information and cognitive processing in reading, acting as a necessary prerequisite for vocabulary recognition and sentence meaning construction [9]. Additionally, the interactive hypothesis proposed by Liu et al. revealed that the word segmentation process influences character recognition through a feedback mechanism, forming a dynamic cyclical processing system [10]. In summary, word segmentation is a fundamental element of reading, vital for both character recognition and advanced comprehension tasks.

In alphabetic writing systems like English, spaces function as inherent word segmentation cues and play a crucial role. Studies show that spaces directly delineate word boundaries, significantly reducing the cognitive load of word recognition [11]. Further research indicated that spaces enable readers to swiftly predict word lengths, optimizing their eye movement strategies. In English reading, approximately 80% of initial fixations fall near the word’s center, known as the “optimal viewing position,” though this strategy failed entirely in texts without spaces [12]. The spelling of English compound words, such as “bell tower” versus “belltower,” influences processing efficiency, with eye movement data revealing that spaced compounds were recognized 150–200 milliseconds faster than their unspaced counterparts due to the additional parsing required for the latter [12]. Evidence also suggested that spaces between words are essential for visual processing and word recognition in languages like English and Spanish that rely on spaces to separate words. Removing spaces could decrease reading speed by 30–50%, as it disrupts eye movement patterns and complicated word boundary identification [13,14,15].

In languages like Chinese, Japanese, and Thai, which lack spaces as word segmentation cues, word segmentation remains highly significant in reading. Research on Chinese readers demonstrated that adding spaces between words enhanced reading speed and reduced fixation times [16,17,18]. Even in typical Chinese sentences without spaces, readers adjusted their fixation positions based on lexical probabilities to segment and recognize words [19,20]. In Japanese, the effect of spaces was less pronounced, yet readers depended heavily on orthographic cues like kanji prominence for segmentation [21]. In Thai, Kohsom and Gobet found that inserting spaces increased reading speed and lowered error rates, with Thai–English bilinguals showing improved performance in spaced Thai texts, as evidenced by shorter fixations and fewer regressions [22].

Tibetan, part of the Tibeto-Burman branch of the Sino-Tibetan language family, employed an alphabetic writing system composed of 30 consonant letters and 4 vowel symbols, augmented by 5 reversed letters (e.g., “ཝ”) [23,24]. Its structure revolved around a “base letter,” with prefixes, superscripts, subscripts, vowel signs, and suffixes added around it [23]. For instance, the Tibetan morpheme “བོད” (meaning “Tibet”) was formed by combining the base consonant “བ” with the vowel “ོ” to create “བོ,” then adding “ད” [9]. Tibetan used the syllable separator “་” (tsheg) to divide syllables—each equivalent to a Chinese character or English morpheme, not a full word—and sentences concluded with the vertical mark “།” (shad), akin to an English period [25]. Tibetan texts lack spaces between words, relying instead on grammatical rules and context for segmentation [25]. These traits suggested that Tibetan, like Chinese and Japanese, lacks inherent word segmentation cues, which could allow researchers to apply similar investigative methods.

Earlier studies proposed that inserting spaces into languages like Chinese and Japanese, which lack word segmentation cues, could facilitate research on word segmentation by providing boundary cues. However, spaces were not the only method employed. Numerous studies utilized color alternation as a segmentation cue, primarily to preserve the text’s original structure and visual fluency without distorting its length. Adding spaces changed sentence length and altered physical layouts [26]. For example, in Vietnamese, where spaces could serve as both syllable and word separators, forcibly adding spaces risked confusing the text’s structure. In contrast, color changes marked word boundaries via blocks or hue differences without modifying character spacing or line width, maintaining the text’s original form [17]. Experiments also indicated that adding extra spaces, particularly between characters in Chinese, disrupted the holistic perception of words, slowing reading speed and disturbing eye movement patterns [17]. Conversely, color-marked boundaries, such as alternating blocks, did not significantly impair reading fluency and aided readers in quickly identifying word units [27]. Thus, both spaces and color alternation served as viable artificial segmentation cues.

Research on languages without word segmentation cues sparked ongoing debate about what constituted a “word.” Some studies argued that words equated to dictionary entries, with Li et al., Noraset et al., and Tissier et al. exploring dictionary use for semantic decomposition, word embedding training, and disambiguation, underscoring its importance in natural language processing [28,29,30]. Historically, Chinese and Tibetan studies segmented words based on dictionary definitions. However, opposing perspectives highlighted dictionary limitations. For example, the sentence “a lot of primary school children are playing all kinds of sports on the playground” contains 15 dictionary words, but lexical combinations such as “a lot of,” “primary school,” and “all kinds of” are readily recognized as single units. When most people perceive these lexical combinations as single units, they can be regarded as psychological words [31,32]. Berko demonstrated through child language acquisition research that children internalized rules rather than merely memorizing dictionary forms, suggesting language processing relied on abstract principles [33]. Yan et al. and Fu compared Chinese lexical (dictionary) words and psychological words, finding the latter easier to process and more likely to serve as the basic unit of reading information processing [31,32].

For Tibetan, comparing dictionary and psychological words was also deemed necessary. Unlike widely used languages like Chinese and English, Tibetan presents a unique challenge: native speakers often exhibited low proficiency and widespread language attrition due to multilingual environments [9]. Tibetan coexisted with dominant languages like Chinese, Hindi, and Nepali. For instance, in the Amdo region (Qinghai, Gansu), the common language shifted from Tibetan to Mandarin, especially among non-Buddhists, where Mandarin became a key tool for social mobility [34]. In Tibetan communities in India and Nepal, English and Hindi dominance in education further constrained Tibetan use [35]. The three major Tibetan dialects (Ü-Tsang, Kham, Amdo) and numerous subdialects show significant variation, with limited mutual intelligibility (e.g., northern vs. southern Kham), often requiring Chinese or other intermediary languages for cross-regional communication. This fragmentation diminished Tibetan’s role as a unified cultural medium [34,35]. Consequently, Tibetan experimental texts need simplification to align with participants’ proficiency levels.

The objective of this experiment is to extend the recent paper by Dingyi Niu. This study and the previous paper [9] both investigated the effect of visual word segmentation cues in Tibetan reading to explore whether these cues facilitate reading and lexical recognition, and to identify the basic information processing unit in Tibetan reading. However, the previous research only examined word segmentation conditions (no segmentation vs. dictionary word segmentation) and segmentation methods (spaces vs. color), whereas the current study further investigates the distinction between dictionary word segmentation and psychological word segmentation. Specifically, this experiment employed a 2 × 3 design with six conditions: (1) normal sentences 1, (2) dictionary word segmentation (spaces), (3) psychological word segmentation (spaces), (4) normal sentences 2, (5) dictionary word segmentation (color alternation), and (6) psychological word segmentation (color alternation). These conditions allowed for a comparison between dictionary-based and psychologically based word segmentation, using both spaces and color alternation as marking methods. Based on previous research that has investigated languages without within-word segmentation cues, this study proposes the following hypotheses, expecting that psychological word segmentation outperforms dictionary word segmentation and that dictionary word segmentation outperforms normal sentences across all relevant metrics.

## 2. Methods and Hypotheses

(1) Inter-word spaces have a facilitative effect on Tibetan reading and aid in lexical recognition. Specifically, the presence of spaces positively influences reading metrics, including average fixation duration, average saccade amplitude, number of fixations, sentence reading time, number of forward saccades, and number of regressions, with psychological word segmentation (spaces) outperforming dictionary word segmentation (spaces), and dictionary word segmentation (spaces) outperforming normal sentences. Similarly, inter-word spaces positively impact lexical recognition metrics, such as first fixation duration, gaze duration, total fixation duration, number of first-pass fixations, total number of fixations, and number of refixations, where psychological word segmentation (spaces) outperforms dictionary word segmentation (spaces), and dictionary word segmentation (spaces) outperforms normal sentences.
(2) Color alternation markings have a facilitative effect on Tibetan reading and aid in lexical recognition. Specifically, the presence of color alternation positively influences reading metrics, including average fixation duration, average saccade amplitude, number of fixations, sentence reading time, number of forward saccades, and number of regressions, with psychological word segmentation (color alternation) outperforming dictionary word segmentation (color alternation), and dictionary word segmentation (color alternation) outperforming normal sentences. Similarly, color alternation positively impacts lexical recognition metrics, such as first fixation duration, gaze duration, total fixation duration, number of first-pass fixations, total number of fixations, and number of refixations, where psychological word segmentation (color alternation) outperforms dictionary word segmentation (color alternation), and dictionary word segmentation (color alternation) outperforms normal sentences.
(3) Psychological words are more likely to be the basic information processing unit in Tibetan reading than dictionary words, and psychological words possess greater psychological reality. In other words, readers demonstrate superior performance in the areas of reading and lexical recognition when exposed to psychological word segmentation conditions (both spaces and color alternation) compared to dictionary word segmentation conditions (both spaces and color alternation), across all relevant metrics.

## 3. Experiment

### 3.1. Participants

To ensure high statistical power, the sample size was calculated using G*Power 3.1.9.7 based on the existing literature [36,37]. The results determined that 44 participants were required for a statistical power of 0.95. The participants were 49 Tibetan university students (24 male, 25 female) aged 18–25 years (M = 21.3, SD = 1.8), recruited from a university in Lhasa, Tibet. All participants were native speakers of Tibetan with normal or corrected-to-normal vision and no reported reading disabilities. They provided informed consent and were compensated with course credit or a small monetary reward.

### 3.2. Design

This experiment utilized a 2 × 3 factorial design, resulting in six experimental conditions. The independent variables were the type of text presentation (two levels) and the segmentation cue (three levels). The six conditions were as follows:

1. Normal Sentence 1 (continuous text with no additional segmentation cues),
2. Dictionary Word Segmentation (spaces inserted between dictionary-defined words),
3. Psychological Word Segmentation (spaces inserted between psychologically salient word units),
4. Normal Sentence 2 (same as Normal Sentence 1, but the color is totally green),
5. Dictionary Word Segmentation with Alternating Colors (dictionary-defined words separated by spaces and presented in alternating colors),
6. Psychological Word Segmentation with Alternating Colors (psychologically salient word units separated by spaces and presented in alternating colors).

**Figure 1:**
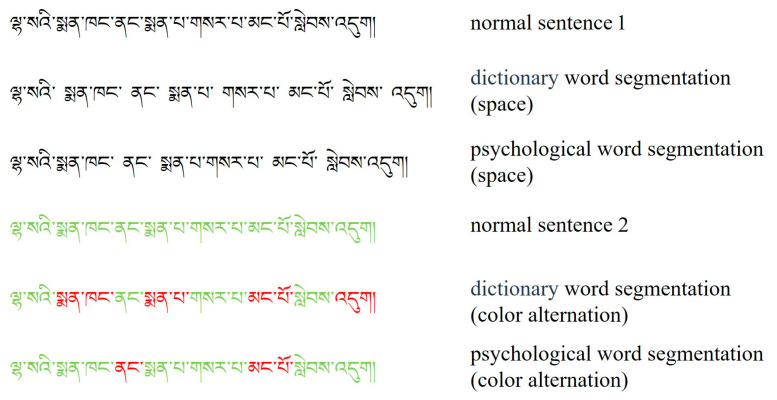
This sentence can be translated to: “Many new doctors have arrived at the Lhasa hospital.” Translation of each word: (1). ལྷ་སའི (lha sa’i): ལྷ་ས (lha sa): Lhasa, the capital city of Tibet. འི (‘i): A genitive marker, meaning “of” or indicating possession. Together: “of Lhasa” or “Lhasa’s.” (2). སྨན་ཁང (sman khang) སྨན (sman): Medicine, medical-related. ཁང (khang): House, building, or place. Together: “hospital” or “medical facility.” (3). ནང (nang) “Inside” or “at.” Indicates location, so here it means “at the hospital.” (4). སྨན་པ (sman pa) Doctor, physician (literally “medicine person”). Refers to medical professionals. (5). གསར་པ (gsar pa) New, fresh. Describes the doctors as “new.” (6). མང་པོ (mang po) Many, a lot. (7). སླེབས (slebs) Arrived, reached. Describes the action of the doctors coming to the hospital. (8). འདུག (‘dug) A verb ending indicating existence or a witnessed event, often implying “are” or “have”.

Here is an example of six experimental conditions in Figure 1.

## 4. Materials

The experimental materials consisted of 102 Tibetan sentences, each approximately 25 words in length (M = 25.1, SD = 1.8). All reading materials were written by 10 native Tibetan speakers, and the writing process referred to our previous reading materials [9]. These sentences were carefully constructed to ensure natural syntax based on a corpus of contemporary Tibetan literature. The method of inserting spaces followed standard procedures established in previous similar studies [16,17,38,39]. Previous studies on the selection of psychological words have mostly employed subject evaluation methods, that is, when more than a certain proportion of subjects consider a multi-word unit as a single word, this word is then recognized as a psychological word [31,32]. In this experiment, the subject evaluation method was also adopted for the selection of psychological words. Each psychological word segmentation of Tibetan sentences has the approval of at least 8 of 10 native Tibetan speakers. To ensure the sentences were sufficiently easy and understandable, six participants who did not participate in the experiment compared the reading difficulty of the materials used this time with those used previously [9] and consistently confirmed that the current materials were easier and more comprehensible. The sentences were divided into six sets, each corresponding to one of the six conditions. For the dictionary word segmentation conditions, word boundaries were determined using a standard Tibetan dictionary. For the psychological word segmentation conditions, boundaries were established based on a pilot study where native speakers identified perceptually salient word units. In the alternating color conditions, adjacent words were displayed in red and green (e.g., word 1 in green, word 2 in red, word 3 in green, etc.) to enhance visual distinction.

### 4.1. Apparatus

The EyeLink1000Plus eye-tracking system produced by SR Research Ltd. (Ottawa, ON, Canada) with a sampling rate of 1000 Hz was used. Data were collected using the default settings for cognitive research (saccade velocity threshold: 30°/s; saccade acceleration threshold: 8000°/s^2^; saccade motion threshold: 0.1°) [9]. The stimuli were presented on an 24.5-inch monitor with a resolution of 1920 × 1080 pixels, at a viewing distance of approximately 65 cm. Based on previous studies [39], the material font was Microsoft Himalaya, size 36 to display the same size, with one sentence displayed on each screen, ensuring readability.

### 4.2. Procedure

Participants were tested individually in a dimly lit, soundproof room. After a brief introduction and calibration of the eye-tracker (using a 9-point calibration grid), participants were instructed to read the sentences silently at their own pace for comprehension. Each trial began with a fixation cross presented at the left of the screen (the position of the sentence’s beginning) for 500 ms, followed by the sentence appearing in one of the six conditions. The order of conditions was counterbalanced across participants using a Latin square design to minimize order effects. After every sentence, participants answered a simple comprehension question (e.g., true/false) to ensure engagement, though these responses were not the primary focus of analysis. The number of correct and incorrect answers was balanced, with 30 true and 30 false responses. The experiment lasted approximately 10 min per participant.

## 5. Experimental Indicators

### 5.1. Global Analysis Indicators

Global analysis indicators provide an overview of eye movement behavior across an entire sentence. All metrics in global analysis reflect reading. Based on prior literature [17,38,40], the following metrics were selected:

**Average Fixation Duration**: This metric represents the mean duration of all fixations within a sentence. A fixation occurs when the eyes pause on a specific point during reading. The average fixation duration is calculated by summing the durations of all fixations in a sentence and dividing by their total number, offering insight into the typical time spent processing each visual stop.

**Average Saccade Amplitude**: This refers to the mean distance traversed by all saccades within a sentence. A saccade is the rapid eye movement between consecutive fixation points. Measured in visual degrees or character spaces, this metric indicates the average spatial extent of eye jumps during reading, reflecting the breadth of visual scanning.

**Number of Fixations**: This is the total count of fixation points recorded within a sentence. A higher number of fixations is often associated with increased cognitive processing load, as it suggests the reader requires more pauses to comprehend the material [41]. This metric serves as an indicator of text difficulty or reader effort.

**Sentence Reading Time**: Defined as the total duration required to read a sentence from its beginning to its end, this metric encompasses all fixations and saccades. It is sensitive to variations in cognitive processing speed, with longer times potentially indicating slower or more effortful comprehension [41].

**Number of Forward Saccades**: This counts the total instances of forward-directed saccades within a sentence, where the eyes move from one fixation point to a subsequent one in the reading direction (e.g., left to right in English). It reflects the progression of reading in a linear fashion.

**Number of Regressions**: This measures the total number of backward saccades, where the eyes revisit earlier portions of the sentence. Regressions may indicate re-processing or clarification efforts, often linked to comprehension difficulties or verification of previously read content.

### 5.2. Local Analysis Indicators

Local analysis indicators focus on eye movements within specific areas of interest (e.g., a word or phrase) within the text, providing detailed insights into the processing of particular elements. Consistent with prior studies [38,42,43], the following metrics were selected, with all the indicators reflecting lexical recognition:

**First Fixation Duration**: This is the duration of the initial fixation on an area of interest. It captures the time spent on the first encounter with a specific region, serving as an early measure of processing effort or lexical access.

**Gaze Duration**: This metric represents the cumulative duration of all fixations on an area of interest during the initial pass, from the first fixation until the eyes exit the area. It reflects the total time devoted to processing that region before moving forward, excluding later revisits.

**Total Fixation Duration**: This encompasses the sum of all fixation durations on an area of interest across the entire reading session, including both the initial pass and any subsequent regressions. It provides a comprehensive measure of attention allocated to the area over time.

**Number of First-Pass Fixations**: This is the count of fixations made on an area of interest during the first continuous reading pass, before the eyes leave the area. It indicates the extent of initial processing required for that region.

**Total Number of Fixations**: This is the overall count of all fixations on an area of interest throughout the reading session, including first-pass fixations and those occurring during regressions. It reflects the cumulative attention directed to the area.

**Number of Refixations**: This metric indicates the number of times an area of interest is fixated upon again after the initial pass. It highlights tendencies to revisit specific regions, potentially due to complexity or ambiguity.

## 6. Result

The average answer accuracy was 94.75%, indicating that the participants care-fully read and understood the sentences. Following the existing studies [9,17,40], data were excluded based on the following four criteria: (1) premature or incorrect key presses that interrupted sentence presentation, (2) invalid data due to loss of tracking, (3) fixation durations of less than 80 ms or greater than 1200 ms, and (4) data points more than three SDs from the mean. Consequently, 2% of the total data in the global analysis and 3.8% of the total data in the local analysis were excluded due to being invalid. Data analysis was conducted in the R programming environment (version 4.4.2).

### 6.1. Global Analysis

Descriptive statistics for each eye movement indicator in various visual segmentation cue conditions are presented in Table 1, and the statistical analysis results are presented in Table 2.

**Figure 2:**
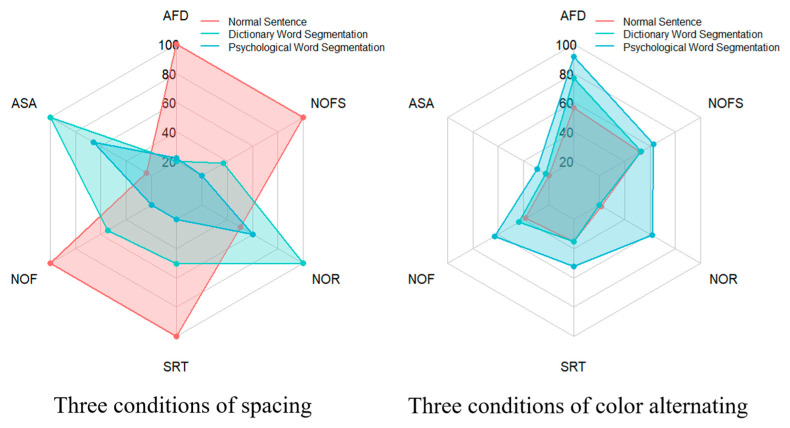
The radar charts of six experimental conditions in global analysis.

To present the statistics of the global analysis indicators more intuitively and clearly, radar charts, as shown in Figure 2, are utilized. The radar charts visualize eye-tracking parameters across six experimental conditions, divided into two charts for clarity. The left chart compares three conditions: normal sentence 1, dictionary-based word segmentation, and psychological word segmentation. The right chart compares normal sentence 2, dictionary-based word segmentation with alternating colors, and psychological word segmentation with alternating colors. Each chart displays six indicators: average fixation duration (AFD), average saccade amplitude (ASA), number of fixations (NOF), sentence reading time (SRT), number of forward saccades (NOFS), number of regressions (NOR). The scales of the left and right radar charts represent the percentage values of the conditions. Specifically, for each metric, the condition with the highest value is set to 100%, and the average values of the other conditions are expressed as percentages relative to this maximum value, as displayed in the radar charts. The scales of both the left and right radar charts are standardized to the same 100% reference. The raw data for each indicator, such as Table S1: 1_Average_duration_of_whole_fixations, can be found in the Supplementary Materials.

Significant main effects of segmentation type were observed across most dependent variables. Both dictionary-based and psycholinguistic segmentation significantly reduced average fixation duration, number of fixations, sentence reading time, and number of forward saccades compared to normal sentences (all *p* < 0.01). Both segmentation types also significantly increased average saccade amplitude (*p* < 0.001). However, only dictionary-based segmentation significantly increased the number of regressions (*p* < 0.01), while psycholinguistic segmentation showed no significant effect on regressions.

The main effect of segmentation method (space vs. color-alternation) was significant for average fixation duration, number of fixations, sentence reading time, and number of forward saccades (all *p* < 0.01), with color-alternation generally producing shorter processing times and fewer eye movements. No significant main effect of segmentation method was found for average saccade amplitude or number of regressions.

Significant interactions between segmentation type and method were found for all dependent variables except number of regressions. Under the color-alternation condition, both dictionary-based and psycholinguistic segmentation showed interaction effects that were contrary to the expected additive effects from the main effects alone (all *p* < 0.05), suggesting that the benefits of segmentation were attenuated when combined with color cues.

Simple main effects analysis revealed that segmentation benefits were primarily driven by the space condition. In the space condition, both segmentation types significantly improved all eye movement measures compared to normal sentences (*p* < 0.01) except the number of regressions. However, under the color-alternation condition, these simple main effects were generally not significant.

Custom contrast analyses indicated that psycholinguistic segmentation was significantly more effective than dictionary-based segmentation in reducing average saccade amplitude in the space condition (*p* < 0.001). Additionally, psycholinguistic segmentation showed marginally superior effects compared to dictionary-based segmentation on number of fixations and sentence reading time in the space condition (both *p* < 0.1), suggesting a trend toward greater effectiveness of the psycholinguistic approach for these measures. Furthermore, psycholinguistic segmentation showed significantly fewer regressions than dictionary-based segmentation in the space condition (*p* < 0.05), while the opposite pattern emerged in the color-alternation condition (*p* < 0.05). For other eye movement measures, the two segmentation types showed comparable effects in both the space and color-alternation conditions.

### 6.2. Local Analysis

Descriptive statistics for each eye movement indicator in various visual segmentation cue conditions are presented in Table 3, and the statistical analysis results are presented in Table 4.

**Figure 3:**
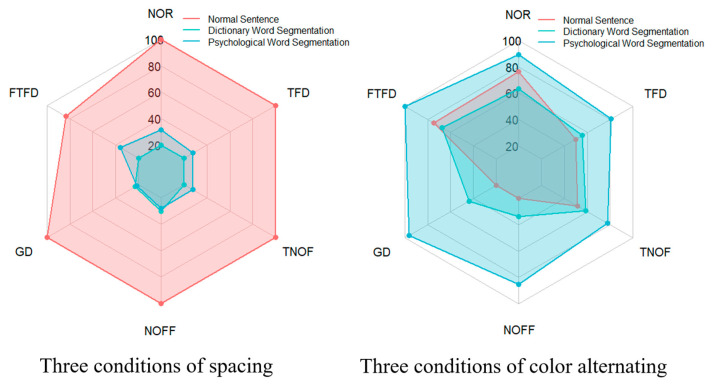
The radar charts of six experimental conditions in local analysis.

To present the statistics of the local analysis indicators more intuitively and clearly, radar charts, as shown in Figure 3, are utilized. The radar charts visualize eye-tracking parameters across six experimental conditions, divided into two charts for clarity. The left chart compares three conditions: normal sentence 1, dictionary-based word segmentation, and psychological word segmentation. The right chart compares normal sentence 2, dictionary-based word segmentation with alternating colors, and psychological word segmentation with alternating colors. Each chart displays six indicators: number of refixations (NOR), first fixation duration (FFD), gaze duration (GD), number of first-pass fixations (NOFF), total number of fixations (TNOF), total fixation duration (TFD). The scales of the left and right radar charts represent the percentage values of the conditions. Specifically, for each metric, the condition with the highest value is set to 100%, and the average values of the other conditions are expressed as percentages relative to this maximum value, as displayed in the radar charts. The scales of both the left and right radar charts are standardized to the same 100% reference.

Significant main effects of segmentation type were observed for several eye movement measures. Both dictionary-based and psycholinguistic segmentation significantly reduced number of refixations (*p* < 0.05), number of first-pass fixations (*p* < 0.05), total number of fixations (*p* < 0.01), and total fixation duration (*p* < 0.01) compared to normal sentences. Both segmentation types showed marginally significant effects on gaze duration (*p* ≈ 0.06), while no significant main effects were found for first fixation duration.

The main effect of segmentation method (space vs. color-alternation) was significant for number of first-pass fixations (*p* < 0.05), total number of fixations (*p* < 0.05), and total fixation duration (*p* < 0.05), with color-alternation generally reducing these measures. Segmentation method showed a marginally significant effect on gaze duration (*p* ≈ 0.05) but no significant effects on number of refixations or first fixation duration.

Significant interactions between segmentation type and method were found for several measures. Psycholinguistic segmentation showed significant interactions for gaze duration (*p* < 0.05), number of first-pass fixations (*p* < 0.01), total number of fixations (*p* < 0.01), and total fixation duration (*p* < 0.01). Dictionary-based segmentation showed significant interactions for total number of fixations (*p* < 0.01) and total fixation duration (*p* < 0.05). These interactions generally indicated that the benefits of segmentation were attenuated when combined with color cues. No significant interactions were observed for number of refixations or first fixation duration.

Simple main effects analysis revealed that segmentation benefits were primarily evident in the space condition. In the space condition, both segmentation types significantly reduced number of first-pass fixations (*p* < 0.05), total number of fixations (*p* < 0.01), and total fixation duration (*p* < 0.05). For number of refixations, dictionary-based segmentation showed significant reduction (*p* < 0.05) while psycholinguistic segmentation showed marginal significance (*p* = 0.054) in the space condition. Both segmentation types showed marginally significant effects on gaze duration in the space condition. Under the color-alternation condition, simple main effects were generally not significant across all measures.

Custom contrast analyses revealed no significant differences between dictionary-based and psycholinguistic segmentation for any of the eye movement measures in either the space or color-alternation conditions (all *p* > 0.05), suggesting that both segmentation types had comparable effects on these measures.

## 7. General Discussion

This study adopted a 2 × 3 experimental design with six conditions: normal sentence 1, dictionary word segmentation (spacing), psychological word segmentation (spacing), normal sentence 2, dictionary word segmentation (color alternation), and psychological word segmentation (color alternation). The aim was to investigate the effects of different visual word segmentation cues (spacing vs. color alternation) and segmentation methods (dictionary words vs. psychological words) on reading processing. By analyzing eye movement data, we identified interaction effects, simple effects, and results from custom contrast analyses. These factors are discussed in detail below.

### 7.1. Interaction Effect and Simple Effect

Under these 7 indicators, the interaction effect between the two factors (segmentation type and segmentation method) is significant: average fixation duration, average saccade amplitude, number of fixations, sentence reading time, number of forward saccades, total number of fixations, total fixation duration. And in further simple effect analysis, the following 10 indicators all show significance under the spacing condition: average fixation duration, average saccade amplitude, number of fixations, sentence reading time, number of regressions, number of forward saccades, number of refixations, number of first-pass fixations, total number of fixations, total fixation duration. Additionally, one indicator shows marginal significance: gaze duration. However, under the color alternation condition, except number of regressions, no indicator shows significance.

Combining the simple effect analysis, we could infer that: under the spacing condition, whether dictionary words or psychological words, there are basically significant differences compared to the baseline condition (normal sentence condition). However, under the color alternation condition, whether dictionary words or psychological words, there are basically no significant differences compared to the baseline condition (normal sentence condition). This means that while spacing condition has substantial facilitating effect on the text in this experiment, color alternation has no substantial facilitating effect on the text in this experiment.

### 7.2. Custom Contrast Analysis

For custom contrast analysis results, the following 8 indicators show no significant difference between dictionary word segmentation and psychological word segmentation: average fixation duration, number of forward saccades, number of refixations, first fixation duration, gaze duration, number of first-pass fixations, total number of fixations, and total fixation duration. The following 2 indicators show that under the spacing condition, there is a marginally significant difference between psychological word segmentation and dictionary word segmentation, with psychological word segmentation performing better than dictionary word segmentation: sentence reading time, number of fixations. The following 2 indicators show that under the spacing condition, there is a significant difference between psychological word segmentation and dictionary word segmentation, with psychological word segmentation performing better than dictionary word segmentation: average saccade amplitude, number of regressions. This suggests that dictionary word segmentation and psychological word segmentation are similar in most cases. However, based on the indicators where each excels, we can infer that: psychological and dictionary word segmentation may better facilitate different levels of cognitive processes in reading.

### 7.3. The Effect of Inter-Word Spaces in Tibetan Reading

According to the hypothesis proposed by Bai et al., there is a trade-off between the interference effect caused by readers’ familiarity with the text presentation and the facilitating effect of visual word segmentation cues [17]. This means that the less familiar a person is with a language, the greater the facilitating effect of spaces as word segmentation cues on their reading of that language. Since most Tibetans may have experienced native language attrition, the facilitating role of inter-word spacing in reading is understandable for the majority of them. This is also consistent with our proposed hypothesis: whether it is inter-word spacing for dictionary words or psychological words, compared to normal sentence conditions, both reading performance and lexical recognition performance of the participants show significant improvement. However, psychological words do not outperform dictionary words on most metrics, possibly because the reduced difficulty of the reading material amplifies the differences among Tibetan participants. In reading, there are two approaches: “subvocal reading” and “direct visual-to-mind processing.” For “subvocal reading,” participants need to first mentally form the pronunciation of the word being read before comprehending its meaning [44,45,46]. For “direct visual-to-mind processing,” participants can directly understand the meaning of the reading material through visual text [45,47,48]. When the reading material is simple enough, some participants may be able to complete all reading tasks using “direct visual-to-mind processing,” while others still rely on “subvocal reading” [46].

This leads to increased variability among participants. This aligns with the records from our experiment: among our 49 participants, 11 completed all reading tasks within 5 min, with the fastest taking only 2 min and 49 s. In contrast, 11 participants took over 9 min, with the slowest requiring 13 min and 42 s. The average reading time per sentence was 2819 milliseconds, with a standard deviation of 1807 milliseconds—a level of dispersion much greater than in previous studies (e.g., in Wang et al.’s experiment, the average reading times per sentence for normal sentences and sentences with inter-word spacing were 1402 and 1156 milliseconds, respectively) [9].

### 7.4. The Effect of Color Alternation Markings in Tibetan Reading

Under the condition of color alternation, the vast majority of indicators did not show significant differences. Possible reasons, apart from the excessive inter-subject variability caused by overly simple reading materials mentioned in the previous section, could also include the possibility that the facilitative effect produced by color alternation itself is not as strong as the facilitative effect produced by spaces. This might be because the word boundaries marked by color alternation are not as prominent as those marked by spaces. In the experiment conducted by Wang et al., word segmentation with spaces, compared to normal sentences, showed significance in 11 out of 13 indicators, with each significant result reaching a *p* < 0.001 level. However, word segmentation with color alternation, compared to normal sentences, showed significance in only 9 out of 13 indicators, with 7 indicators significant at the *p* < 0.05 level and only 2 indicators reaching the *p* < 0.01 level. No indicators reached the *p* < 0.001 level of significance [9]. This suggests that the facilitative effect of color alternation is not as substantial as that of spacing. This further explanation might be that color alternation might introduce additional cognitive load rather than facilitating processing [49]. Readers may need to consciously interpret the alternating colors as word boundaries, whereas spaces are a more automatic and universally recognized cue. This conscious interpretation process may lead to weaker cognitive effects on the measured indicators.

### 7.5. The Basic Information Processing Units in Tibetan Reading

The word-spacing condition, encompassing both psychological word segmentation and dictionary word segmentation, exhibited fewer fixations, fewer forward saccades, fewer regressions, fewer first-pass fixations, a lower total number of fixations, a shorter total fixation duration, a shorter gaze duration, fewer refixations, and shorter sentence reading times, alongside a slightly larger average saccade amplitude and a shorter average fixation duration, compared to the normal sentence condition. While the psychological and dictionary word segmentation approaches showed almost no significant differences between each other, they both demonstrated a clear advantage over the baseline (normal sentence) across these indicators. This suggests that explicit word segmentation, regardless of whether it is psychologically or dictionary-driven, enhances reading efficiency relative to unsegmented text. Thus, it seems that both psychological words and dictionary words may be the basic units of Tibetan reading.

Psychological word segmentation appears to reduce “sentence reading time” and “number of fixations” because it matches how readers naturally think about words. When people read, they instinctively group letters and syllables into meaningful units based on their language experience and intuition [50,51]. In Tibetan, readers use visual markers called tshegs as guides, but psychological segmentation goes further—it reflects how readers naturally break down text in their minds. This natural approach helps readers process information more smoothly. Because the word boundaries feel intuitive and meaningful to them, readers need fewer stops (fixations) to understand the text, which speeds up their overall reading. For instance, psychological word boundaries often match what readers expect based on their experience with the language, reducing the mental effort needed to decode sentences.

On the other hand, dictionary word segmentation might outperform in “average saccade amplitude” and “number of regressions” because it adheres to standardized, linguistically defined word boundaries, providing a more consistent structure. When text follows dictionary rules, readers can make longer eye movements (larger “average saccade amplitude”) because they trust that each dictionary-defined word forms a complete, uninterrupted unit. They do not worry about breaking up words in the middle, so they can jump further across the text with confidence [52,53]. This consistency could also reduce backward eye movements (number of regressions), as readers are less likely to backtrack to reprocess ambiguous or unfamiliar segments [54,55]. Dictionary segmentation, being rule-based and aligned with formal linguistic norms, might prevent the uncertainty that could arise from more subjective psychological boundaries, leading to smoother forward progression through the text.

This indicates that reading may involve different levels of cognitive processes, and the basic units of cognitive processes at different levels may be inconsistent. Therefore, the basic units of Tibetan reading may simultaneously include psychological words and dictionary words.

## 8. Limitations and Prospects

Considering the significant individual differences observed among Tibetan participants when reading low-difficulty Tibetan sentences, in our subsequent research, we will establish both the difficulty level of Tibetan reading texts and participants’ Tibetan reading proficiency as variables. This approach will allow us to study individual differences as an independent variable and more clearly observe the interaction effects between text difficulty and participants’ Tibetan language proficiency.

Additionally, our experiment revealed that Tibetan is not a uniform language. There were instances where Tibetan sentences written by some Tibetan individuals were incomprehensible to others. Although we attempted to select sentences that were widely accepted among Tibetan people for our experiment, the internal variations within the Tibetan language informed us of the necessity to use standardized sentences. Therefore, in future studies, we will strive to use Tibetan sentences from middle school examinations, college entrance examinations, and officially published materials as our experimental materials.

Finally, considering that Tibetan text naturally lacks spaces as visual word segmentation cues yet reading proceeds normally, it is essential to explore potential advanced linguistic word segmentation cues in Tibetan (such as lexical positional probability information) [56,57,58].

## 9. Conclusions

For simple Tibetan sentences, inter-word spacing (whether dictionary words or psychological words) can facilitate Tibetan reading and Tibetan vocabulary recognition. Color alternation has no effect on Tibetan reading or Tibetan vocabulary recognition. Tibetan reading may involve cognitive processes at different levels, and the basic units of different levels of cognitive processes may not be consistent. Dictionary words and psychological words may have greater facilitating effects on different levels of cognitive processes in Tibetan reading. Dictionary words and psychological words may jointly constitute the basic units of Tibetan reading.

## Figures and Tables

**Table 1:** Descriptive statistics of the global analysis indicators (standard deviations in parentheses).

	Average Fixation Duration	Average Saccade Amplitude	Number of Fixations	Sentence Reading Time	Number of Regressions	Number of Forward Saccades
Normal Sentence 1	180.051 (44.901)	4.165 (4.881)	10.732 (6.321)	3056.004 (1888.369)	3.584 (3.051)	8.233 (4.916)
Dictionary Word Segmentation (spaces)	172.190 (37.764)	5.204 (5.646)	10.089 (5.926)	2826.196 (1760.988)	3.967 (3.302)	7.361 (4.602)
Psychological Word Segmentation (spaces)	172.393 (42.858)	4.738 (5.150)	9.595 (6.055)	2685.635 (1804.014)	3.659 (3.376)	7.126 (4.815)
Normal Sentence 2	175.803 (42.718)	4.108 (4.634)	9.857 (6.218)	2756.544 (1801.468)	3.359 (3.338)	7.577 (4.819)
Dictionary Word Segmentation (color alternation)	177.778 (42.385)	4.143 (4.699)	9.929 (5.943)	2754.793 (1760.599)	3.347 (3.008)	7.584 (4.754)
Psychological Word Segmentation (color alternation)	179.220 (44.215)	4.232 (4.809)	10.205 (6.437)	2833.881 (1804.929)	3.670 (3.440)	7.719 (4.895)

**Table 2:** T-values (and significance) for main effects, interactions, simple effects, and custom contrasts in global analysis. The simple effect of color alternation was nonsignificant and thus omitted.

	Average Fixation Duration	Average Saccade Amplitude	Number of Fixations	Sentence Reading Time	Number of Regressions	Number of Forward Saccades
Main Effects: Dictionary-based vs. Normal	−5.845 ***	13.404 ***	−2.899 **	−2.921 **	2.603 **	−4.529 ***
Main Effects: Psycholinguistic vs. Normal	−5.248 ***	7.483 ***	−4.635 ***	−4.708 ***	0.511	−5.756 ***
Main Effects: Segmentation Method	−2.750 **	−0.555	−3.494 ***	−3.823 ***	−1.525	−3.417 ***
Interaction Effect: Dictionary-based (Color)	4.885 ***	−9.106 ***	1.996 *	1.996 *	−1.885 §	3.244 **
Interaction Effect: Psycholinguistic (Color)	4.903 ***	−4.238 ***	4.033 ***	3.988 ***	1.128	4.591 ***
Simple Main Effects: Dictionary-based (Space)	−5.930 ***	12.787 ***	−2.840 **	−2.817 **	2.643**	−4.546 ***
Simple Main Effects: Psycholinguistic (Space)	−5.344 ***	6.992 ***	−4.582 ***	−4.559 ***	0.521	−5.773 ***
Custom Contrast: Space Condition	0.598	−5.684 ***	−1.724 §	−1.784 §	−2.091 *	−1.23
Custom Contrast: Color Condition	0.618	1.126	1.148	1.034	2.171 *	0.676

* *p* < 0.05; ** *p* < 0.01; *** *p* < 0.001; § *p* < 0.1.

**Table 3:** Descriptive statistics of the local analysis indicators (standard deviations in parentheses).

	Number of Refixations	First Fixation Duration	Gaze Duration	Number of First-Pass Fixations	Total Number of Fixations	Total Fixation Duration
Normal Sentence 1	1.231 (1.336)	183.115 (101.347)	704.577 (752.028)	3.654 (3.237)	6.538 (4.062)	1368.154 (1113.218)
Dictionary Word Segmentation (spaces)	0.455 (0.800)	168.136 (96.826)	358.500 (262.896)	2.136 (1.167)	2.818 (2.039)	463.500 (389.658)
Psychological Word Segmentation (spaces)	0.565 (0.844)	171.913 (65.739)	351.043 (323.837)	2.087 (1.649)	3.174 (2.367)	552.130 (473.019)
Normal Sentence 2	1.000 (1.024)	181.045 (88.283)	343.955 (427.593)	1.909 (1.743)	4.273 (3.453)	798.500 (733.170)
Dictionary Word Segmentation (color alternation)	0.875 (0.797)	179.375 (111.714)	451.500 (571.092)	2.208 (2.085)	4.625 (3.019)	865.833 (644.746)
Psychological Word Segmentation (color alternation)	1.125 (1.154)	187.000 (99.135)	687.083 (559.872)	3.333 (2.496)	5.500 (3.230)	1153.833 (761.722)

**Table 4:** T-values (and significance) for main effects, interactions, simple effects, and custom contrasts in local analysis. The simple effect of color alternation was nonsignificant and thus omitted.

	Number of Refixations	First Fixation Duration	Gaze Duration	Number of First-Pass Fixations	Total Number of Fixations	Total Fixation Duration
Main Effects—Word Segmentation Type (Dictionary)	−2.584 *	−0.454	−1.845 §	−2.173 *	−3.746 ***	−3.273 **
Main Effects—Word Segmentation Type (Psycholinguistic)	−2.240 *	−0.373	−1.895 §	−2.253 *	−3.408 **	−2.978 **
Main Effects—Segmentation Method	−0.77	−0.116	−1.964 §	−2.517 *	−2.316 *	−2.136 *
Interaction Effect—Dictionary	1.521	0.32	1.726 §	1.836 §	2.902 **	2.522 *
Interaction Effect—Psycholinguistic	1.856 §	0.423	2.649 *	3.023 **	3.276 **	3.047 **
Simple Main Effects—Space (Dictionary)	−2.361 *	−0.566	−1.95 §	−2.144 *	−3.412 **	−2.827 **
Simple Main Effects—Space (Psycholinguistic)	−2.035 §	−0.432	−2.004 §	−2.226 *	−3.079 **	−2.568 *
Custom Contrast—Space	0.359	0.082	−0.036	−0.057	0.36	0.304
Custom Contrast—Color-Alternation	0.835	0.229	1.259	1.614	0.88	1.039

* *p* < 0.05; ** *p* < 0.01; *** *p* < 0.001; § *p* < 0.1.

## Data Availability

The original contributions presented in the study are included in the Supplementary Materials, further inquiries can be directed to the corresponding author.

## Supplementary Materials

The following supporting information can be downloaded at: https://www.mdpi.com/article/10.3390/jemr18040033/s1, Table S1: 1_Average_duration_of_whole_fixations.
