# Supplementary figures and images for: Visual Word Segmentation Cues in Tibetan Reading: Comparing Dictionary-Based and Psychological Word Segmentation
Source: J Eye Mov Res. 2025 Aug 4;18(4):33. doi: 10.3390/jemr18040033 (PMC12387474; doi:10.3390/jemr18040033)

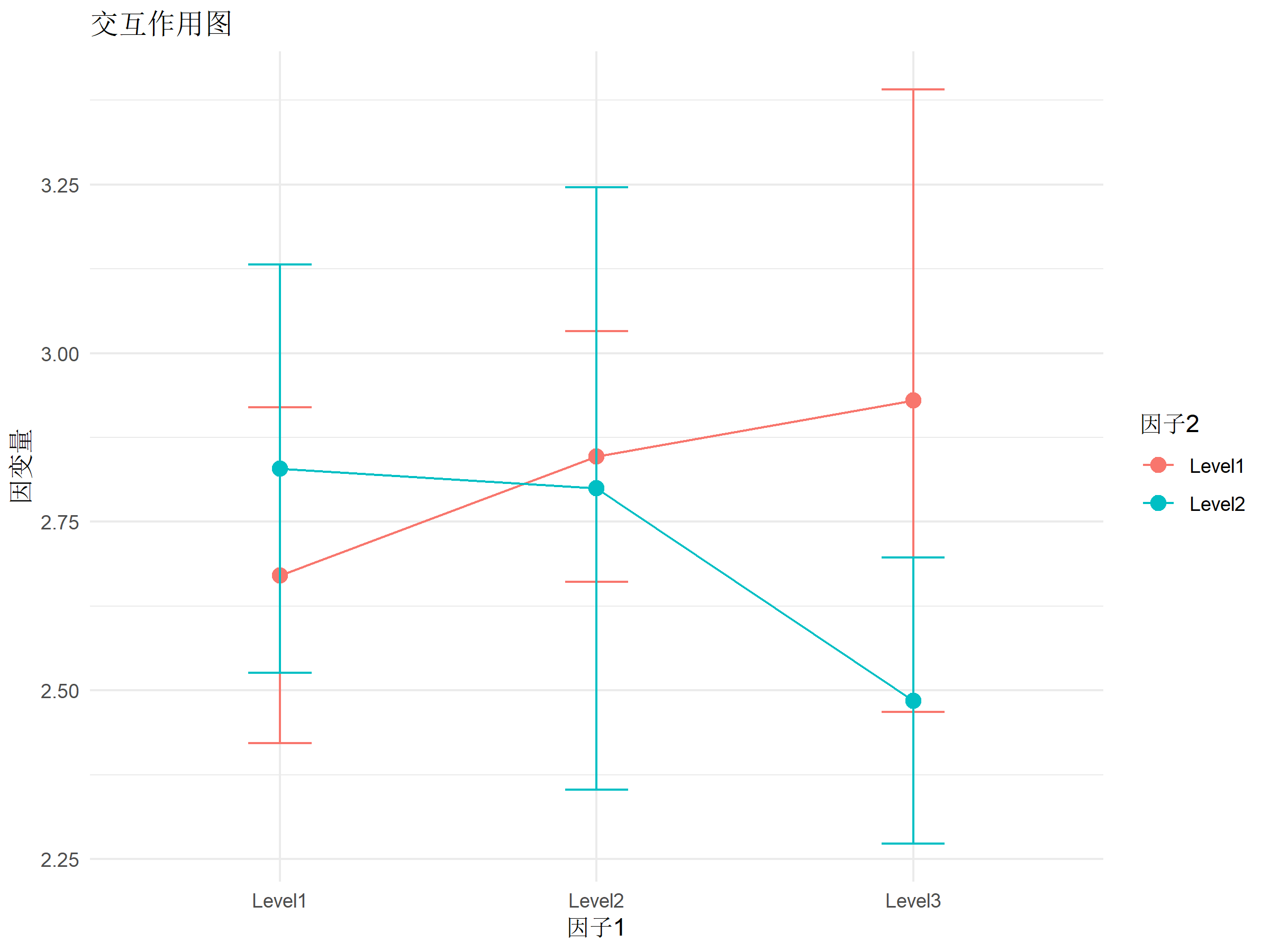

Supplement: Supplementary file 1 [file jemr-18-00033-s001.zip › local/2_fixation_position/interaction_plot.png]

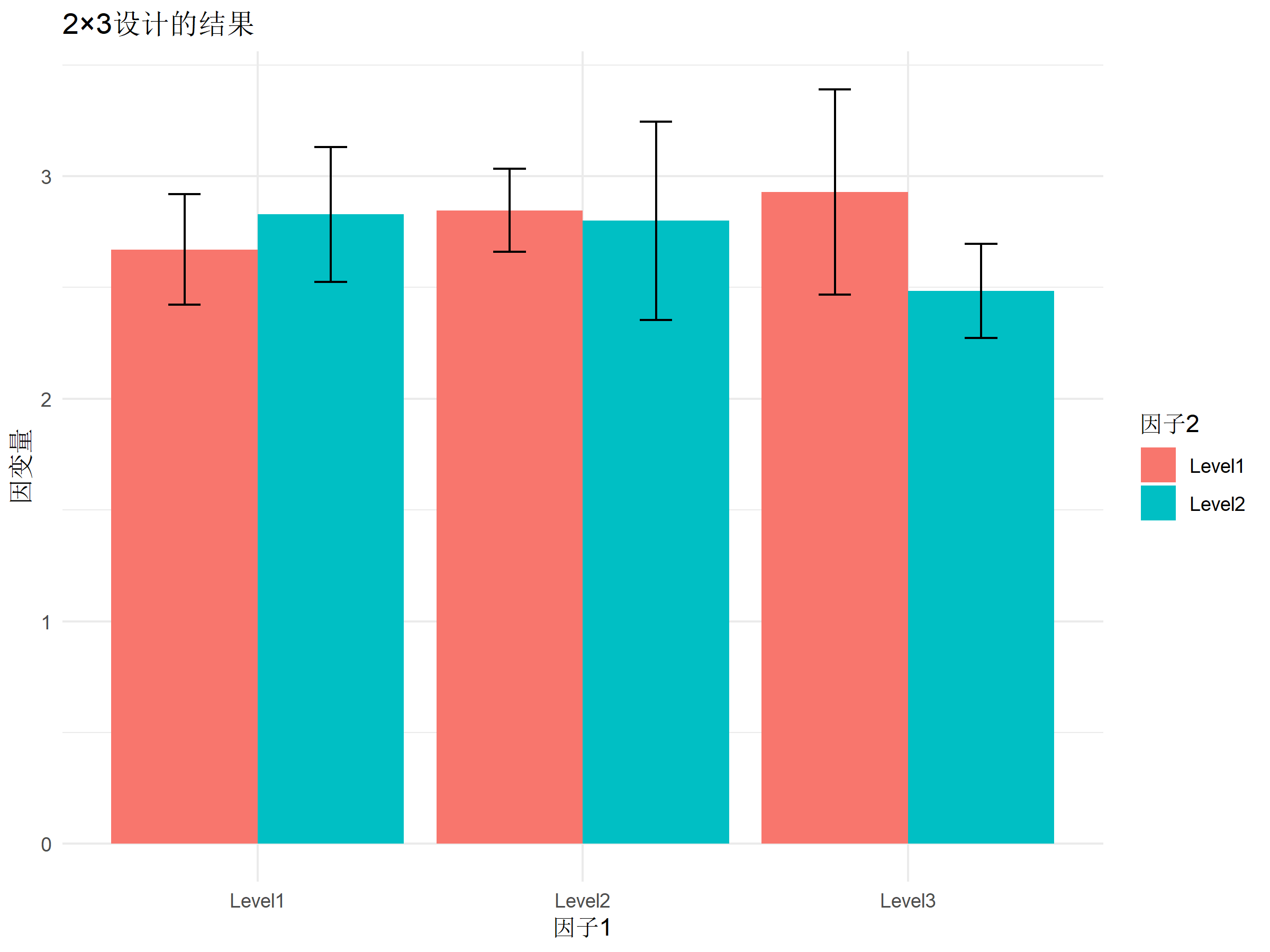

Supplement: Supplementary file 1 [file jemr-18-00033-s001.zip › local/2_fixation_position/result_plot.png]

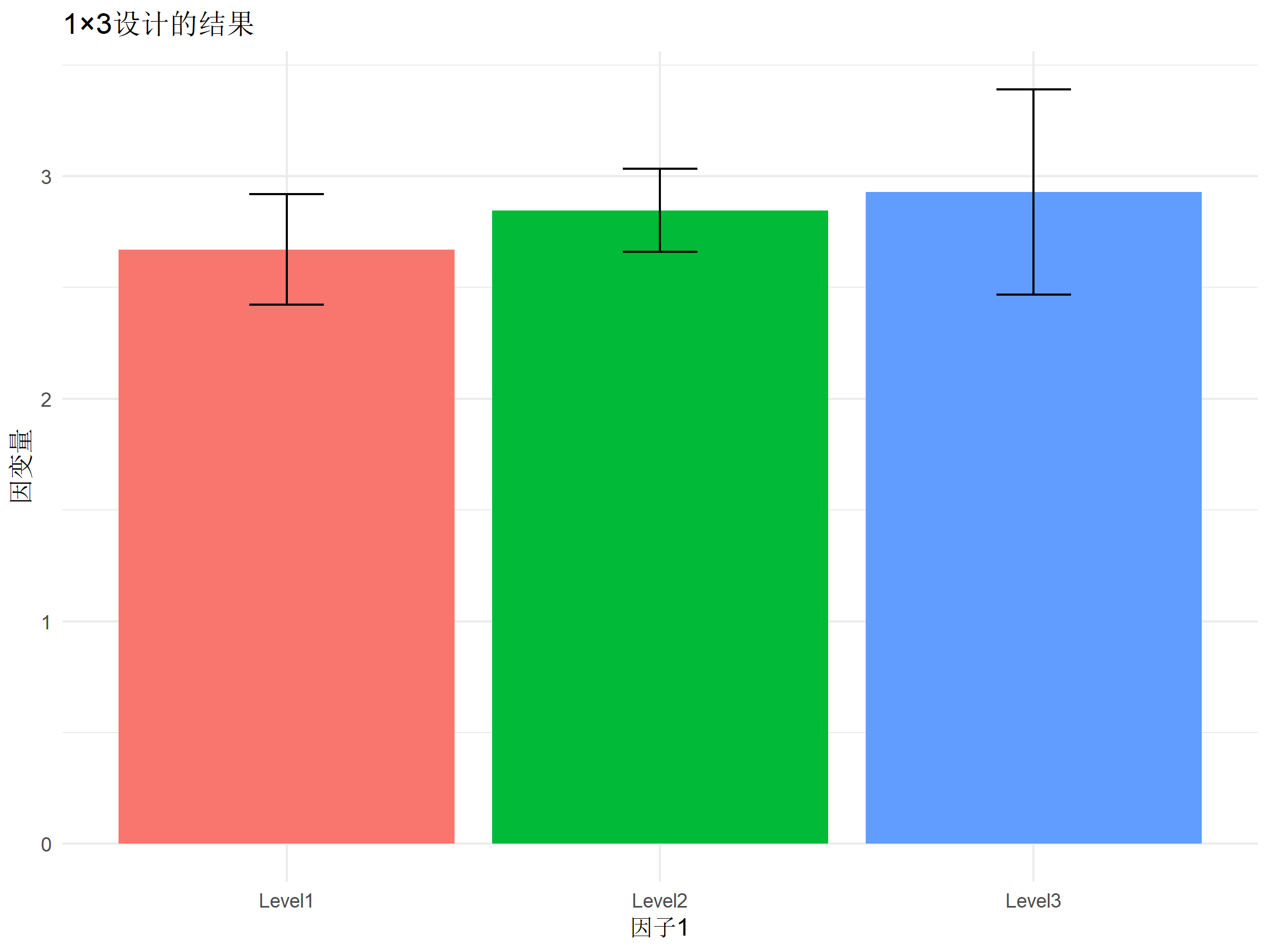

Supplement: Supplementary file 1 [file jemr-18-00033-s001.zip › local/2_fixation_position2/result_plot.png]

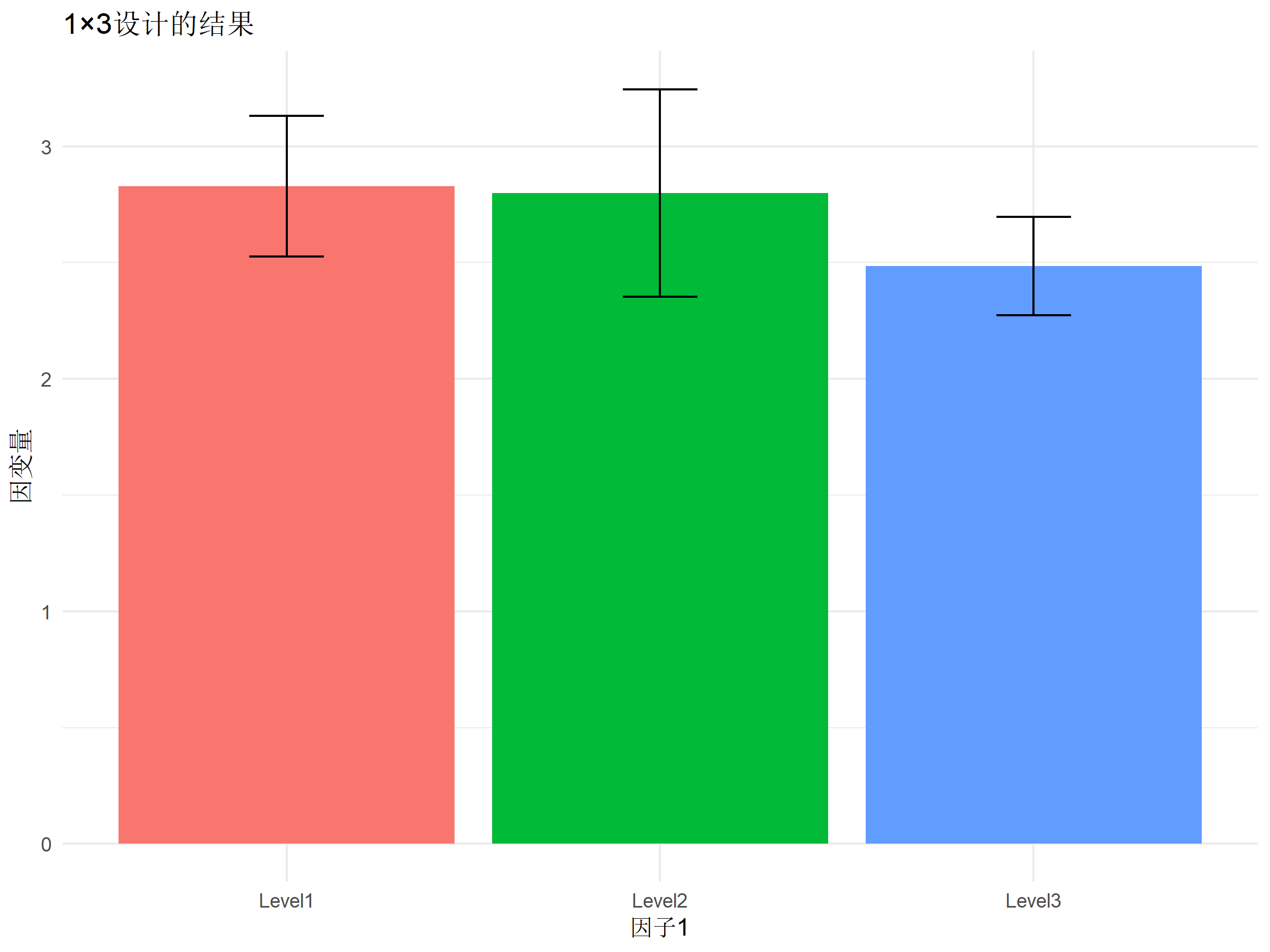

Supplement: Supplementary file 1 [file jemr-18-00033-s001.zip › local/2_fixation_position3/result_plot.png]

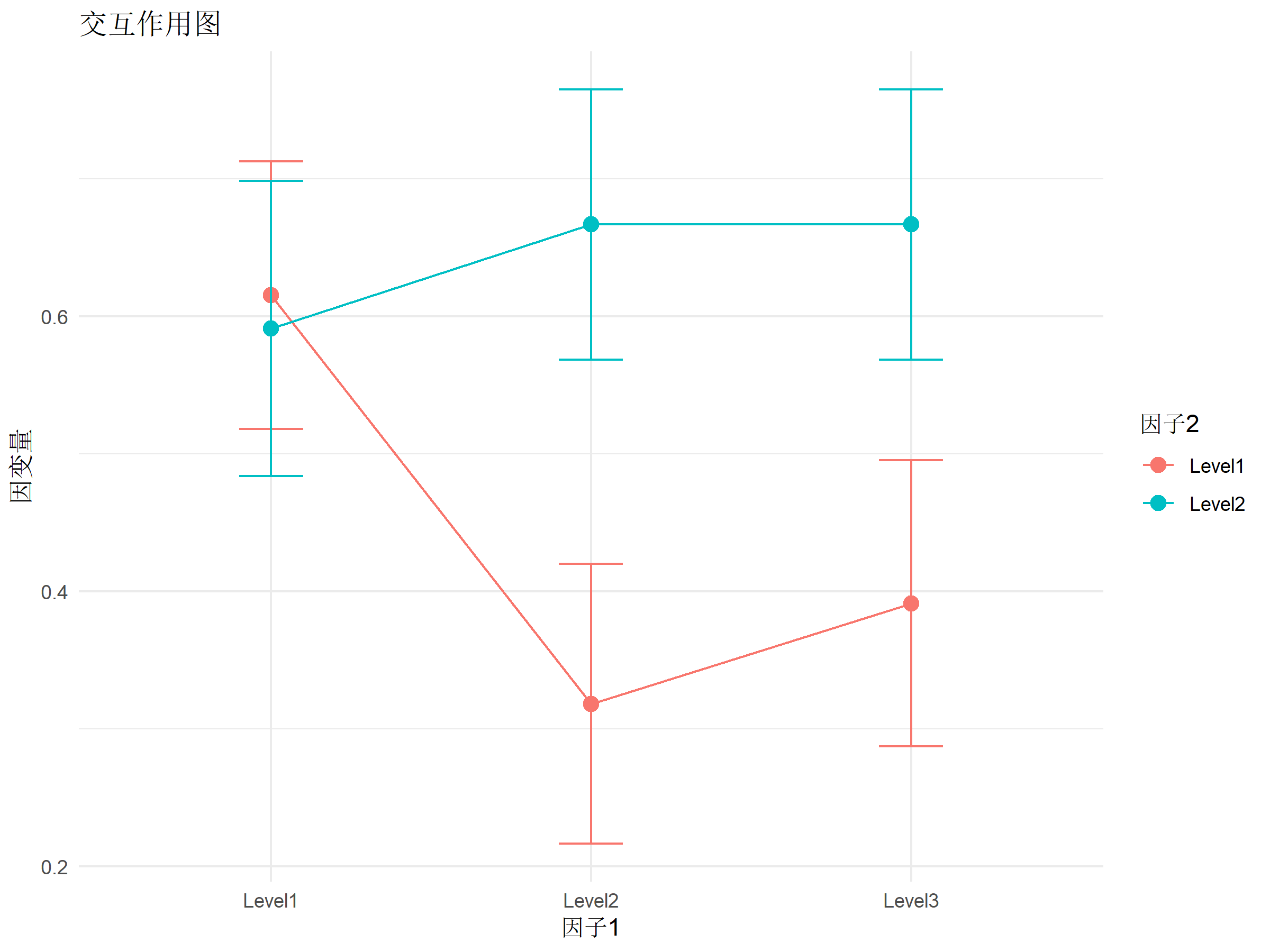

Supplement: Supplementary file 1 [file jemr-18-00033-s001.zip › local/2_probability/interaction_plot.png]

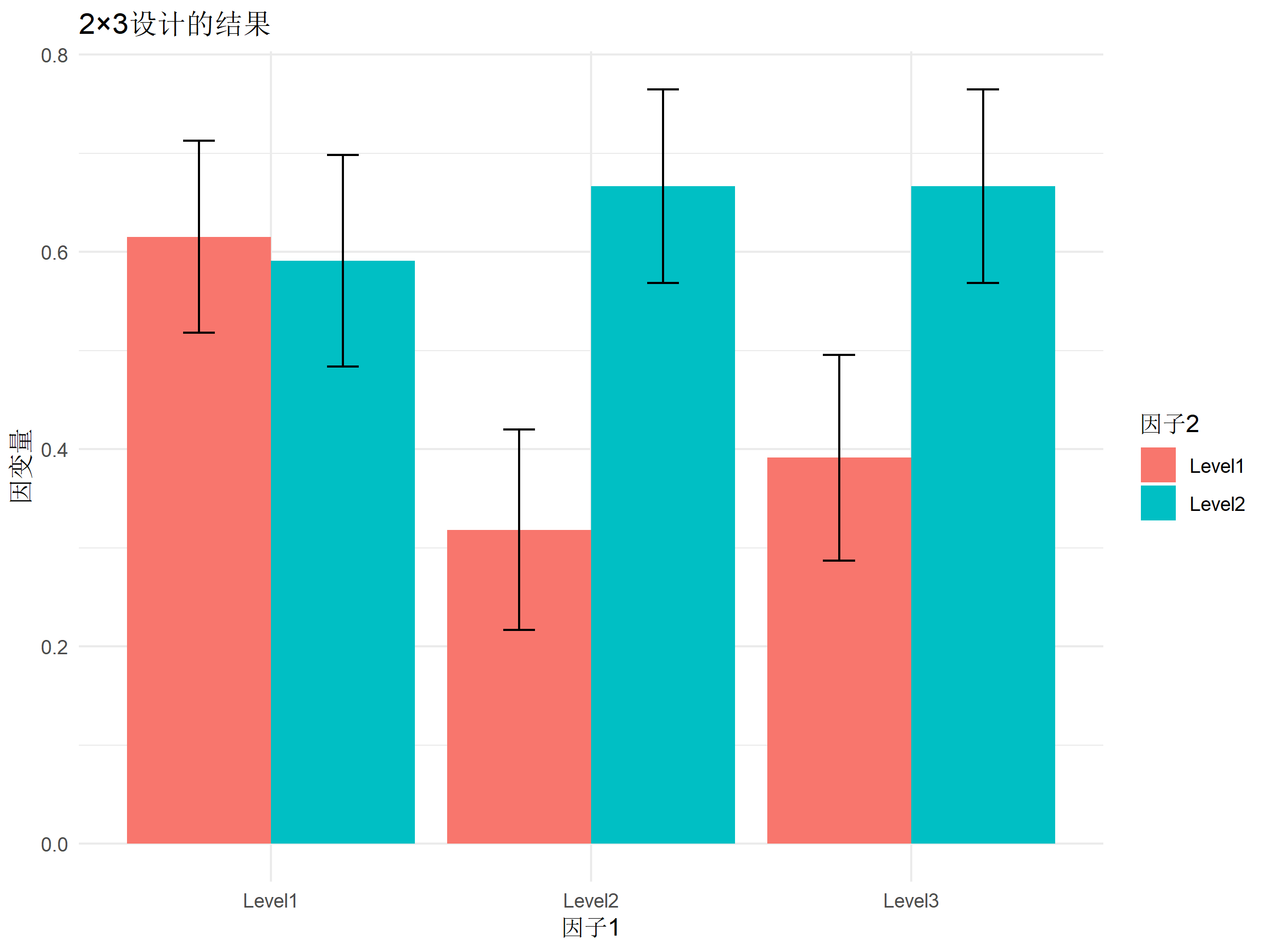

Supplement: Supplementary file 1 [file jemr-18-00033-s001.zip › local/2_probability/result_plot.png]

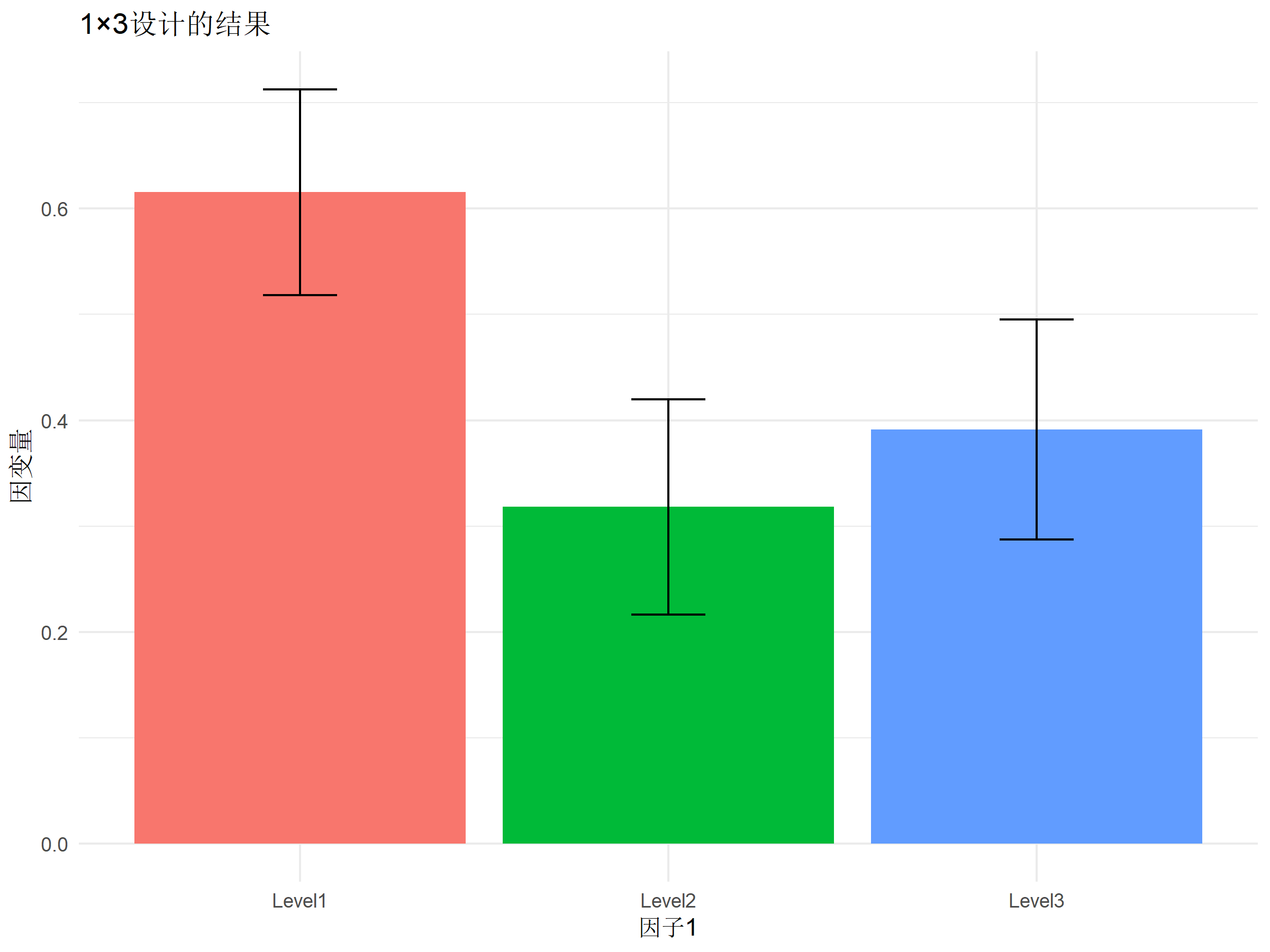

Supplement: Supplementary file 1 [file jemr-18-00033-s001.zip › local/2_probability2/result_plot.png]

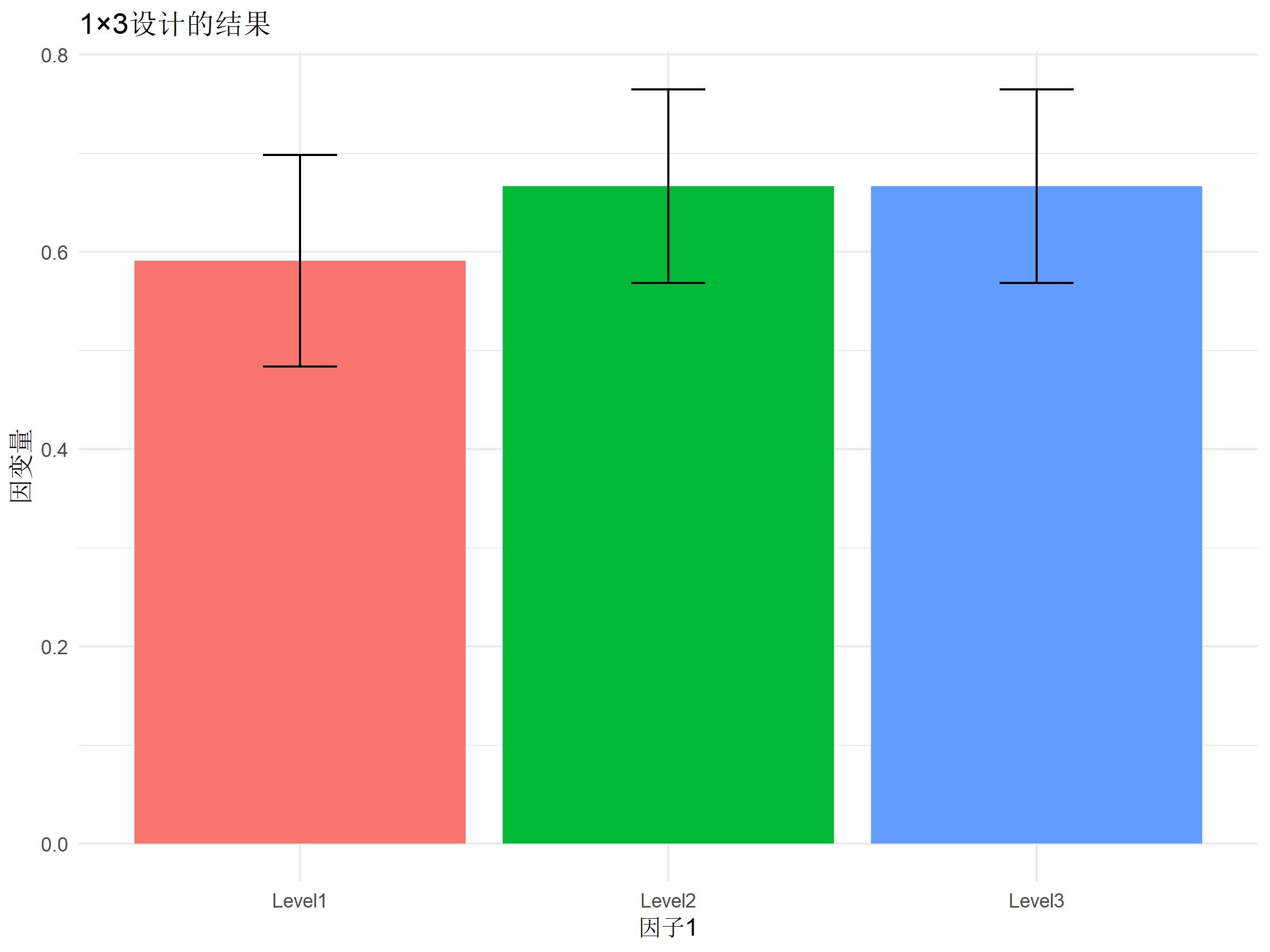

Supplement: Supplementary file 1 [file jemr-18-00033-s001.zip › local/2_probability3/result_plot.png]

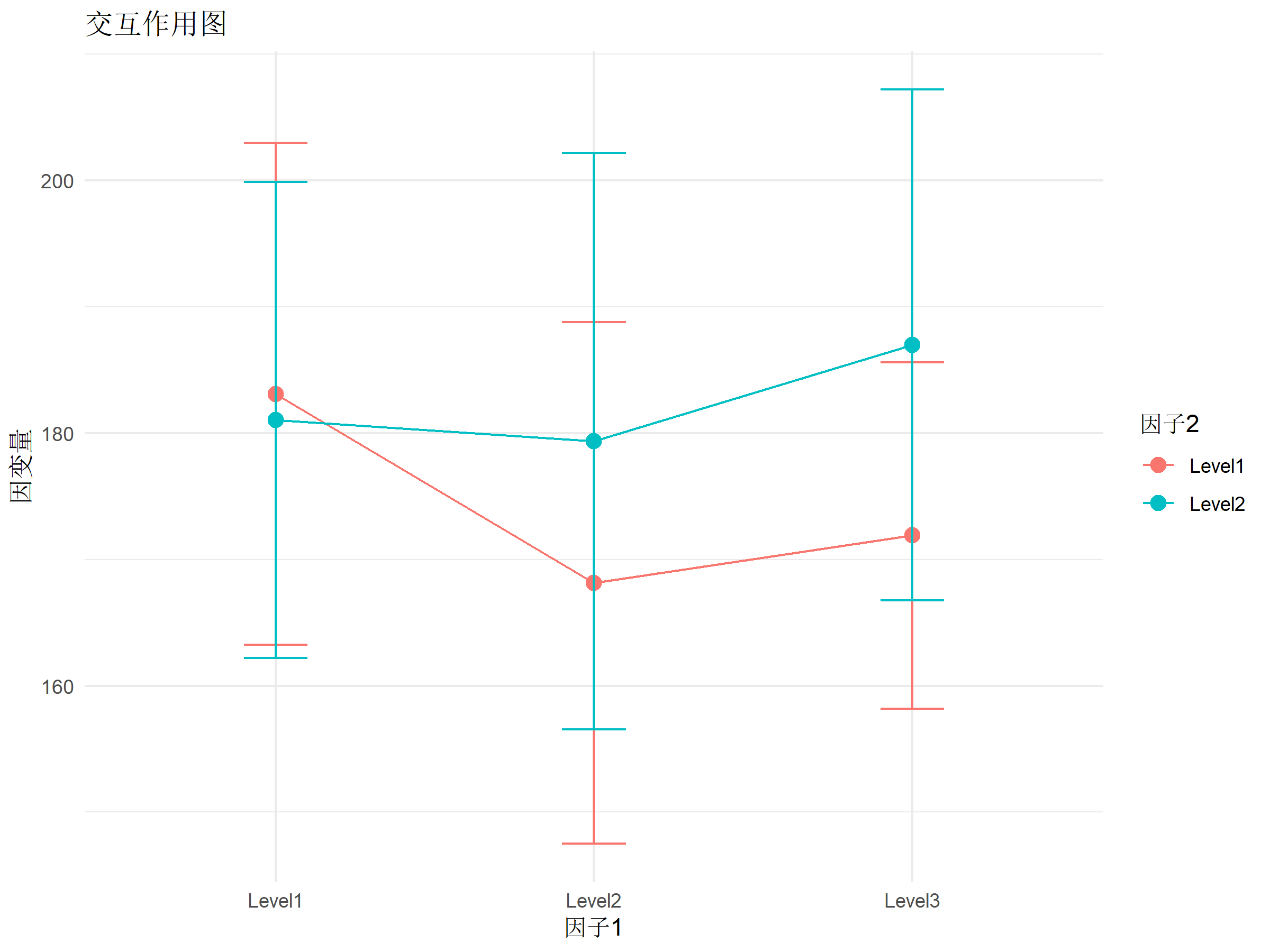

Supplement: Supplementary file 1 [file jemr-18-00033-s001.zip › local/Duration_of_first_fixation/interaction_plot.png]

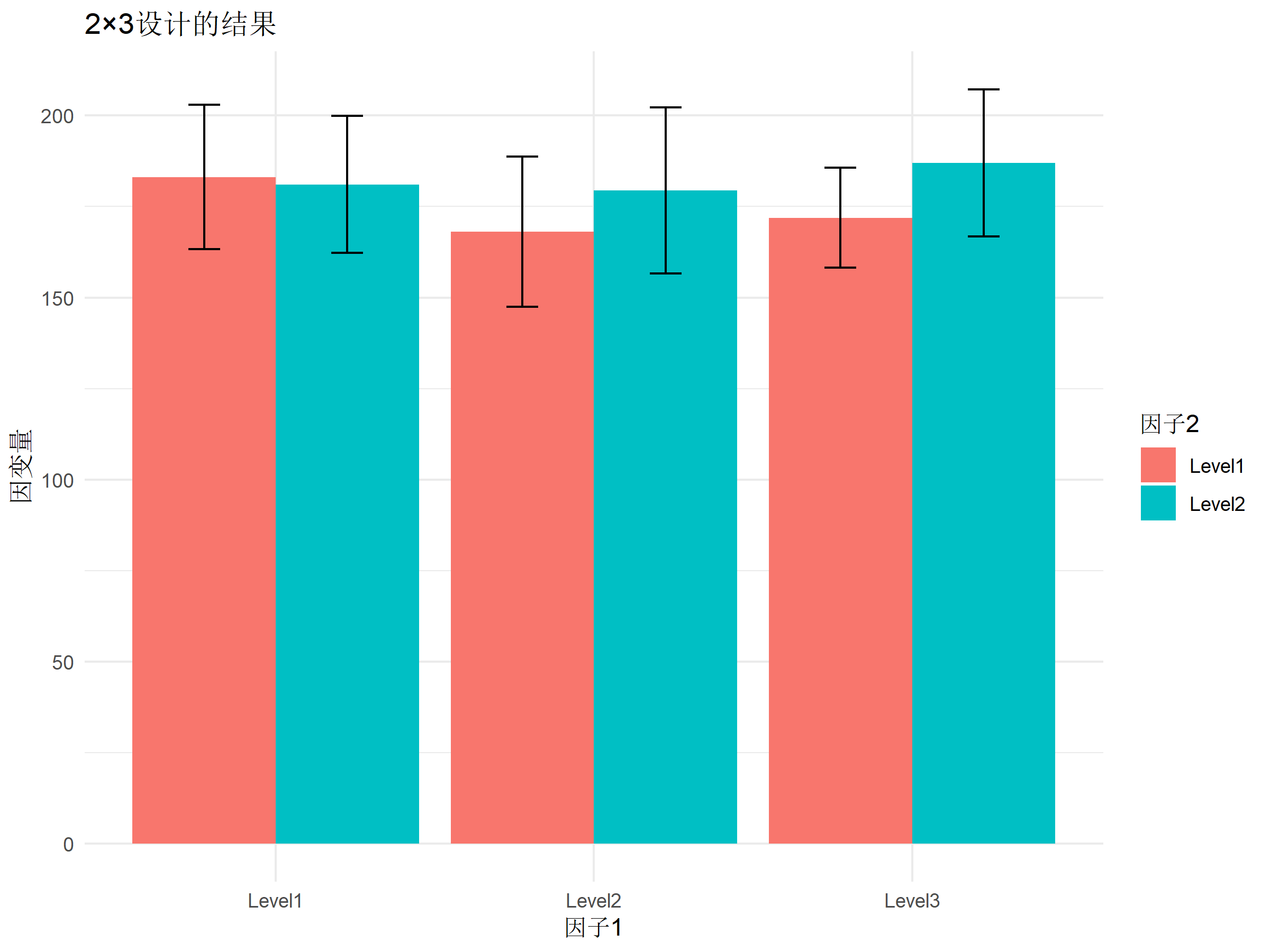

Supplement: Supplementary file 1 [file jemr-18-00033-s001.zip › local/Duration_of_first_fixation/result_plot.png]

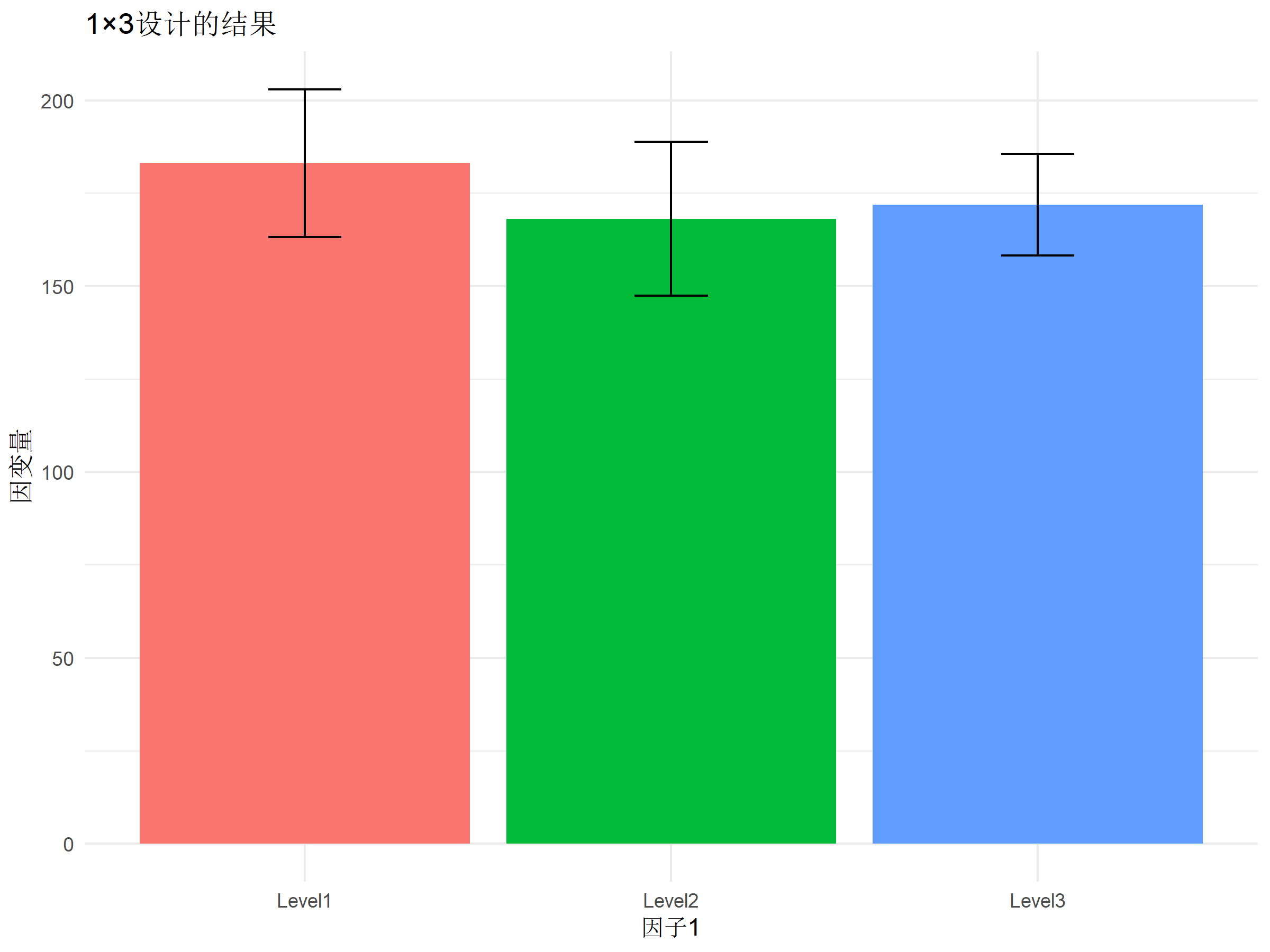

Supplement: Supplementary file 1 [file jemr-18-00033-s001.zip › local/Duration_of_first_fixation2/result_plot.png]

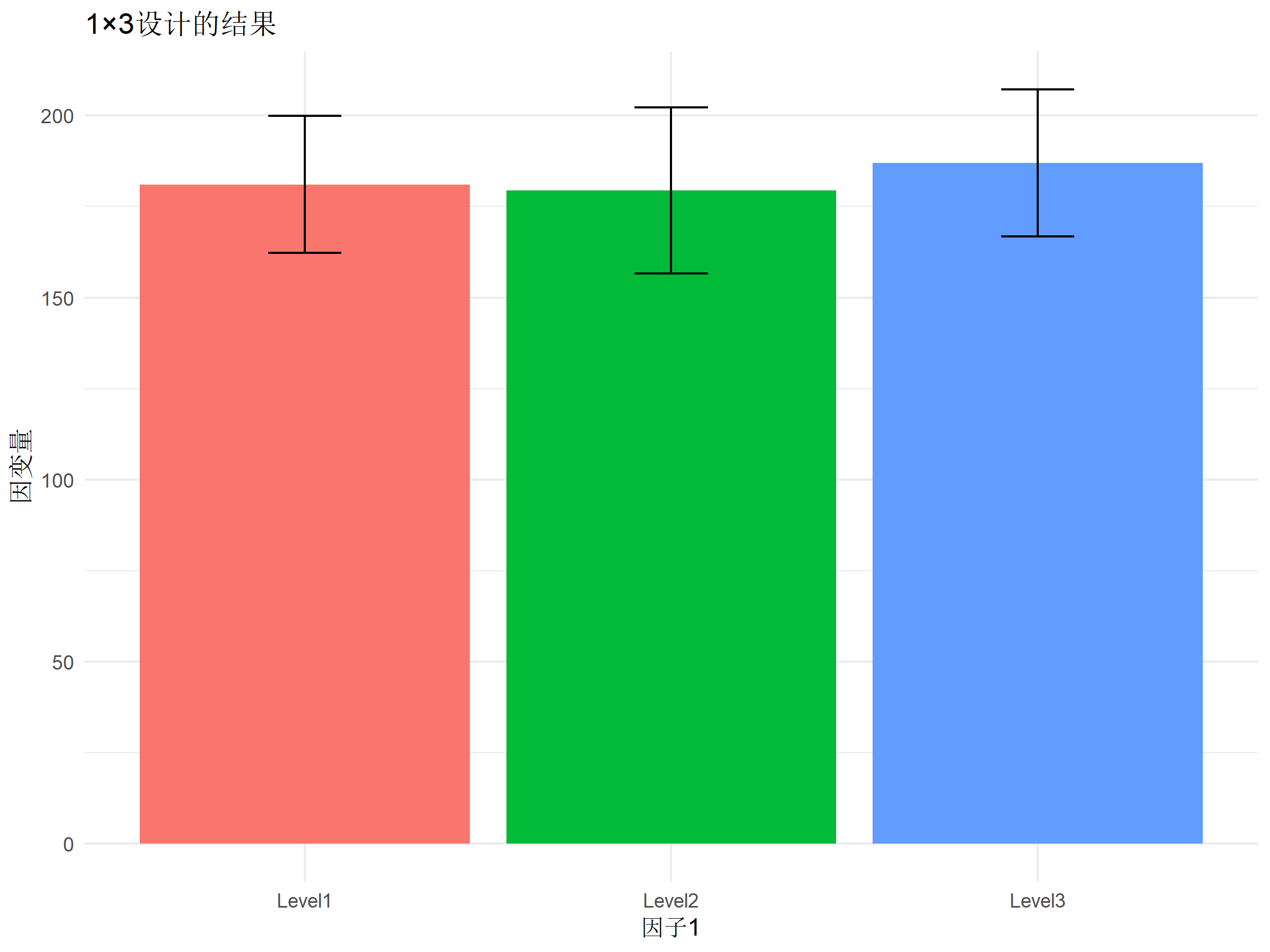

Supplement: Supplementary file 1 [file jemr-18-00033-s001.zip › local/Duration_of_first_fixation3/result_plot.png]

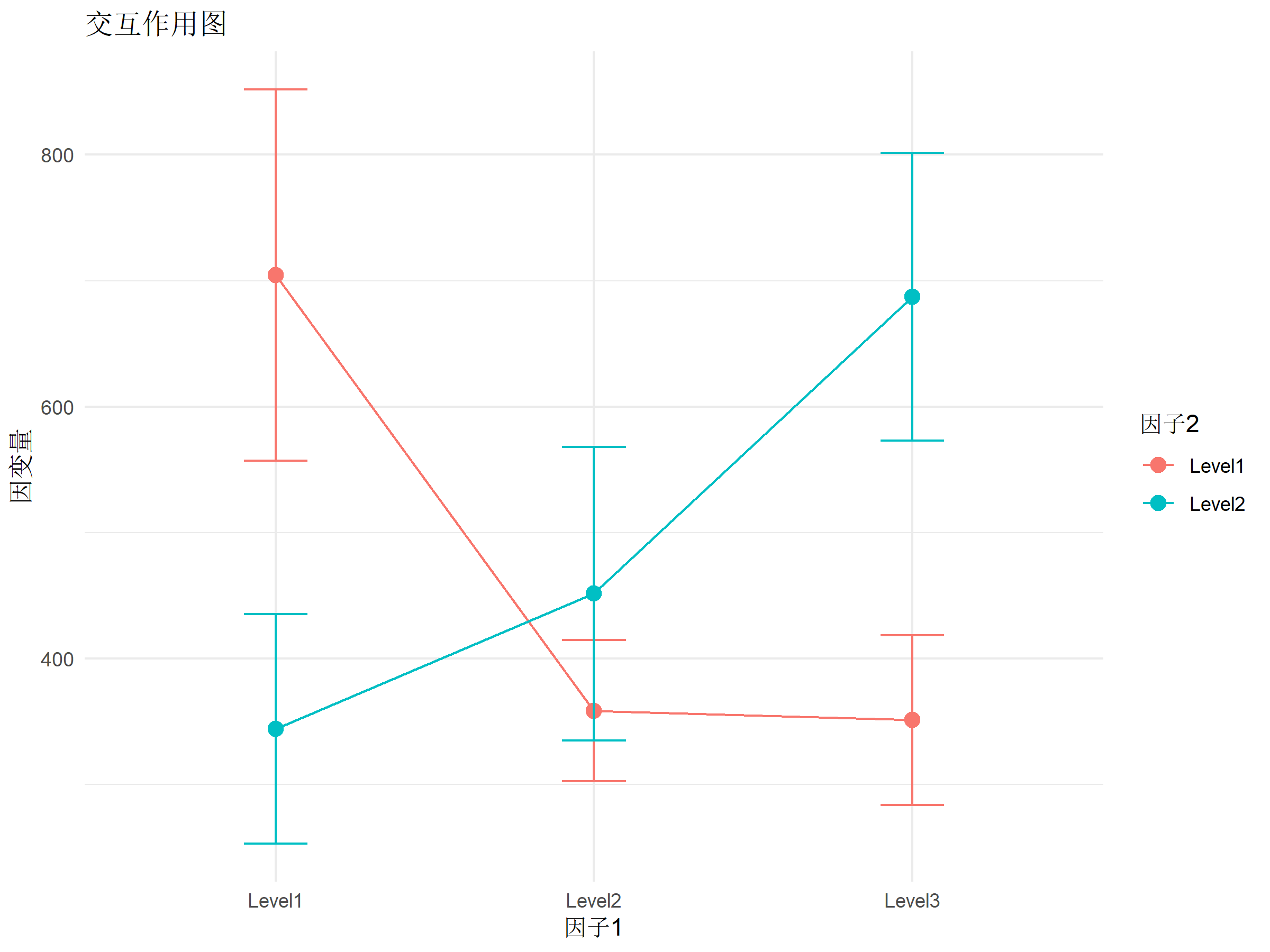

Supplement: Supplementary file 1 [file jemr-18-00033-s001.zip › local/gaze/interaction_plot.png]

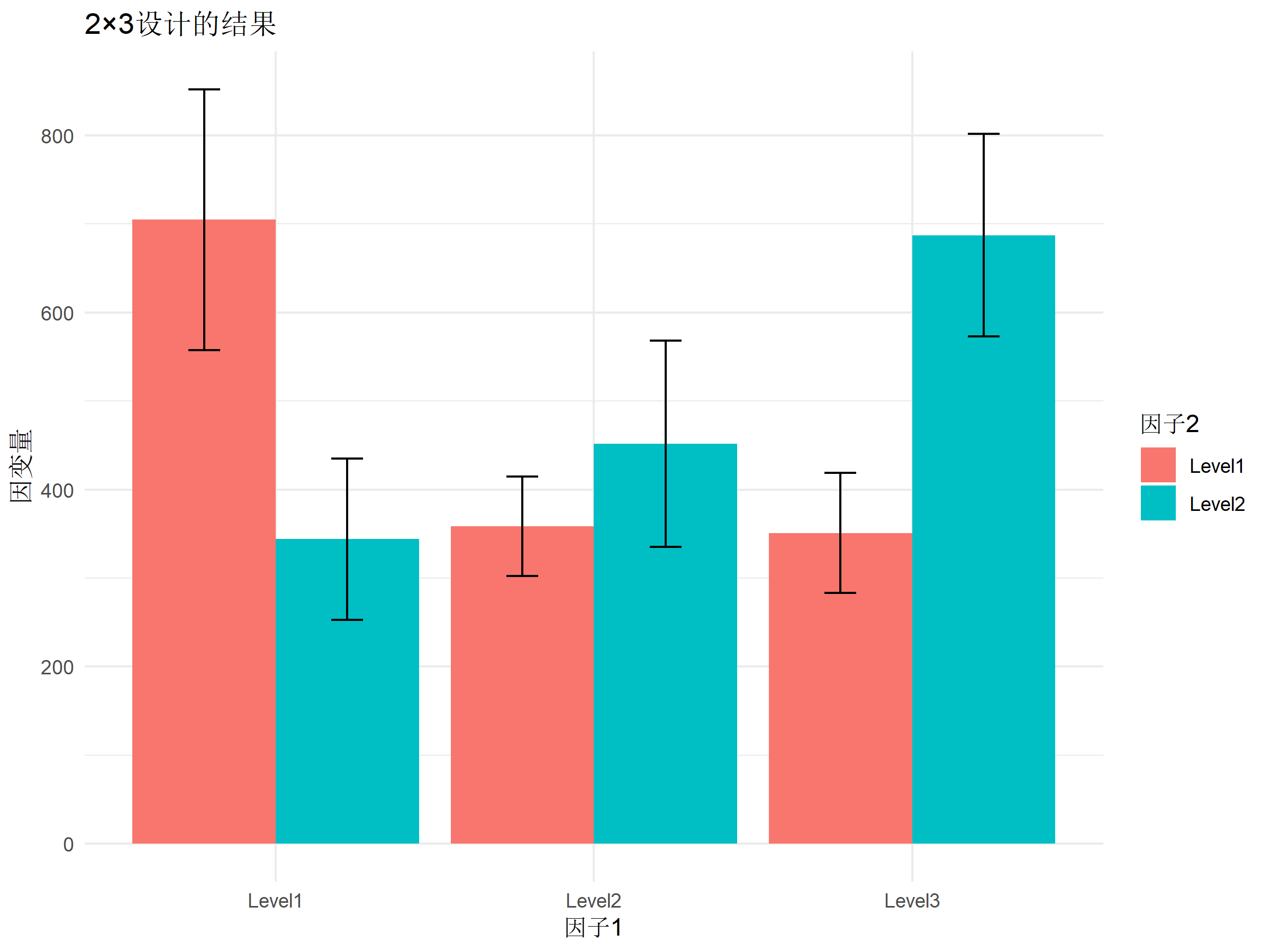

Supplement: Supplementary file 1 [file jemr-18-00033-s001.zip › local/gaze/result_plot.png]

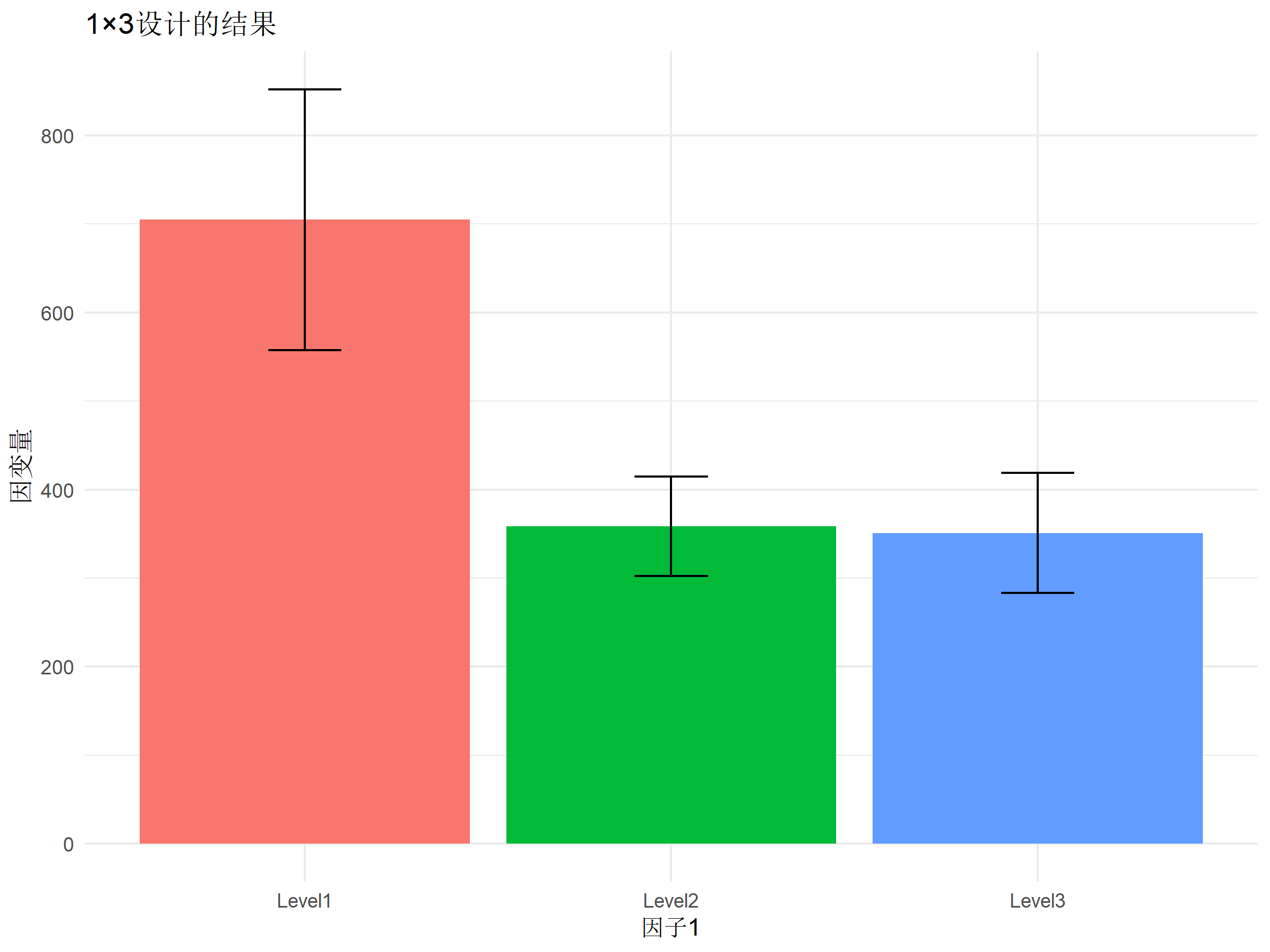

Supplement: Supplementary file 1 [file jemr-18-00033-s001.zip › local/gaze2/result_plot.png]

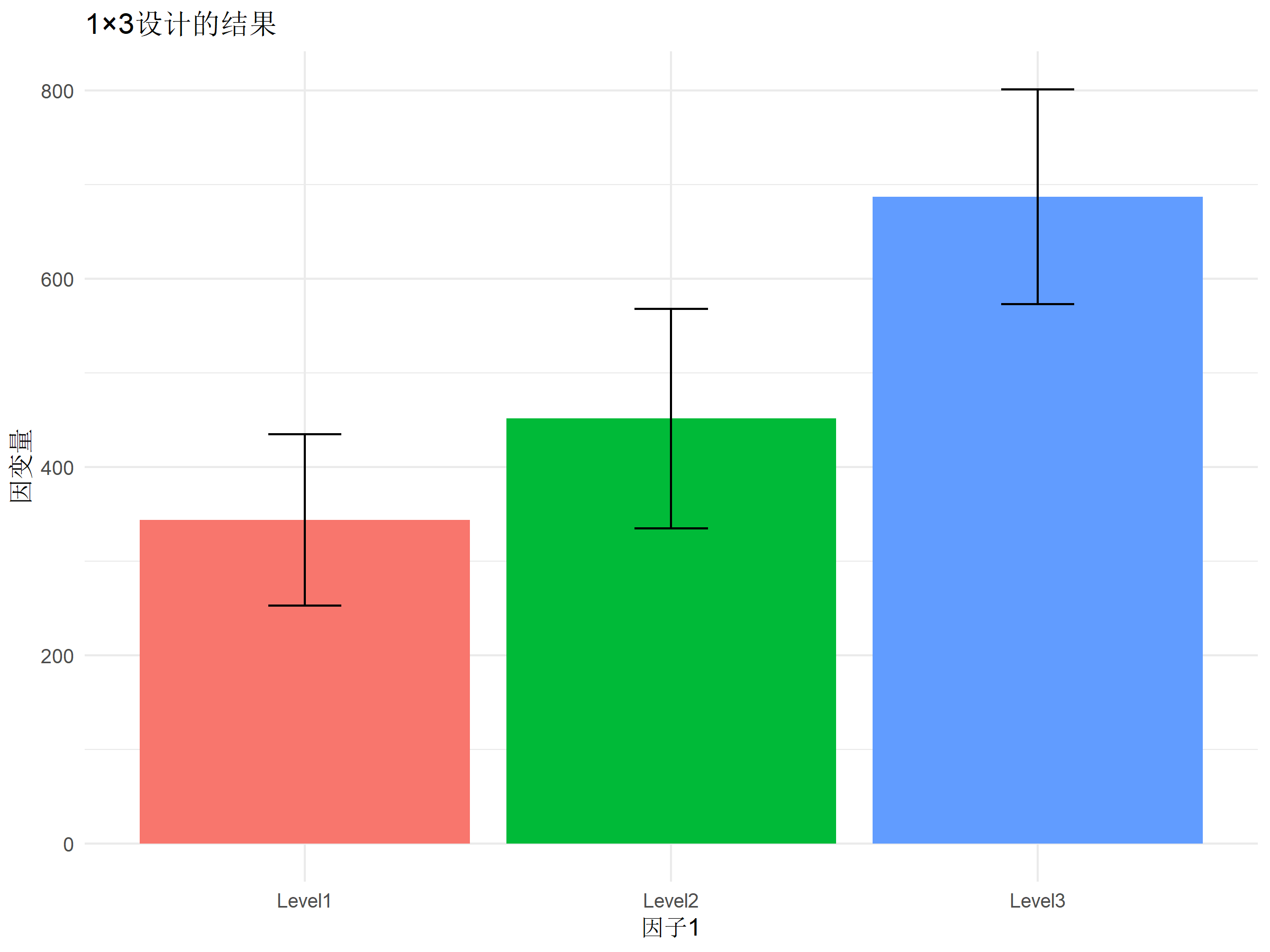

Supplement: Supplementary file 1 [file jemr-18-00033-s001.zip › local/gaze3/result_plot.png]

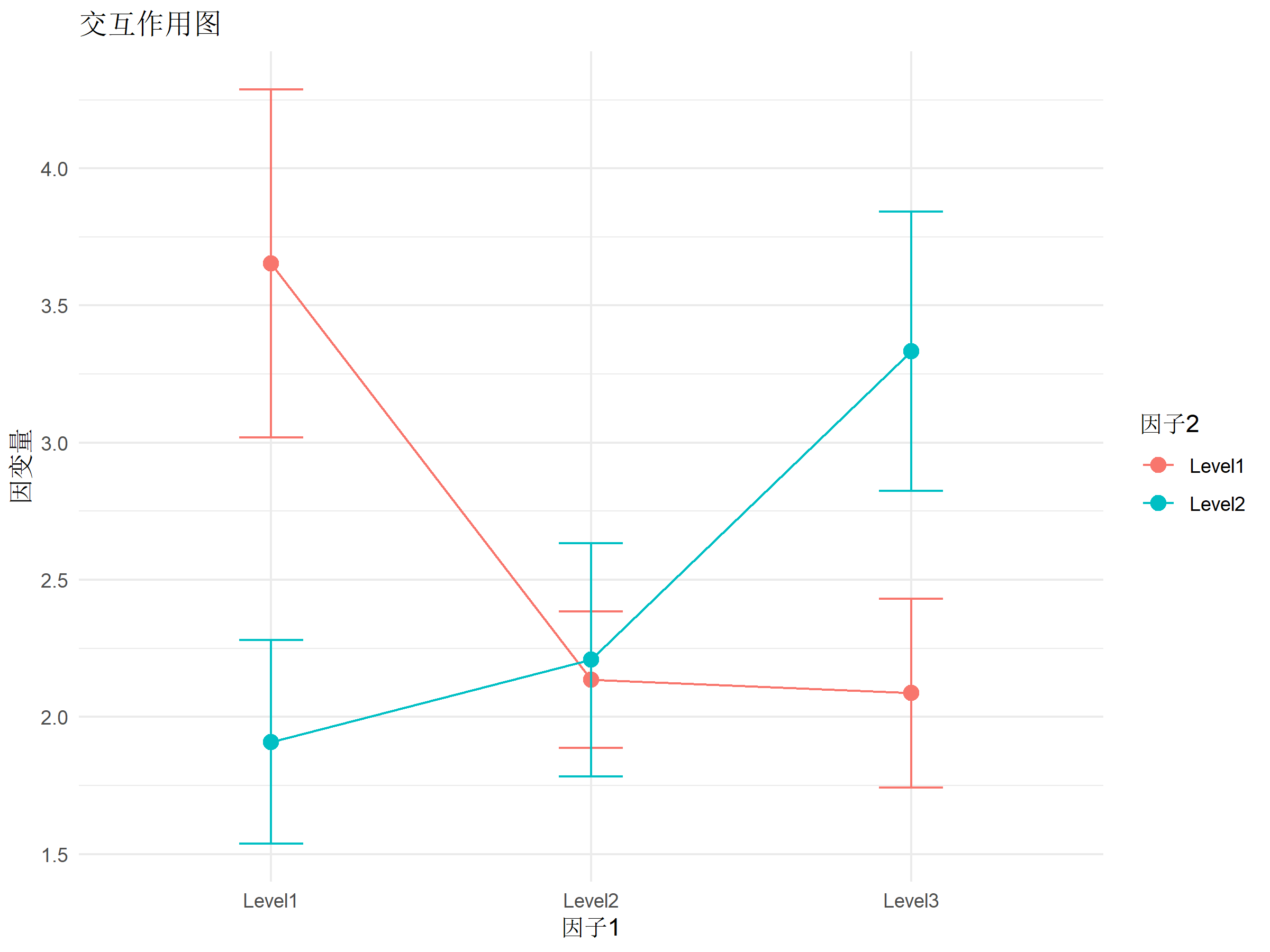

Supplement: Supplementary file 1 [file jemr-18-00033-s001.zip › local/gaze_count/interaction_plot.png]

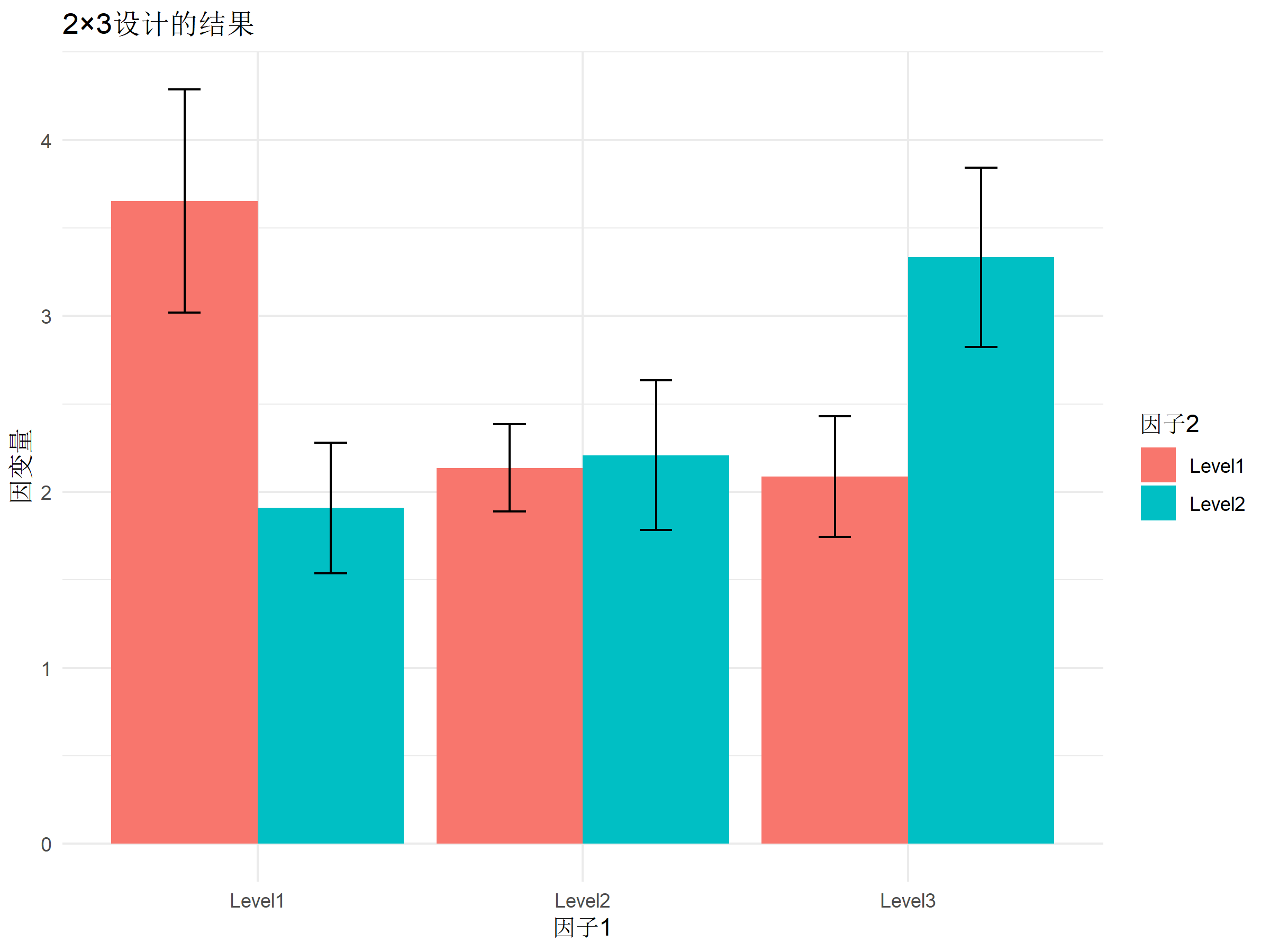

Supplement: Supplementary file 1 [file jemr-18-00033-s001.zip › local/gaze_count/result_plot.png]

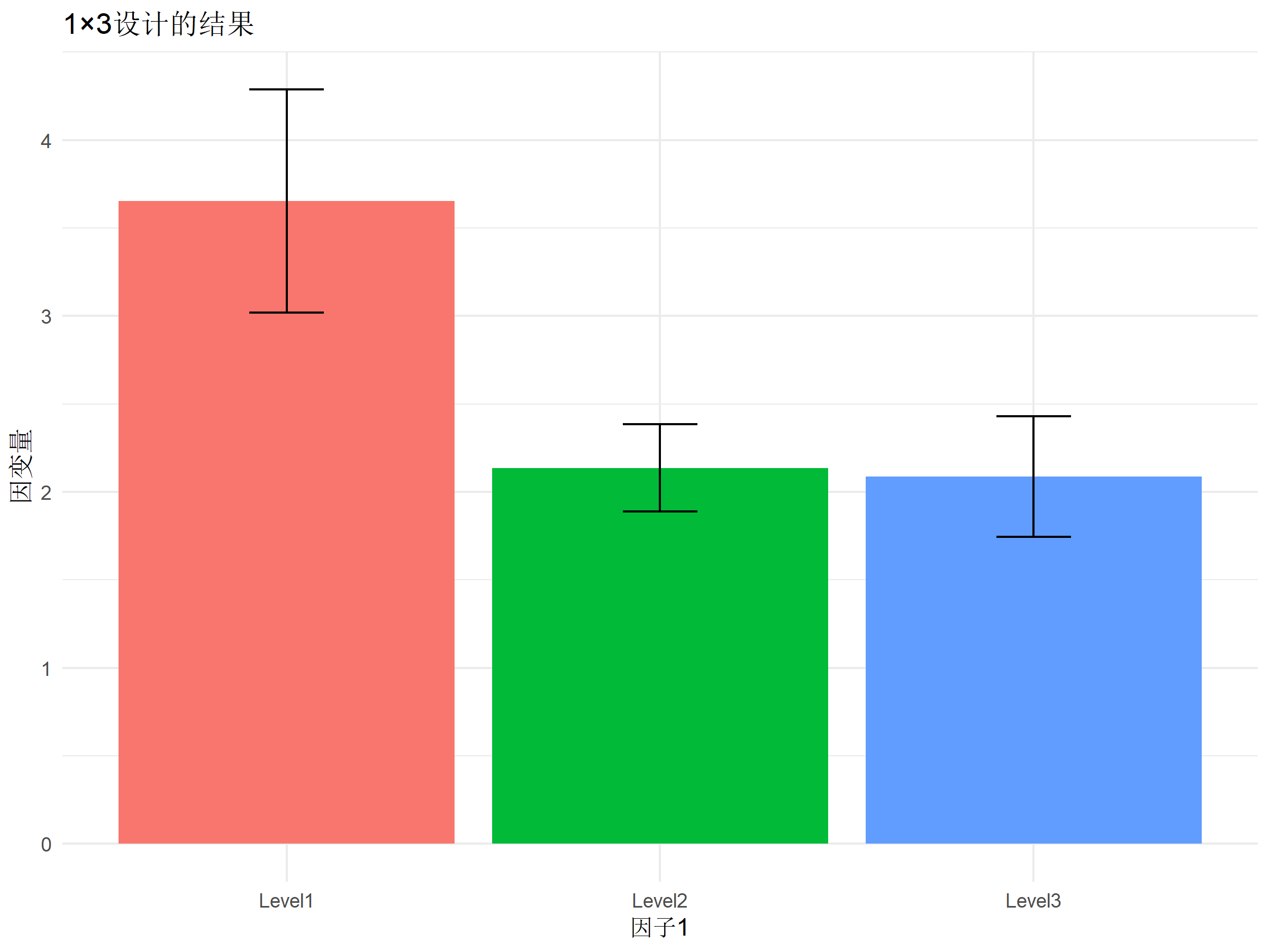

Supplement: Supplementary file 1 [file jemr-18-00033-s001.zip › local/gaze_count2/result_plot.png]

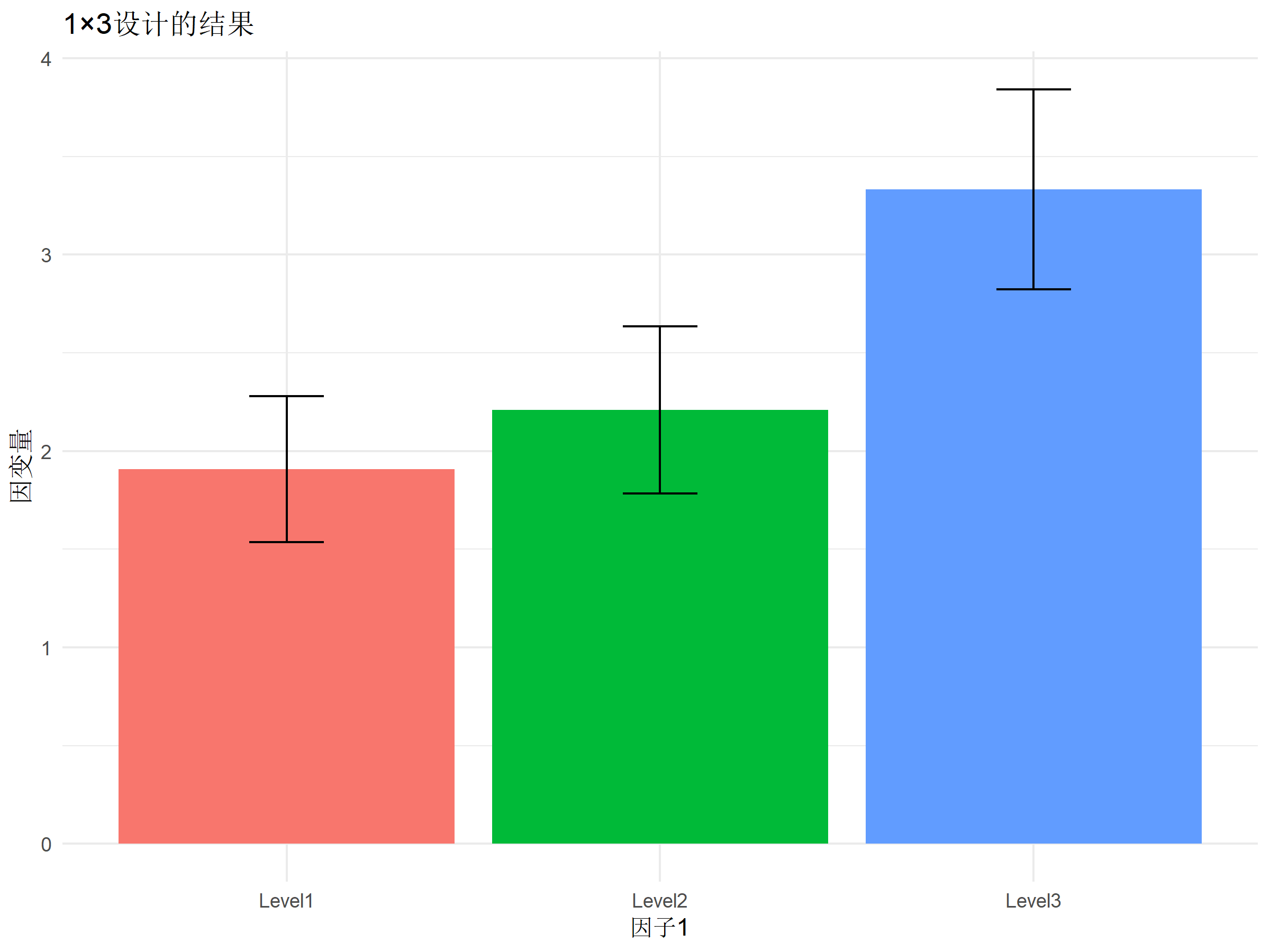

Supplement: Supplementary file 1 [file jemr-18-00033-s001.zip › local/gaze_count3/result_plot.png]

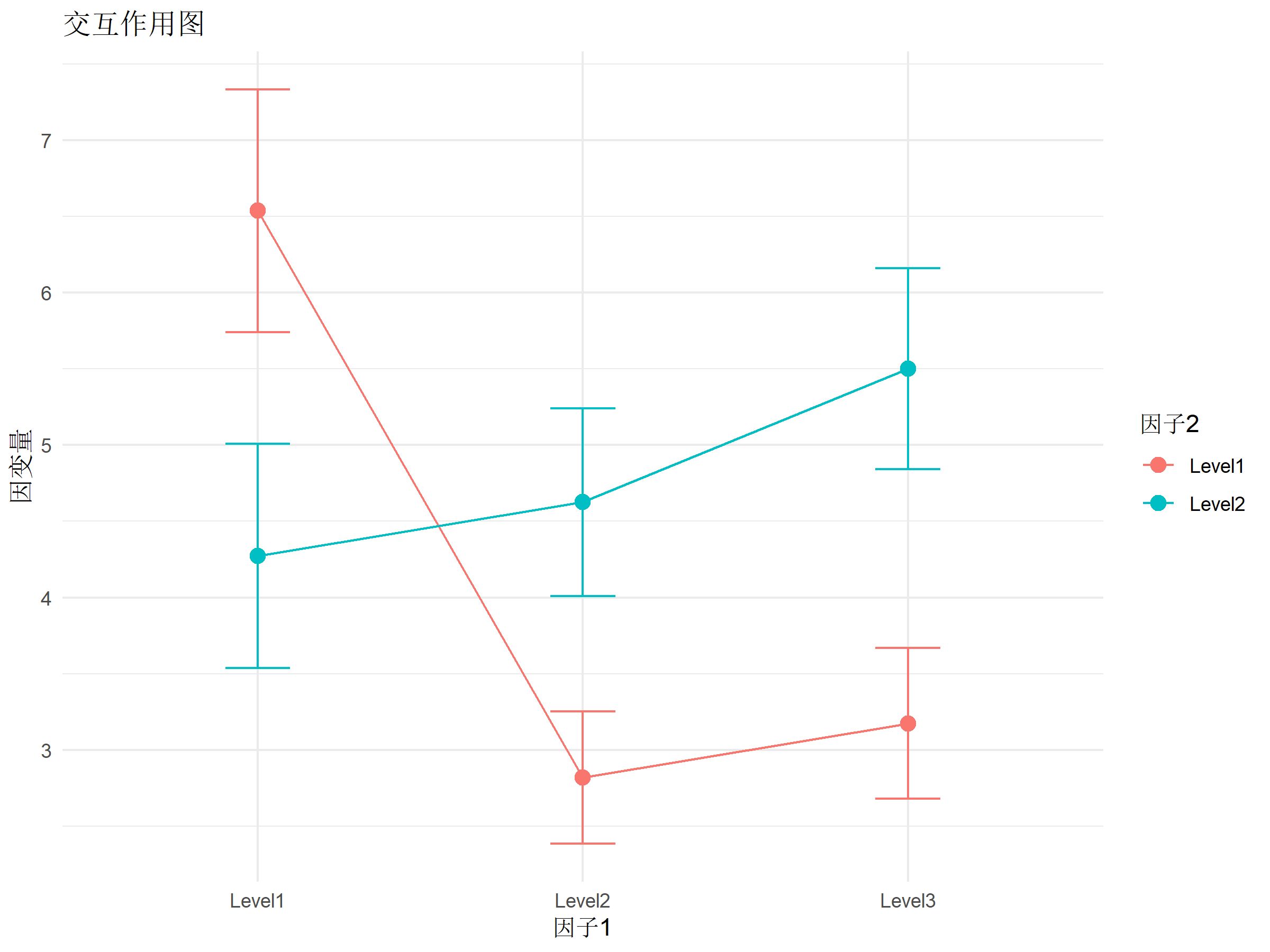

Supplement: Supplementary file 1 [file jemr-18-00033-s001.zip › local/Number_of_fixations/interaction_plot.png]

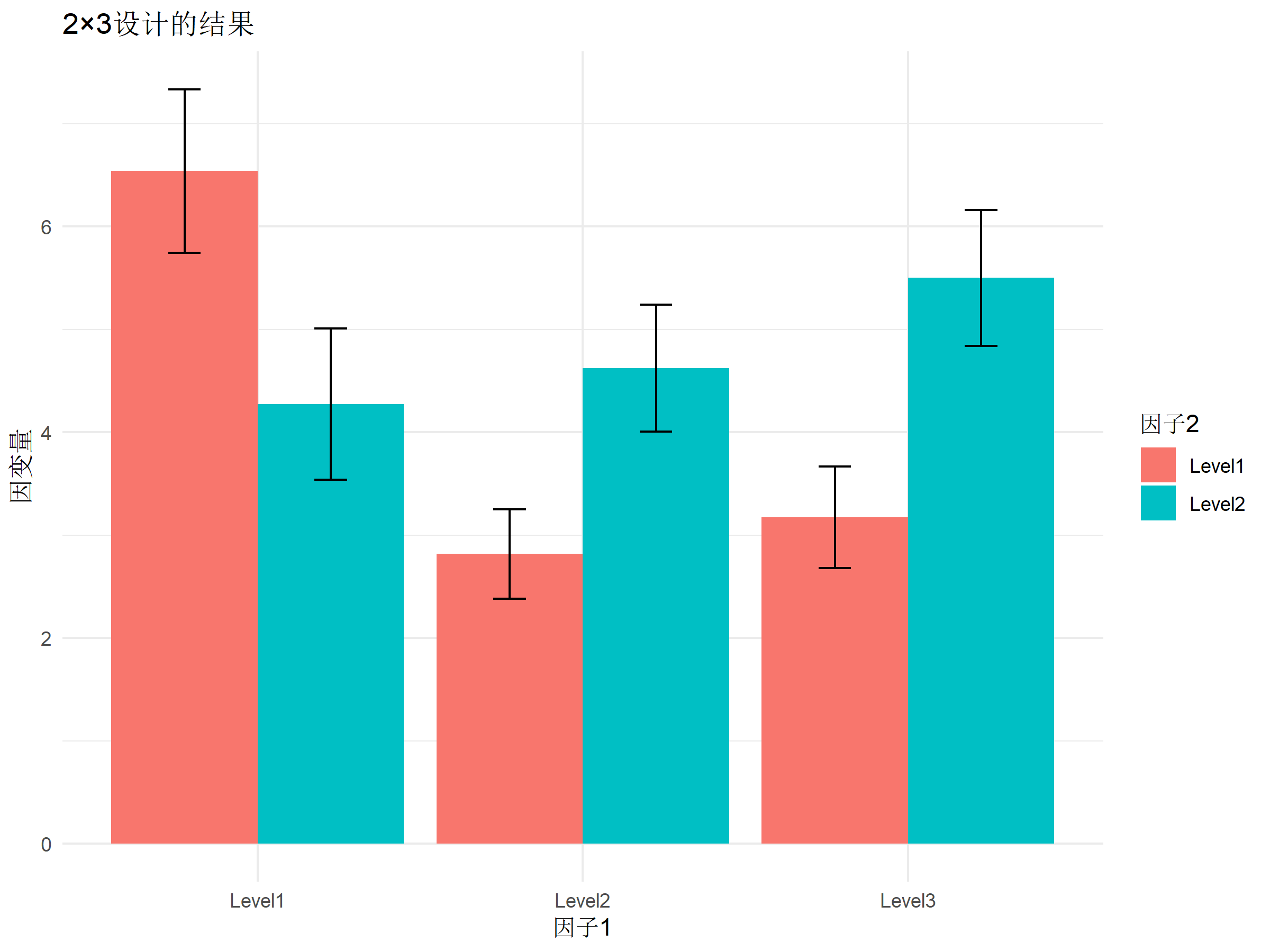

Supplement: Supplementary file 1 [file jemr-18-00033-s001.zip › local/Number_of_fixations/result_plot.png]

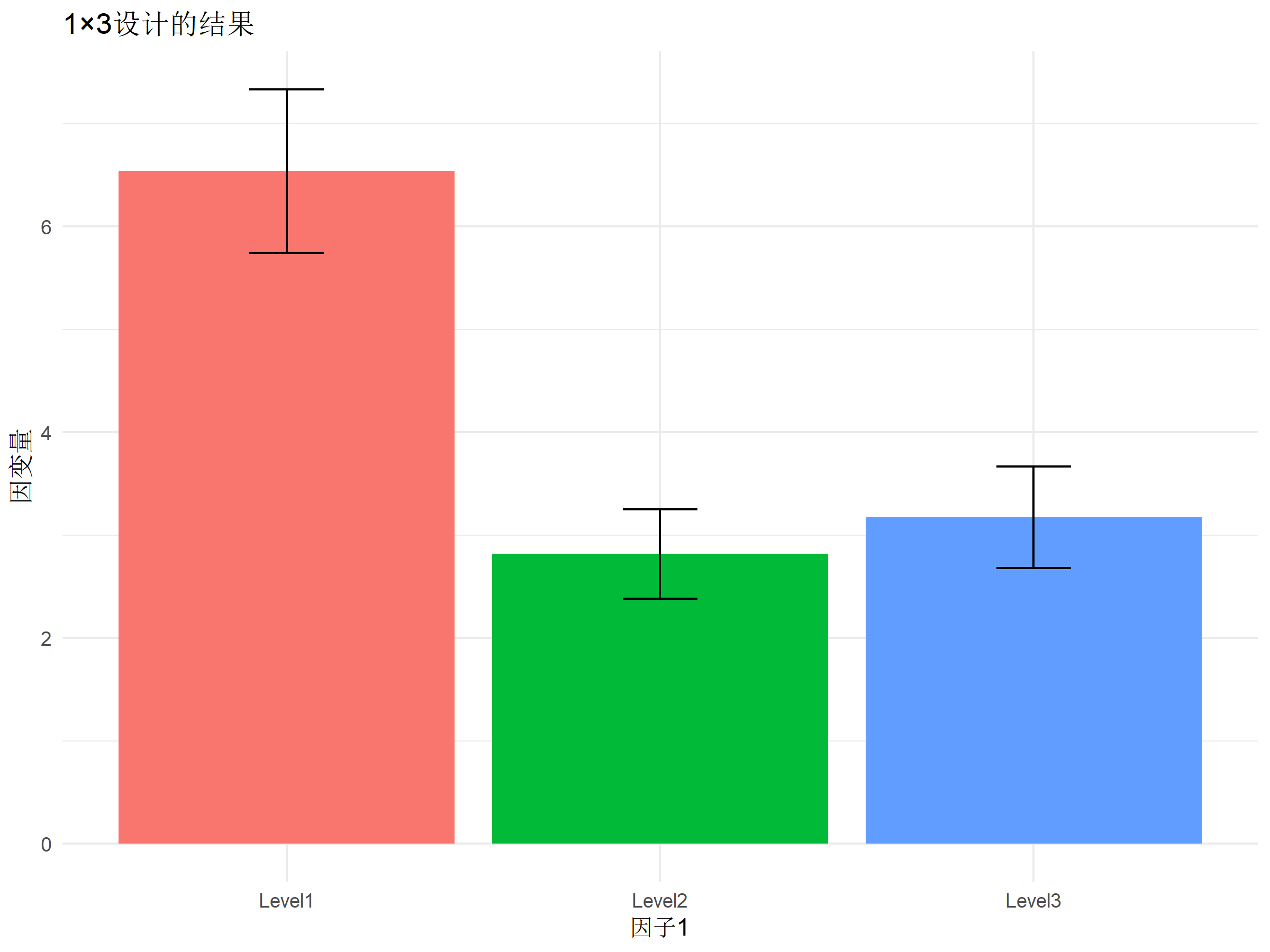

Supplement: Supplementary file 1 [file jemr-18-00033-s001.zip › local/Number_of_fixations2/result_plot.png]

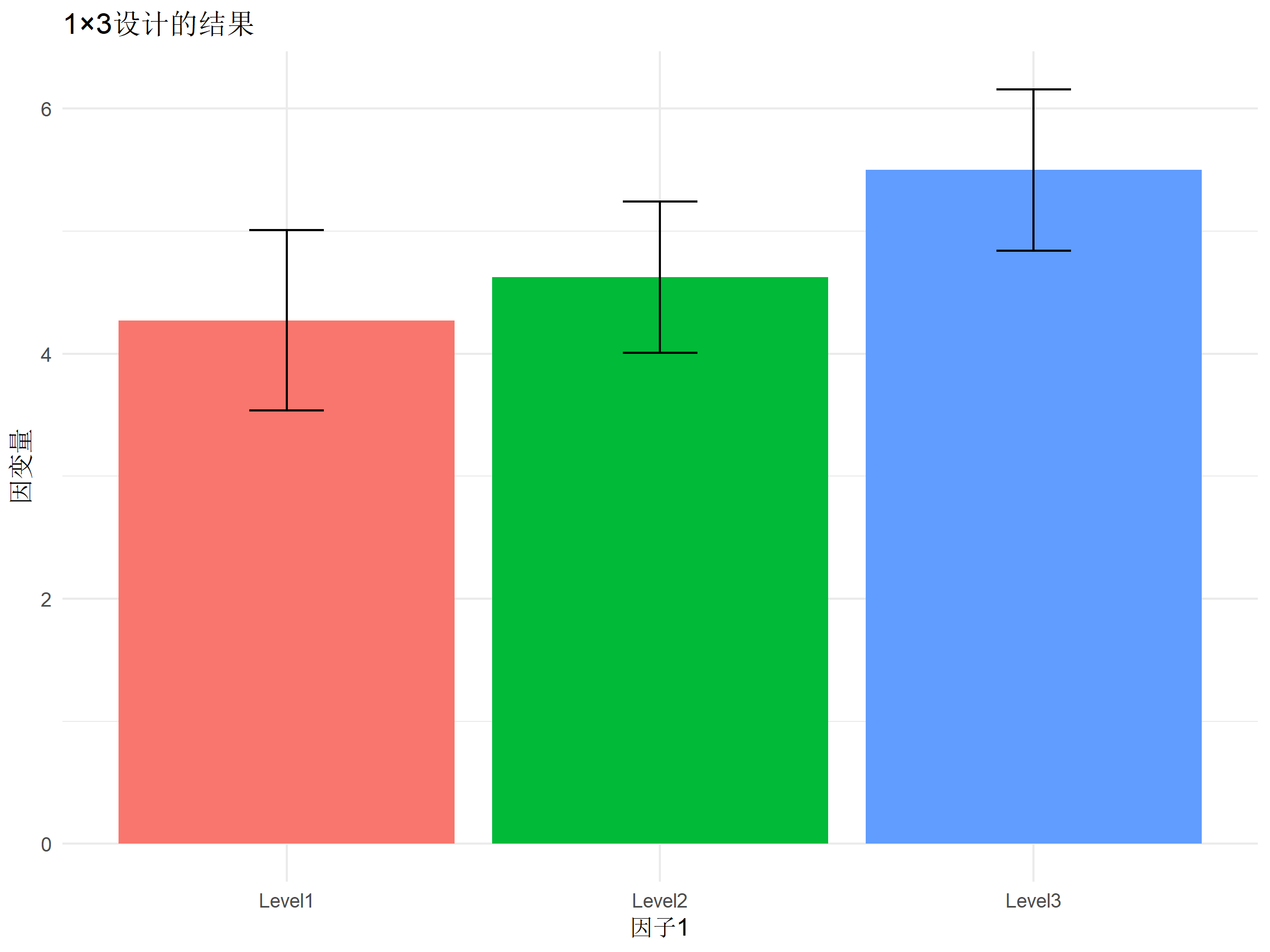

Supplement: Supplementary file 1 [file jemr-18-00033-s001.zip › local/Number_of_fixations3/result_plot.png]

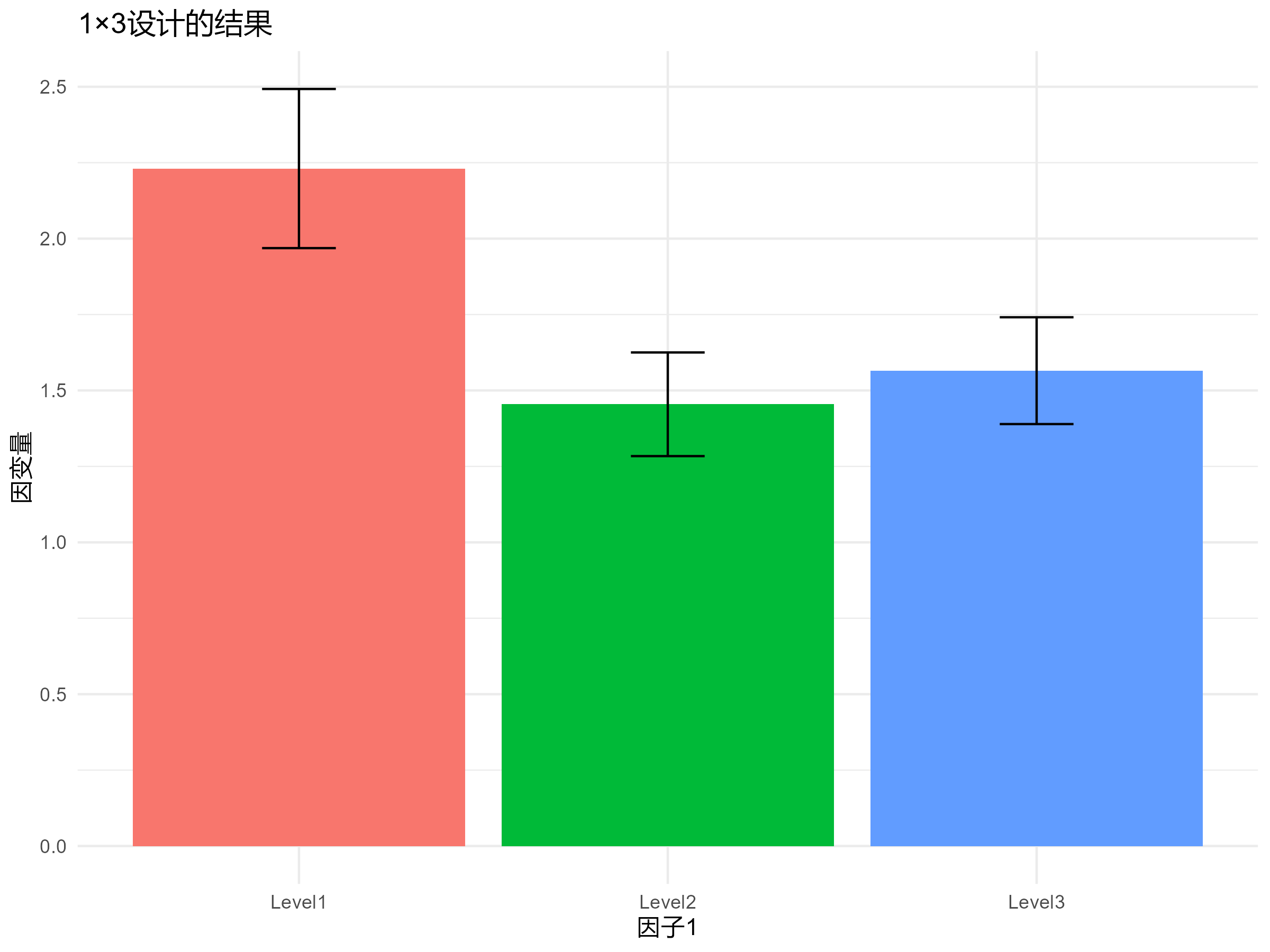

Supplement: Supplementary file 1 [file jemr-18-00033-s001.zip › local/number_of_visit/result_plot.png]

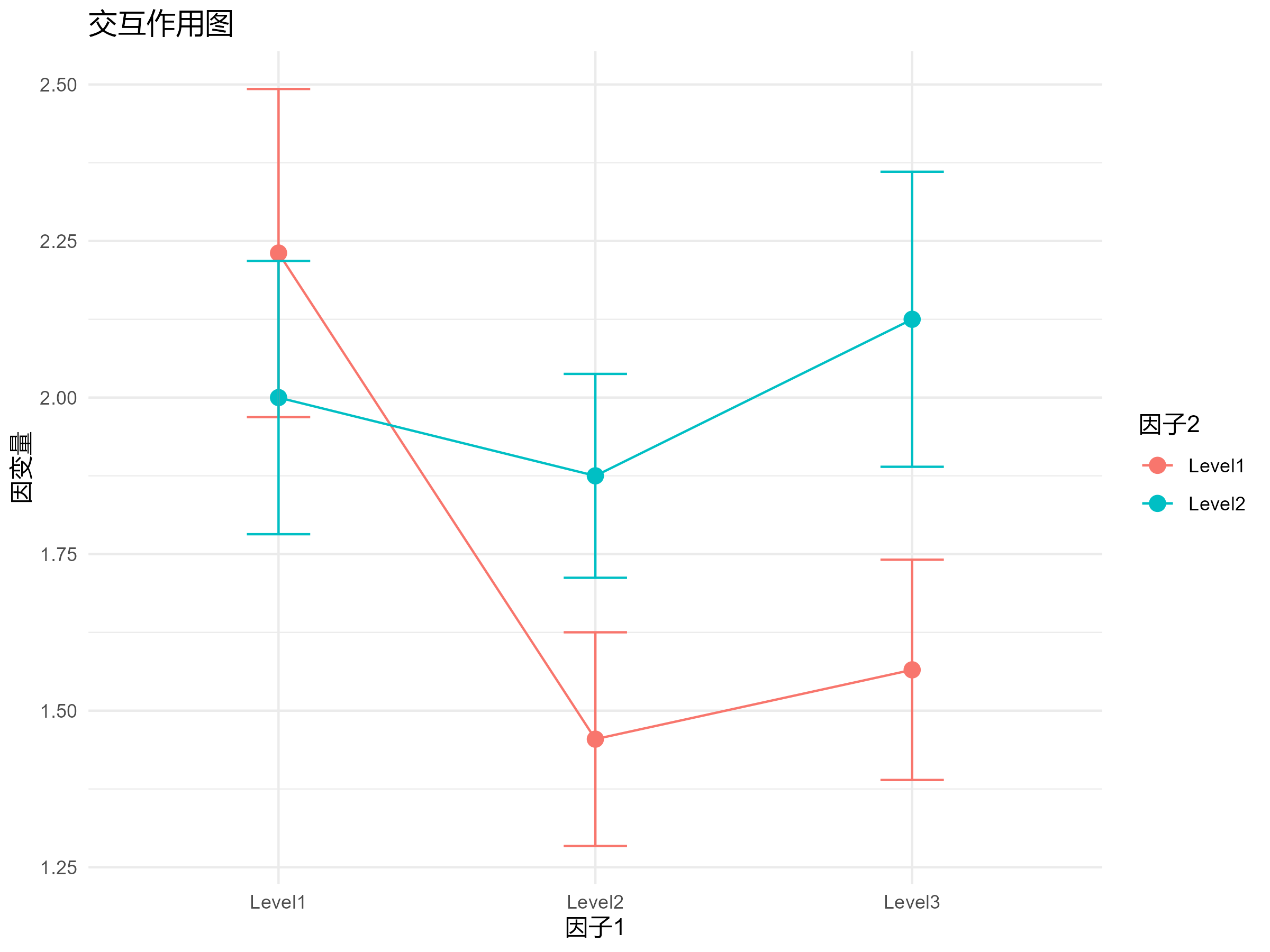

Supplement: Supplementary file 1 [file jemr-18-00033-s001.zip › local/Number_of_Visits/interaction_plot.png]

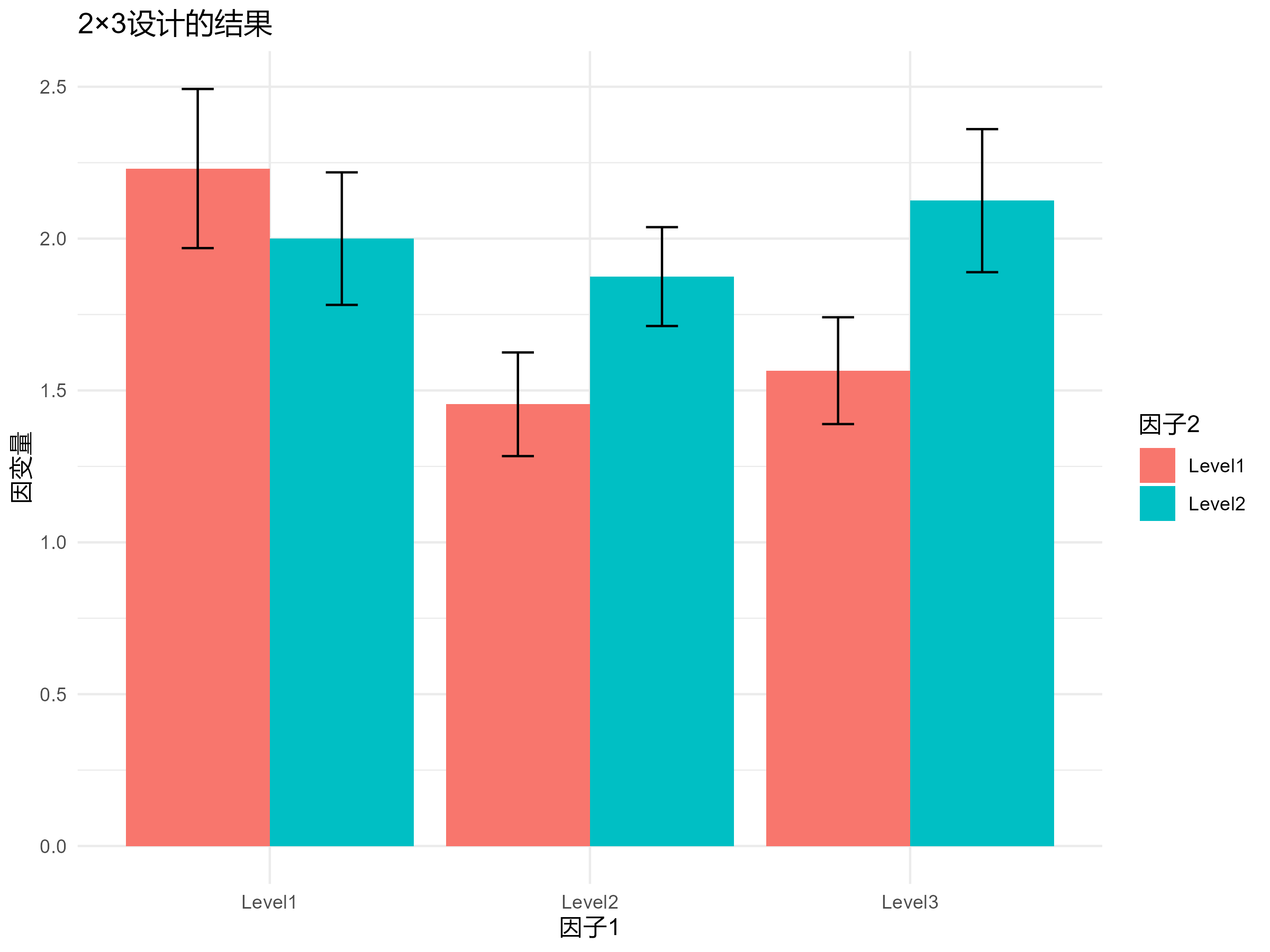

Supplement: Supplementary file 1 [file jemr-18-00033-s001.zip › local/Number_of_Visits/result_plot.png]

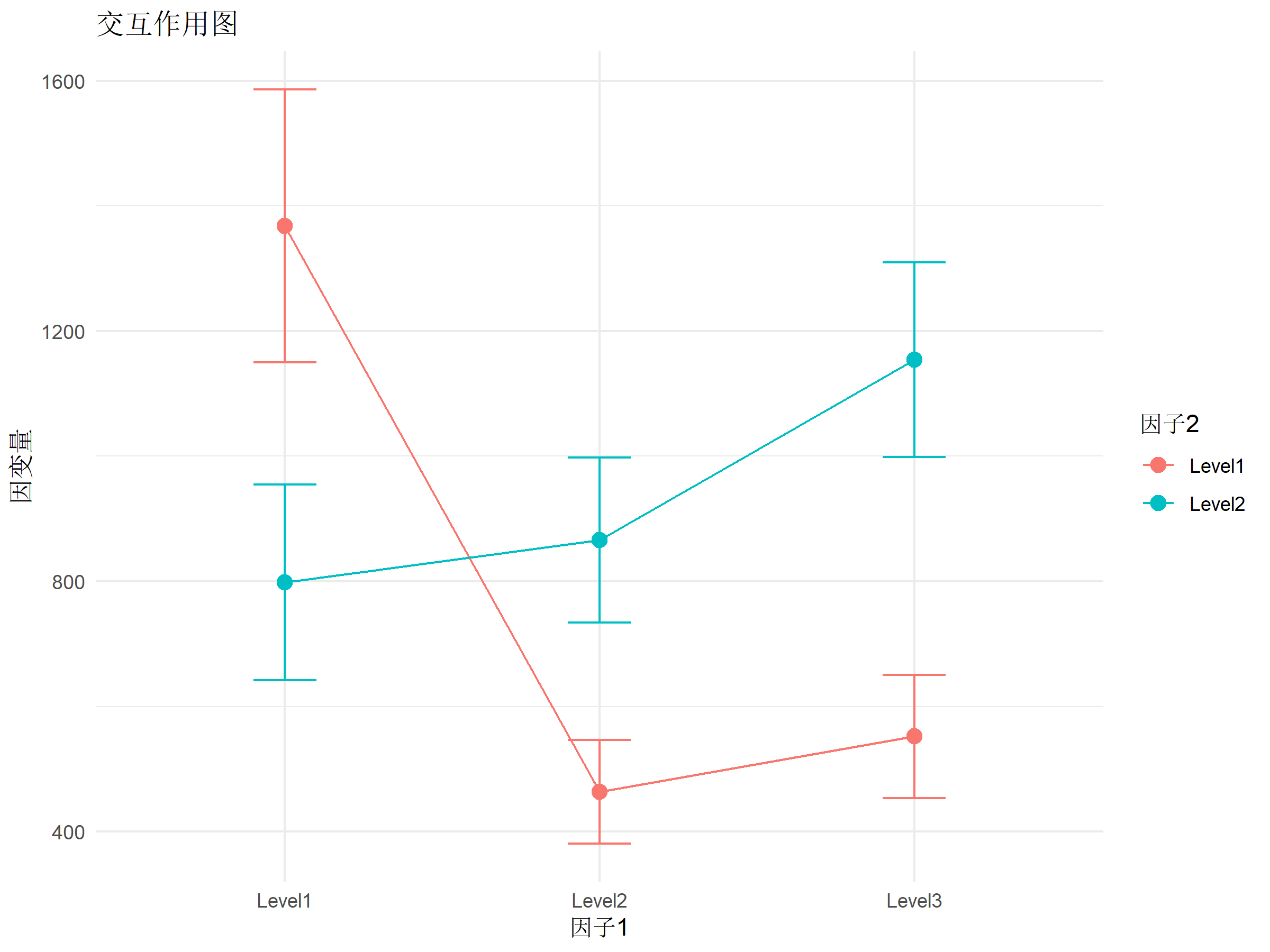

Supplement: Supplementary file 1 [file jemr-18-00033-s001.zip › local/Total_duration_of_fixations/interaction_plot.png]

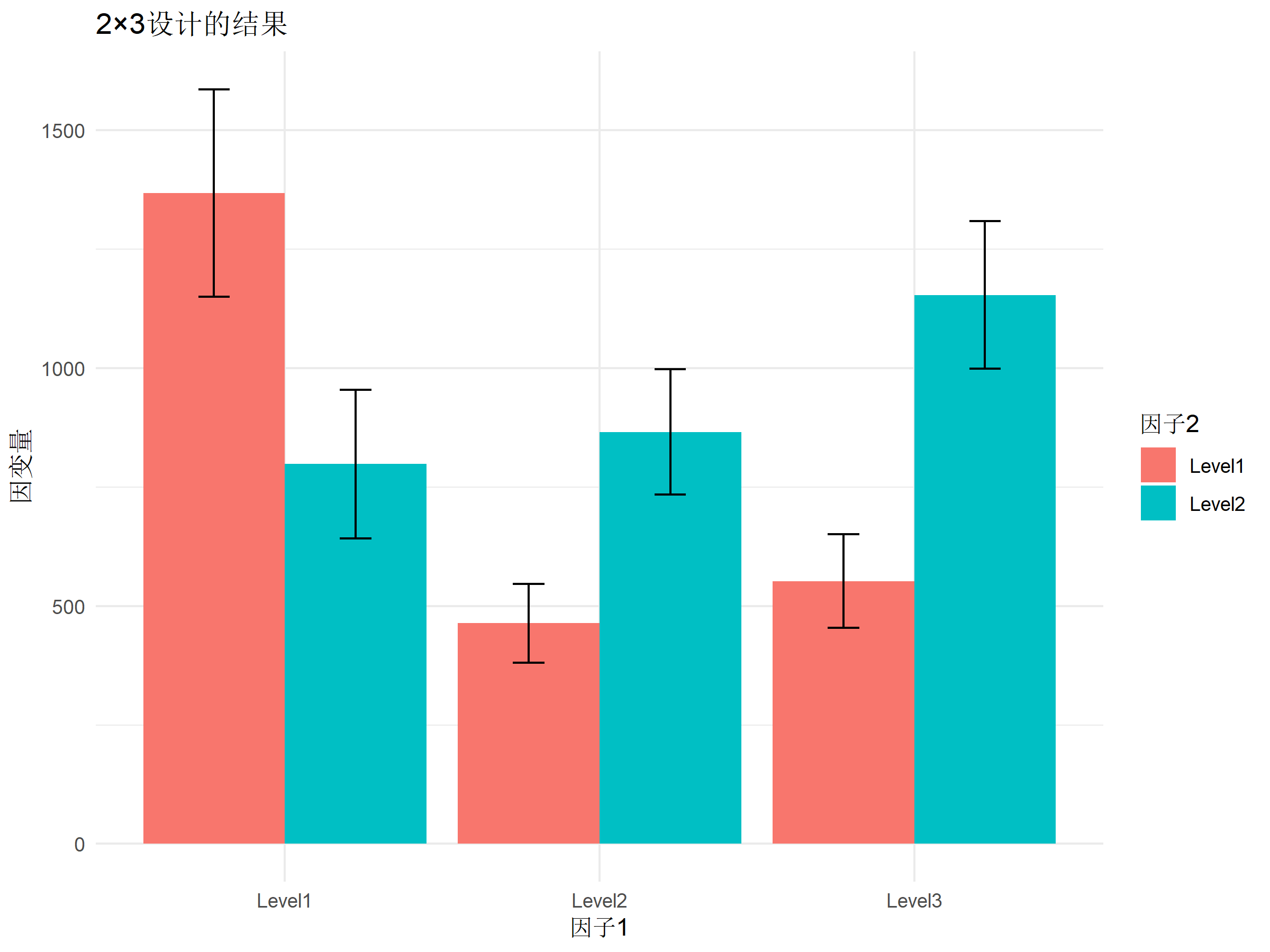

Supplement: Supplementary file 1 [file jemr-18-00033-s001.zip › local/Total_duration_of_fixations/result_plot.png]

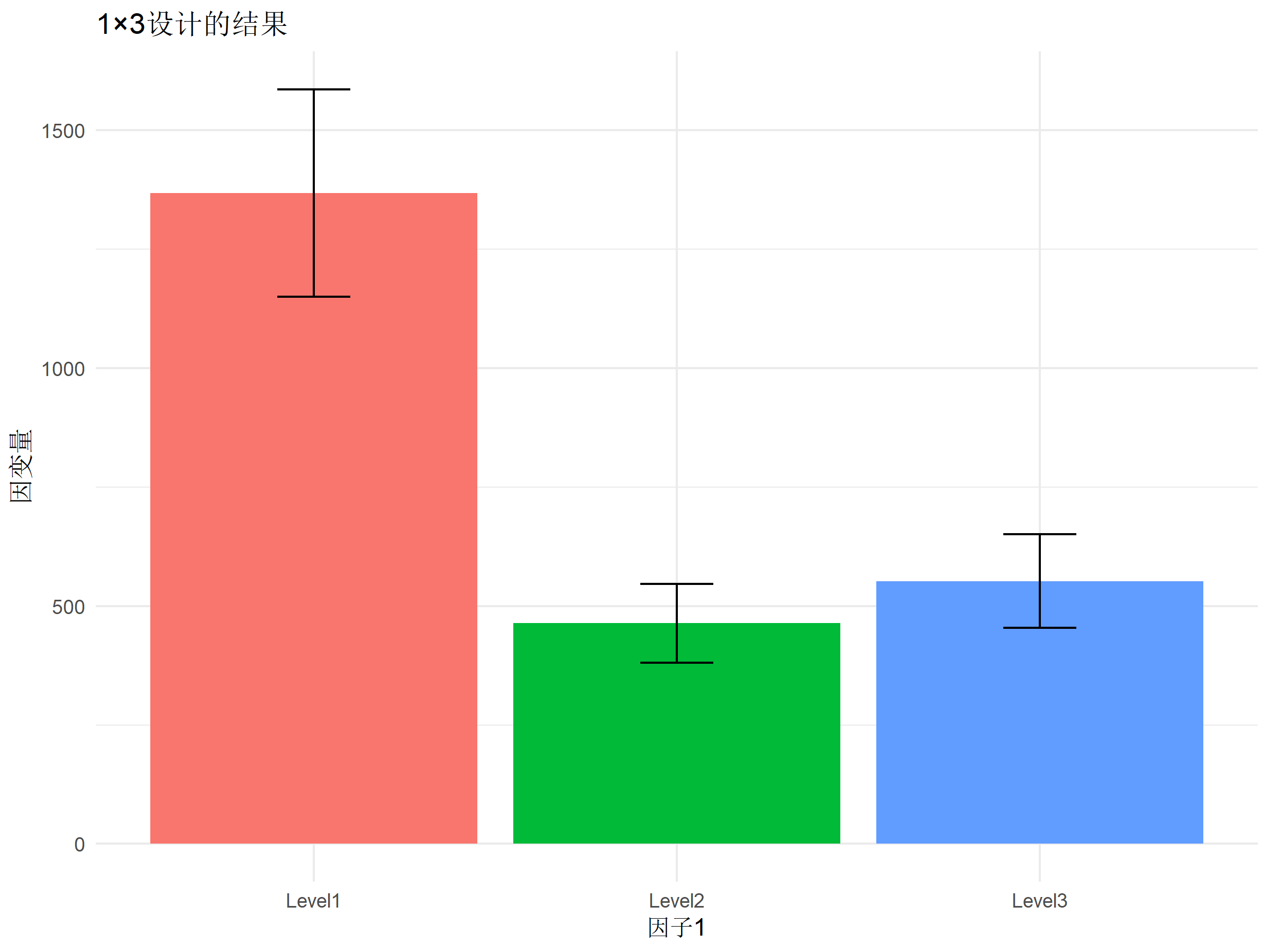

Supplement: Supplementary file 1 [file jemr-18-00033-s001.zip › local/Total_duration_of_fixations2/result_plot.png]

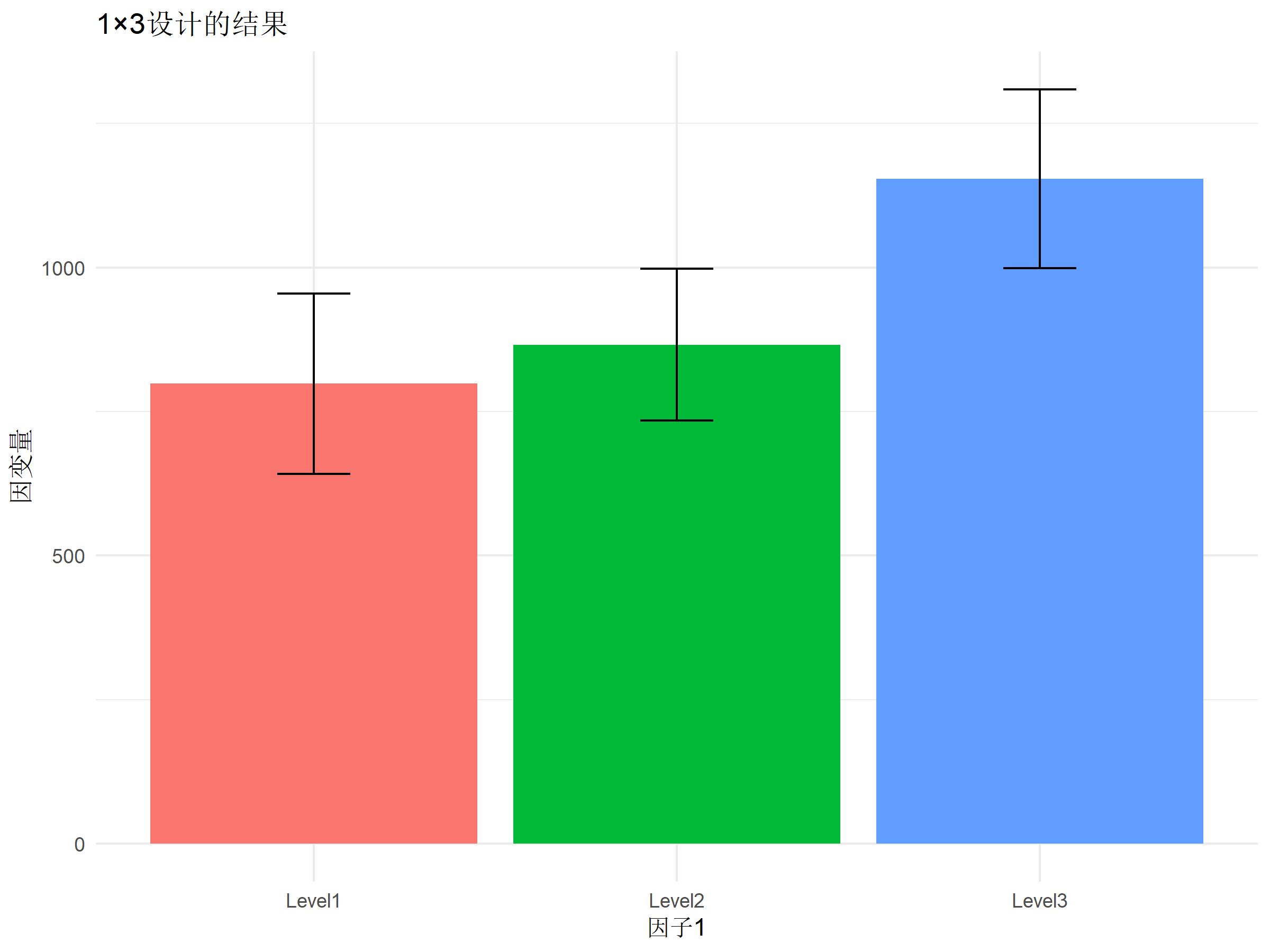

Supplement: Supplementary file 1 [file jemr-18-00033-s001.zip › local/Total_duration_of_fixations3/result_plot.png]

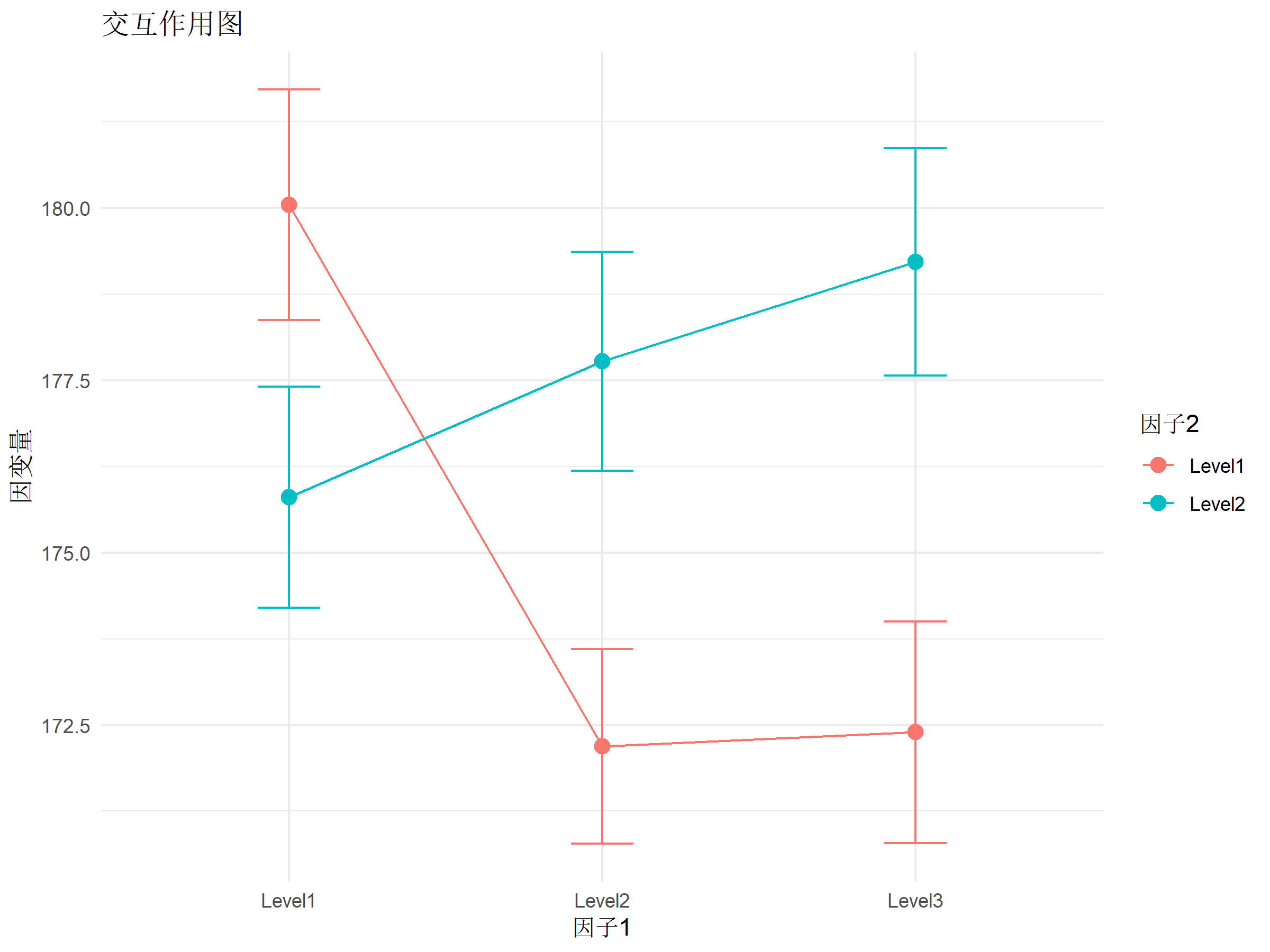

Supplement: Supplementary file 1 [file jemr-18-00033-s001.zip › global/1_Average_duration_of_whole_fixations/interaction_plot.png]

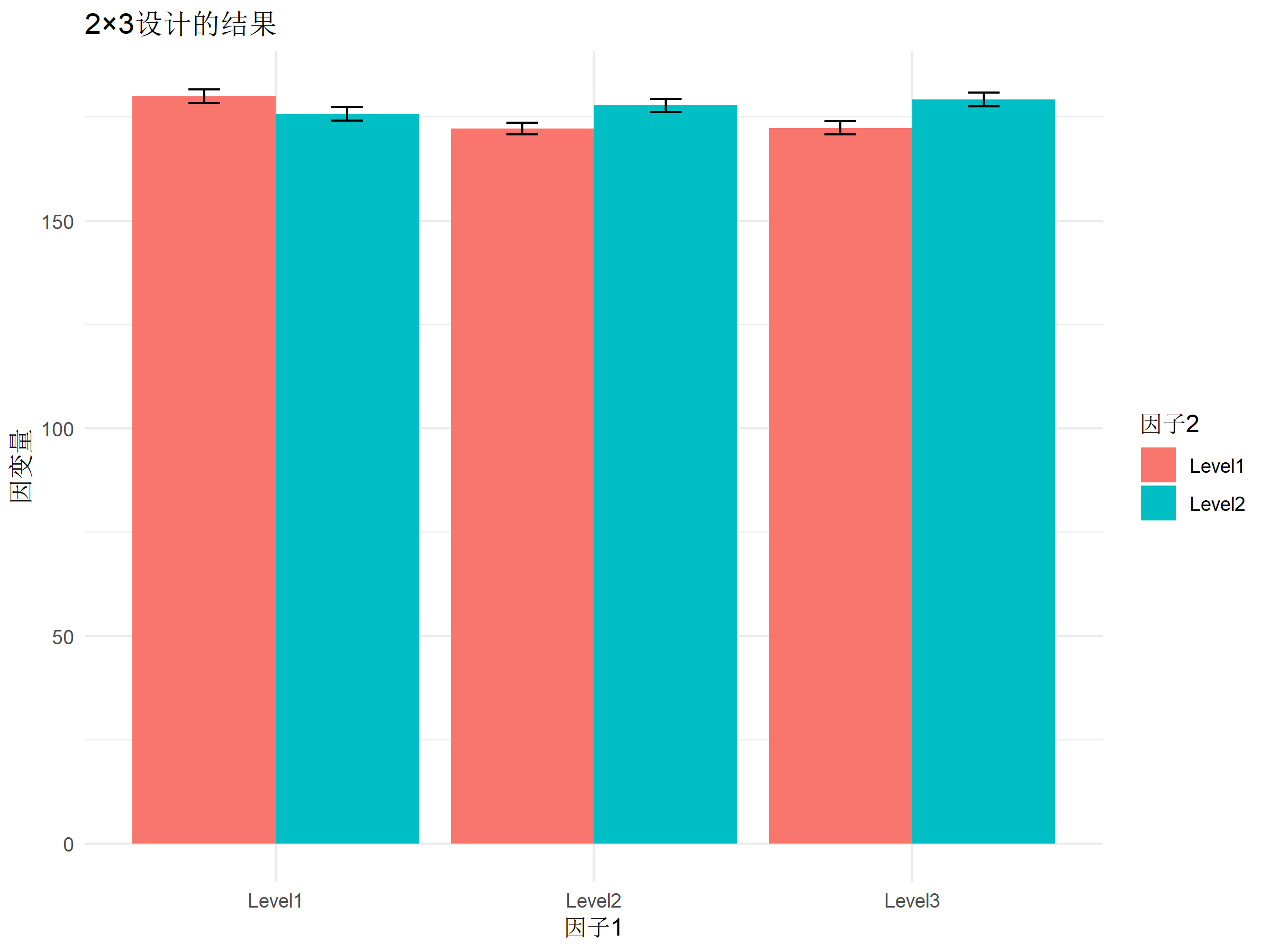

Supplement: Supplementary file 1 [file jemr-18-00033-s001.zip › global/1_Average_duration_of_whole_fixations/result_plot.png]

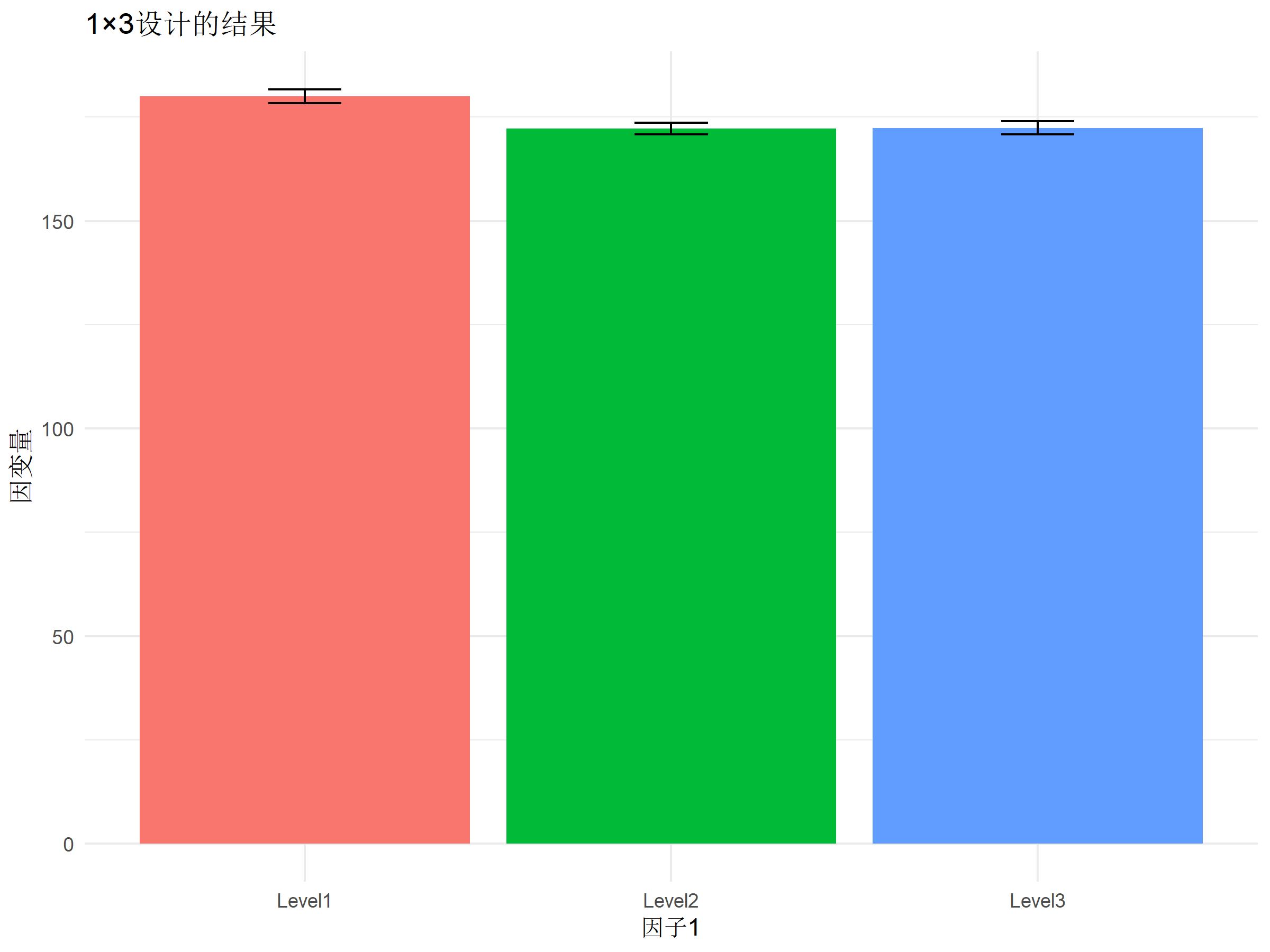

Supplement: Supplementary file 1 [file jemr-18-00033-s001.zip › global/1_Average_duration_of_whole_fixations2/result_plot.png]

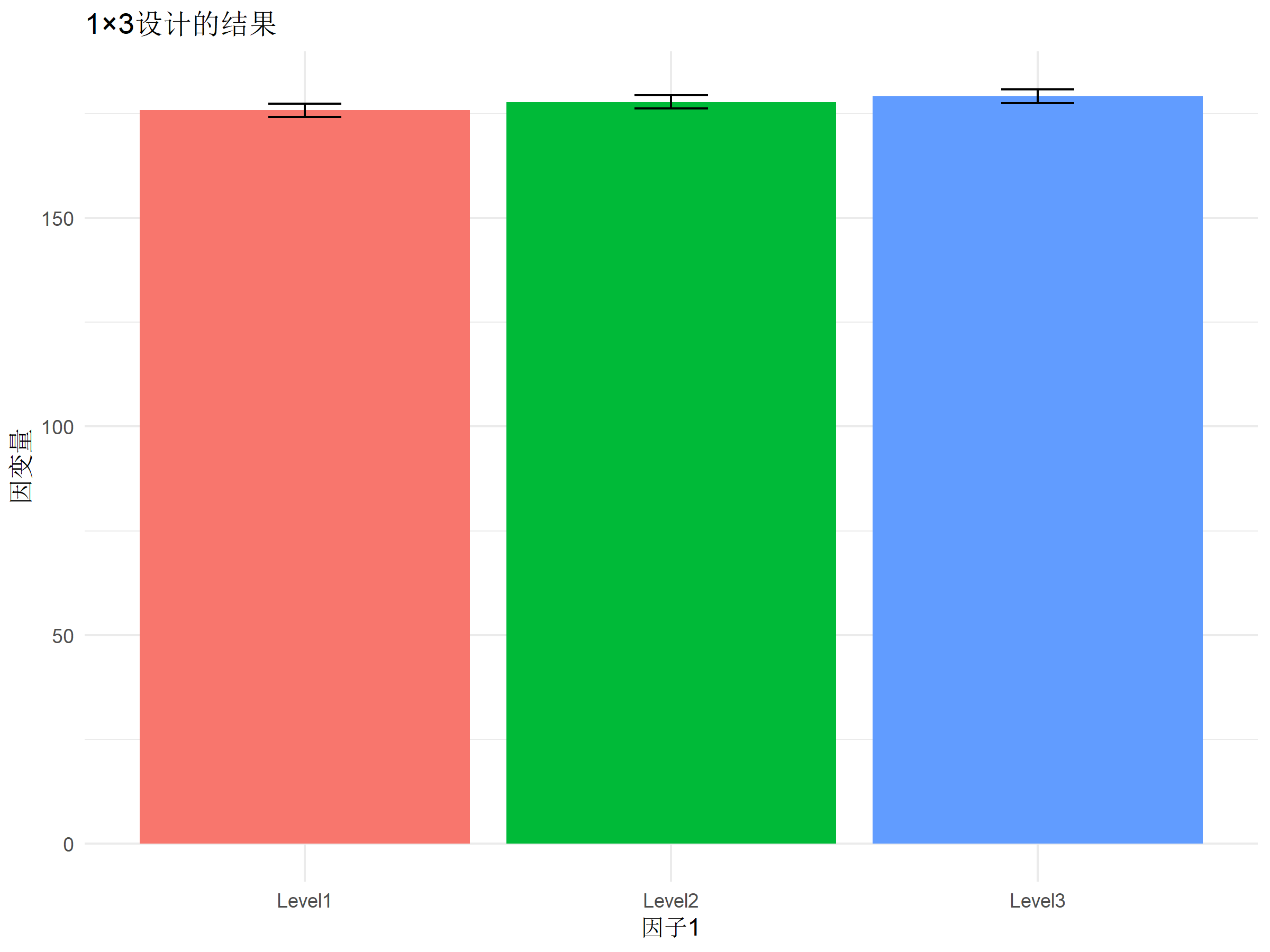

Supplement: Supplementary file 1 [file jemr-18-00033-s001.zip › global/1_Average_duration_of_whole_fixations3/result_plot.png]

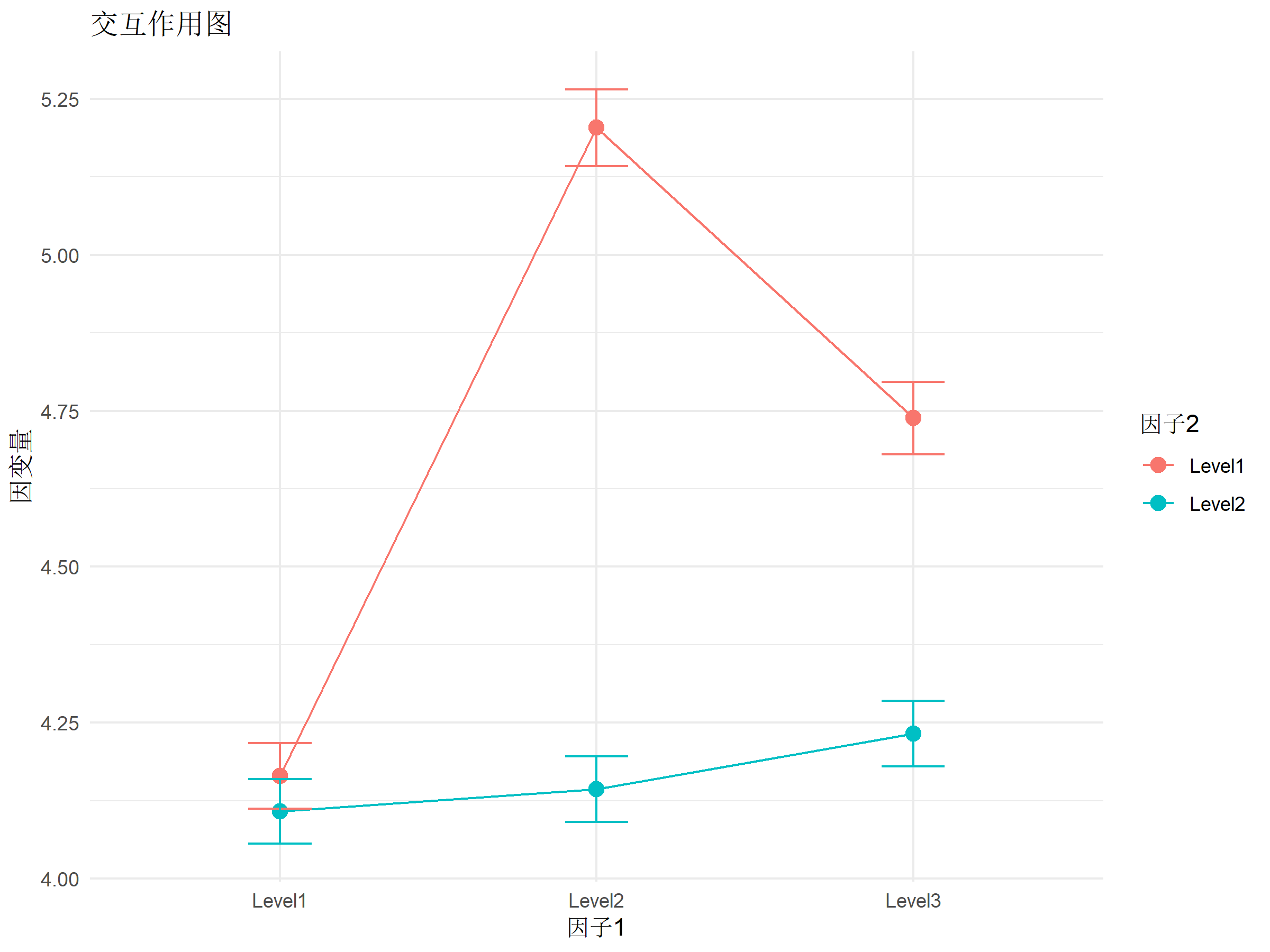

Supplement: Supplementary file 1 [file jemr-18-00033-s001.zip › global/1_Average_saccade_length/interaction_plot.png]

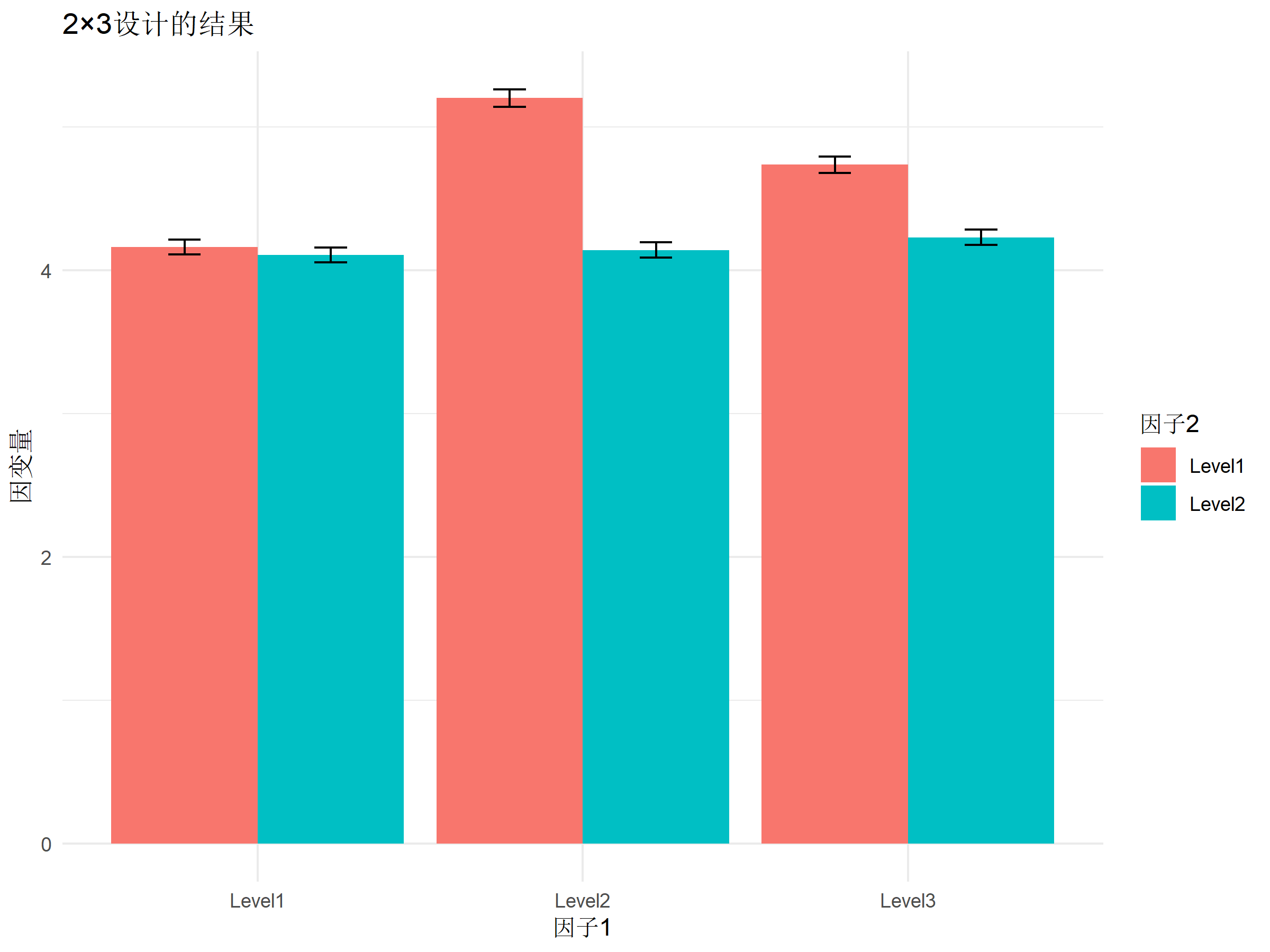

Supplement: Supplementary file 1 [file jemr-18-00033-s001.zip › global/1_Average_saccade_length/result_plot.png]

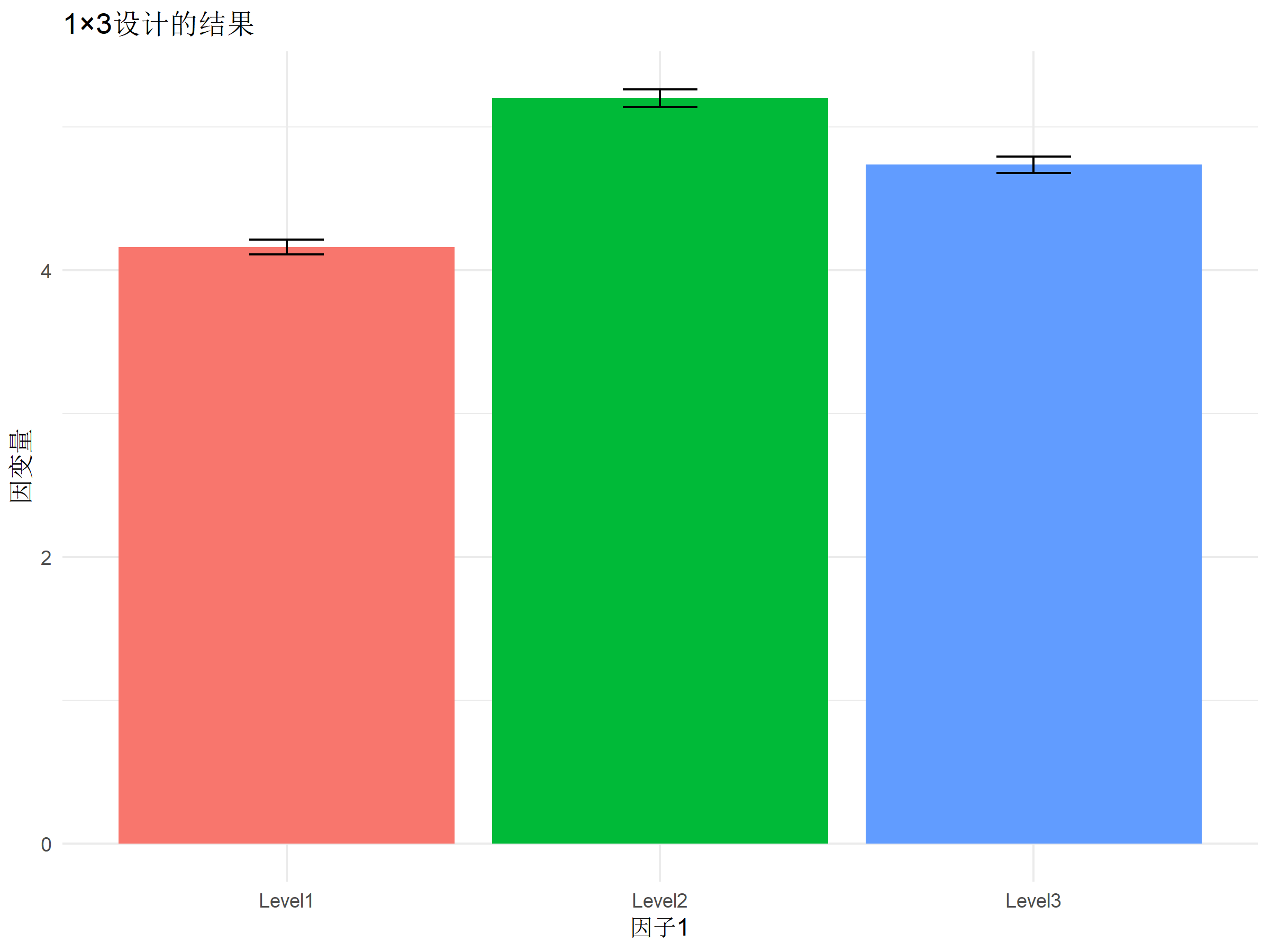

Supplement: Supplementary file 1 [file jemr-18-00033-s001.zip › global/1_Average_saccade_length2/result_plot.png]

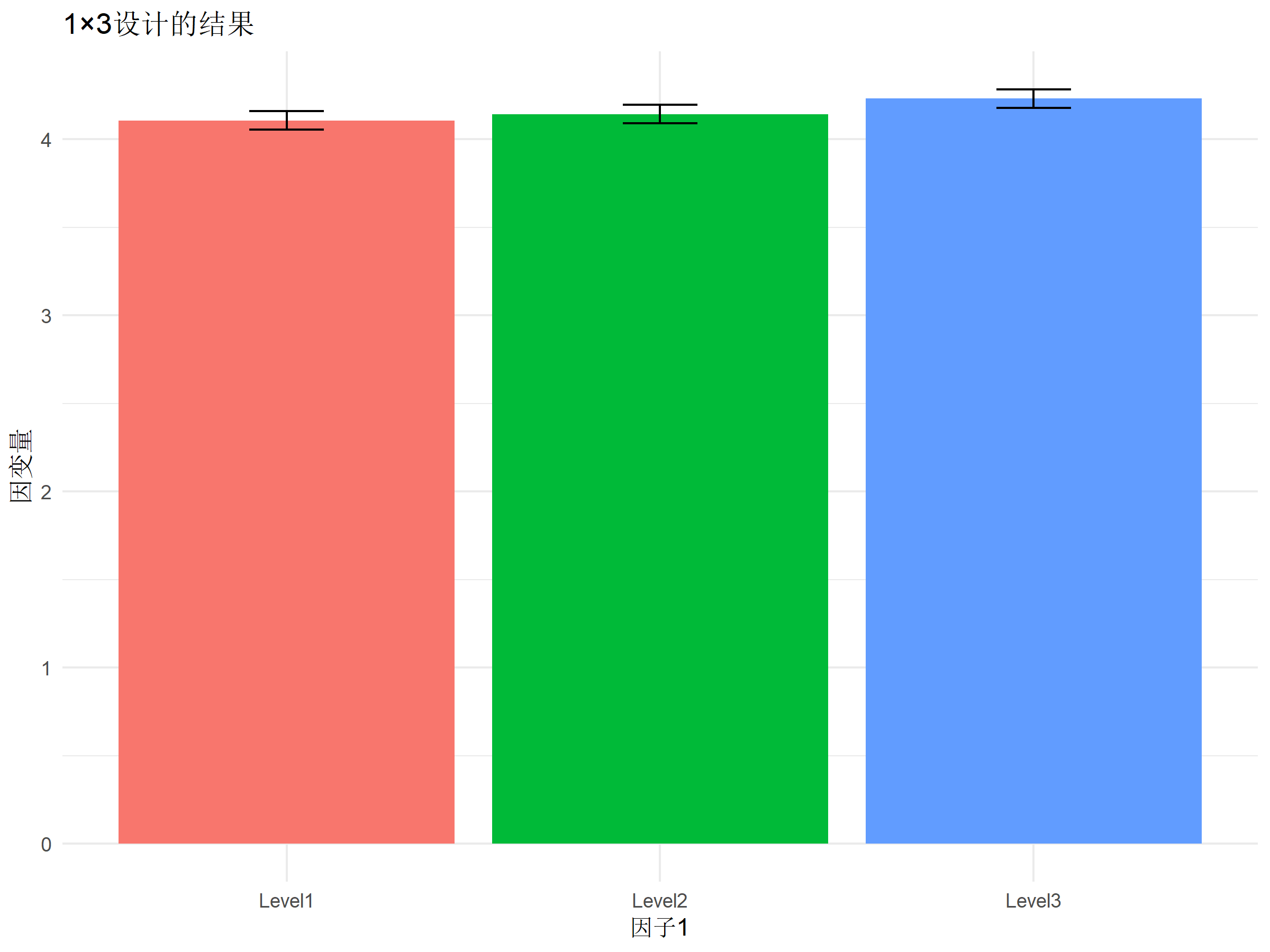

Supplement: Supplementary file 1 [file jemr-18-00033-s001.zip › global/1_Average_saccade_length3/result_plot.png]

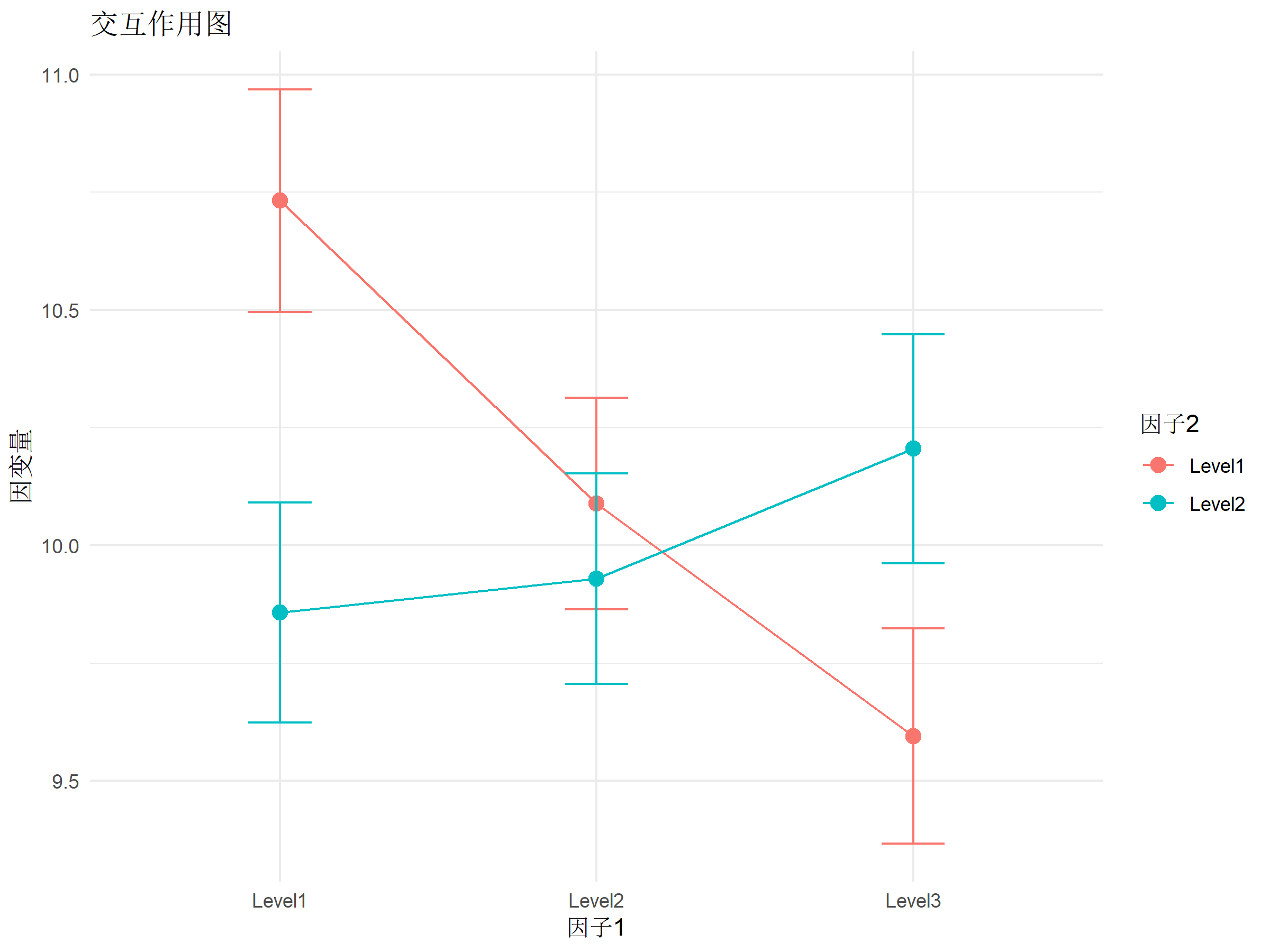

Supplement: Supplementary file 1 [file jemr-18-00033-s001.zip › global/1_number_of_whole_fixations/interaction_plot.png]

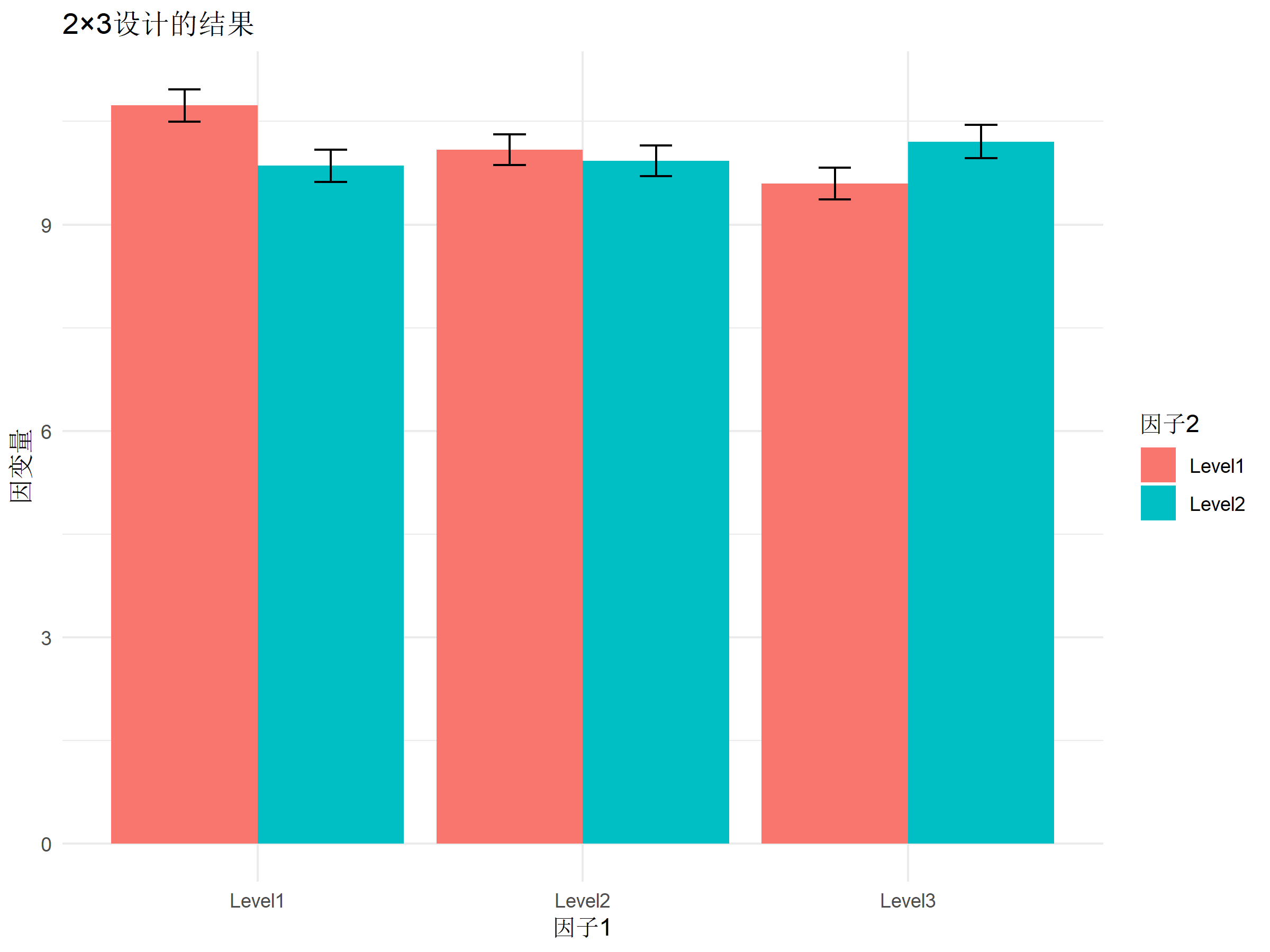

Supplement: Supplementary file 1 [file jemr-18-00033-s001.zip › global/1_number_of_whole_fixations/result_plot.png]

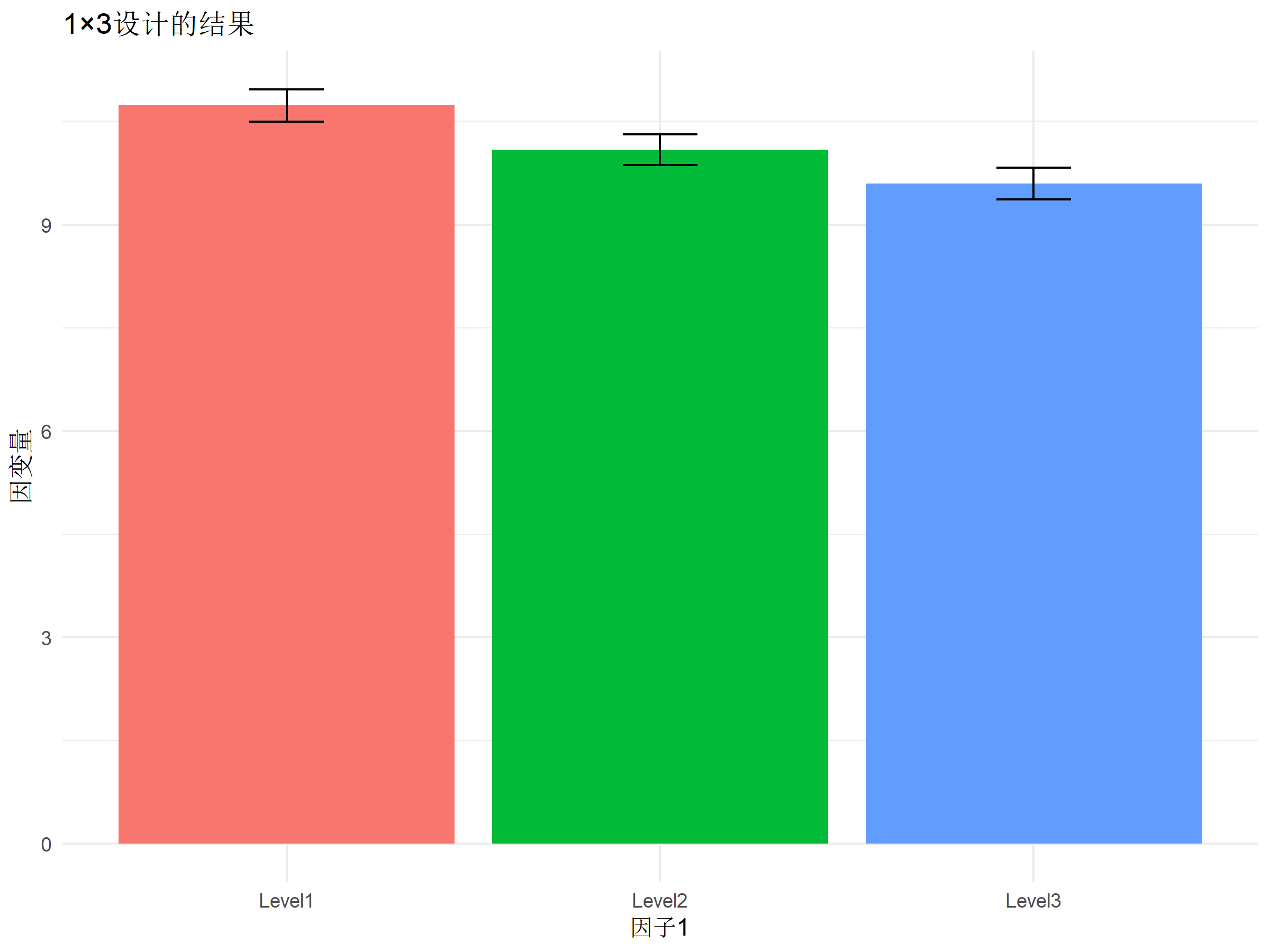

Supplement: Supplementary file 1 [file jemr-18-00033-s001.zip › global/1_number_of_whole_fixations2/result_plot.png]

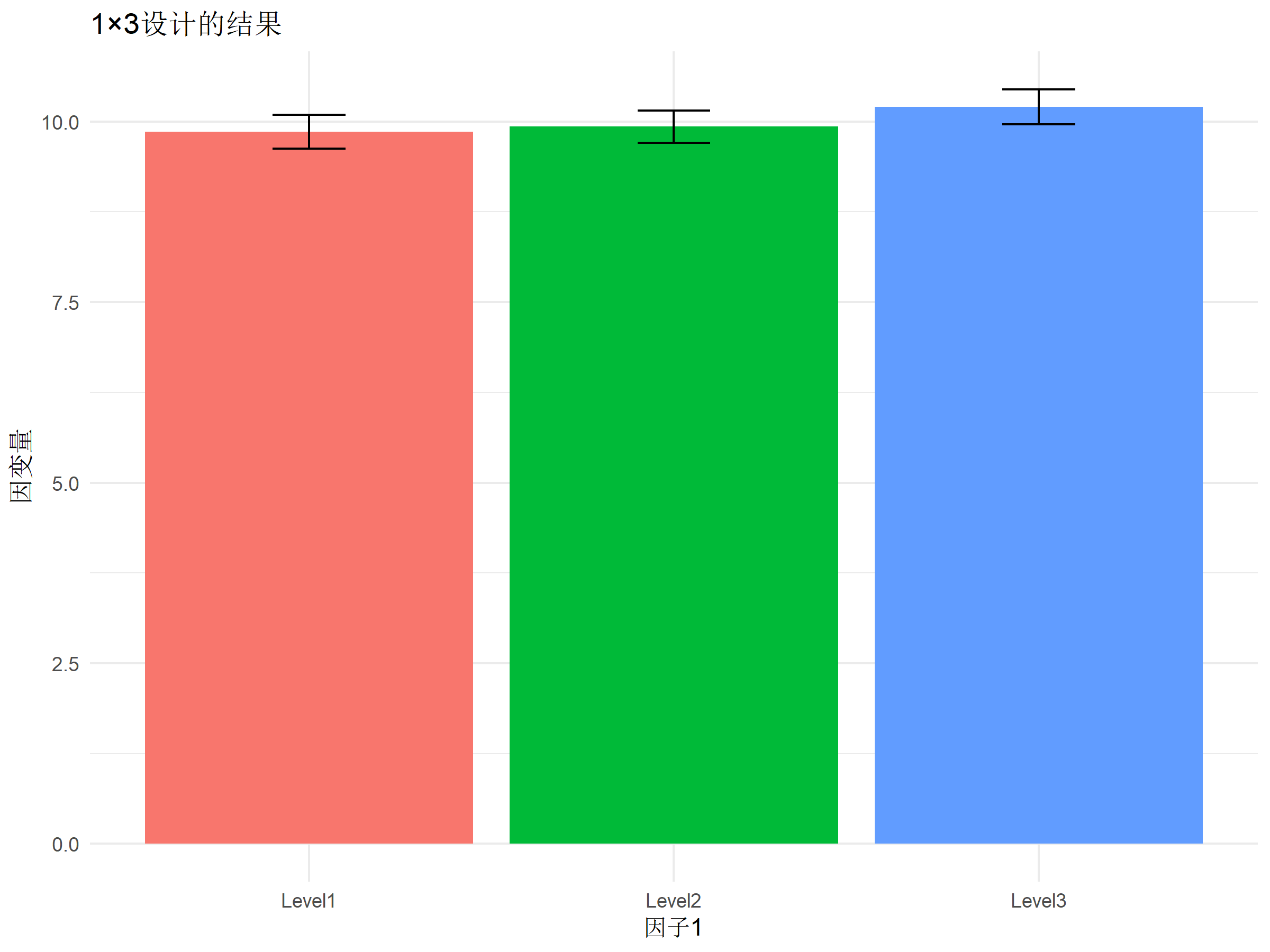

Supplement: Supplementary file 1 [file jemr-18-00033-s001.zip › global/1_number_of_whole_fixations3/result_plot.png]

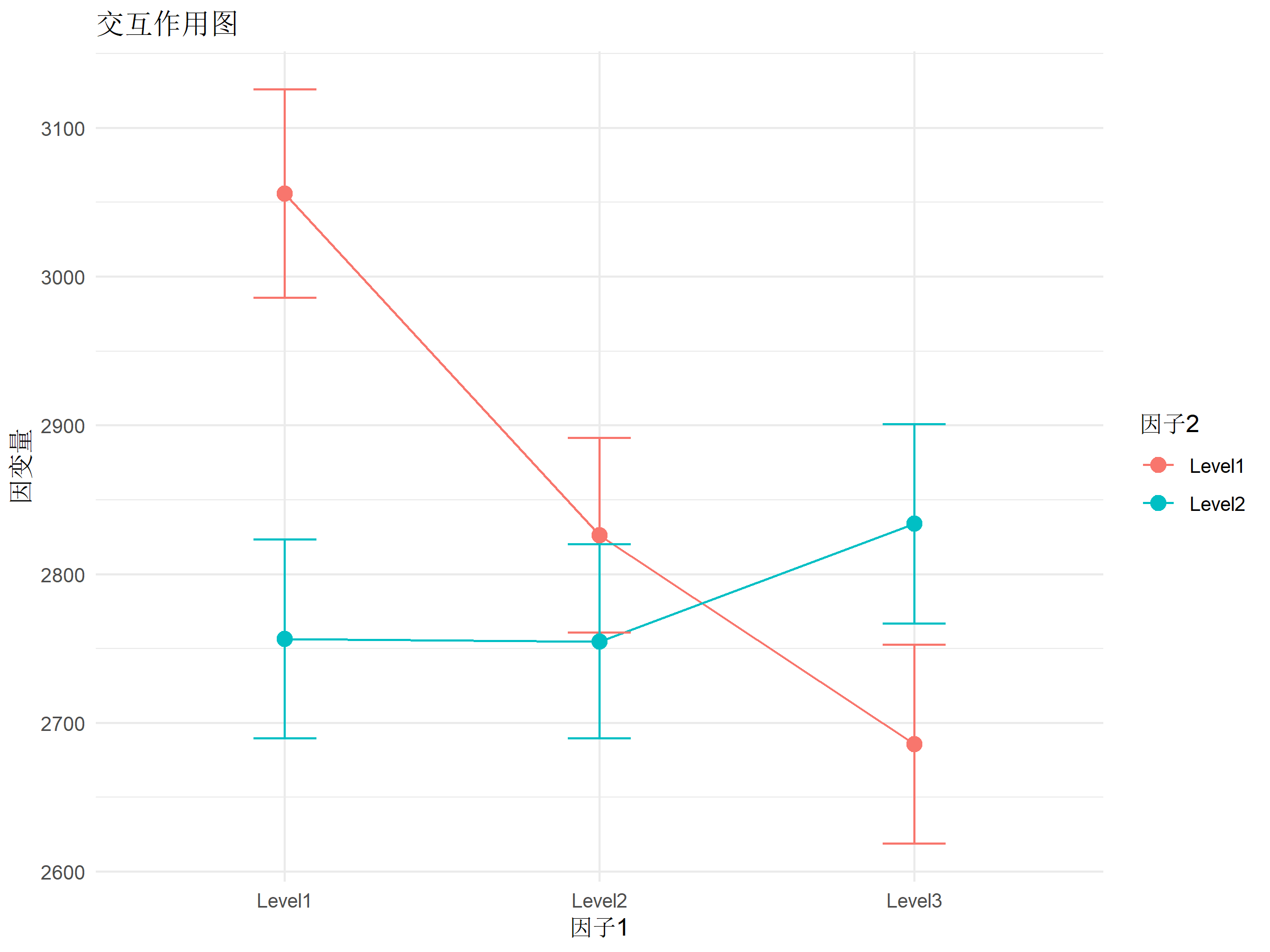

Supplement: Supplementary file 1 [file jemr-18-00033-s001.zip › global/1_reading_time/interaction_plot.png]

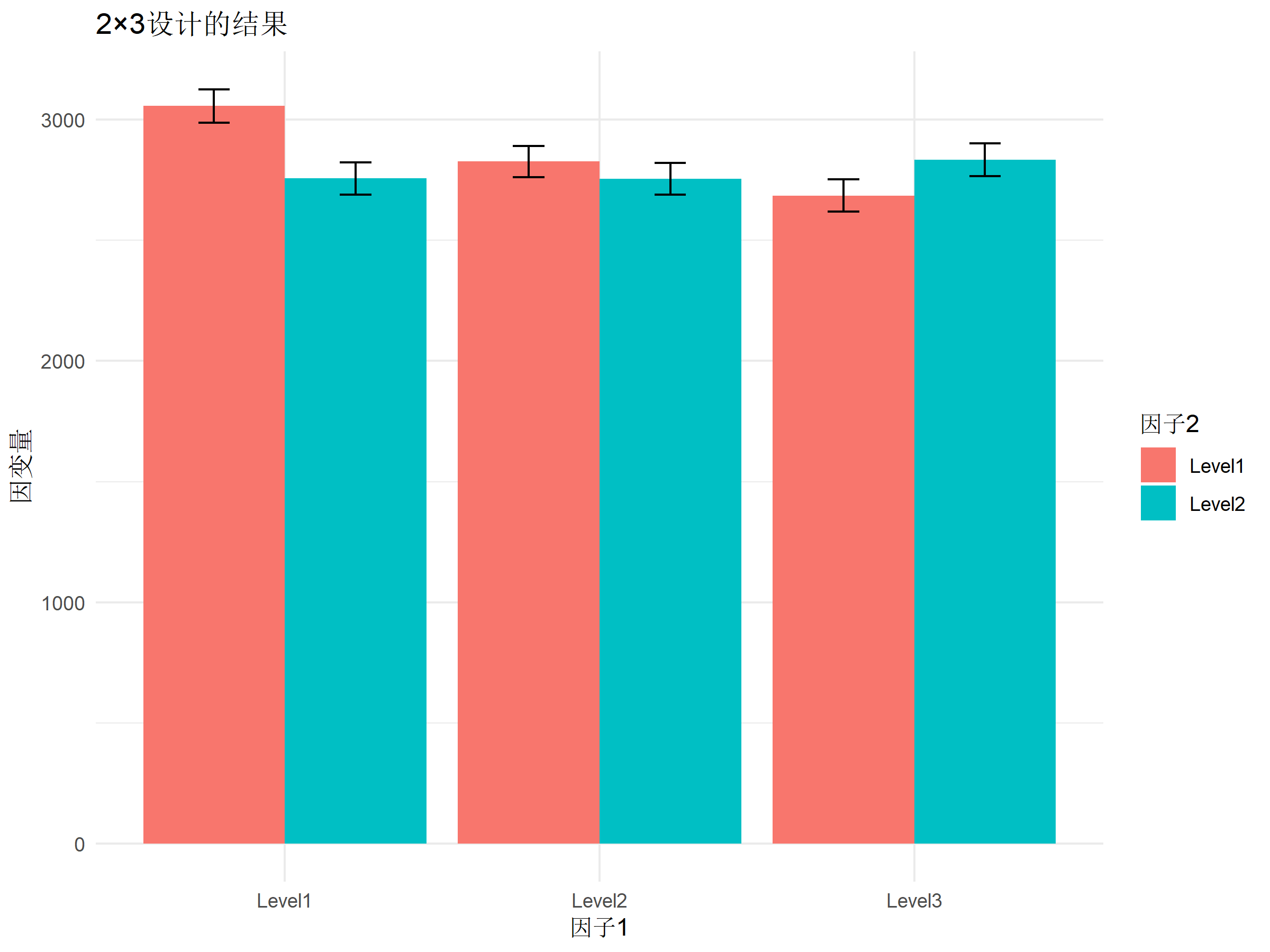

Supplement: Supplementary file 1 [file jemr-18-00033-s001.zip › global/1_reading_time/result_plot.png]

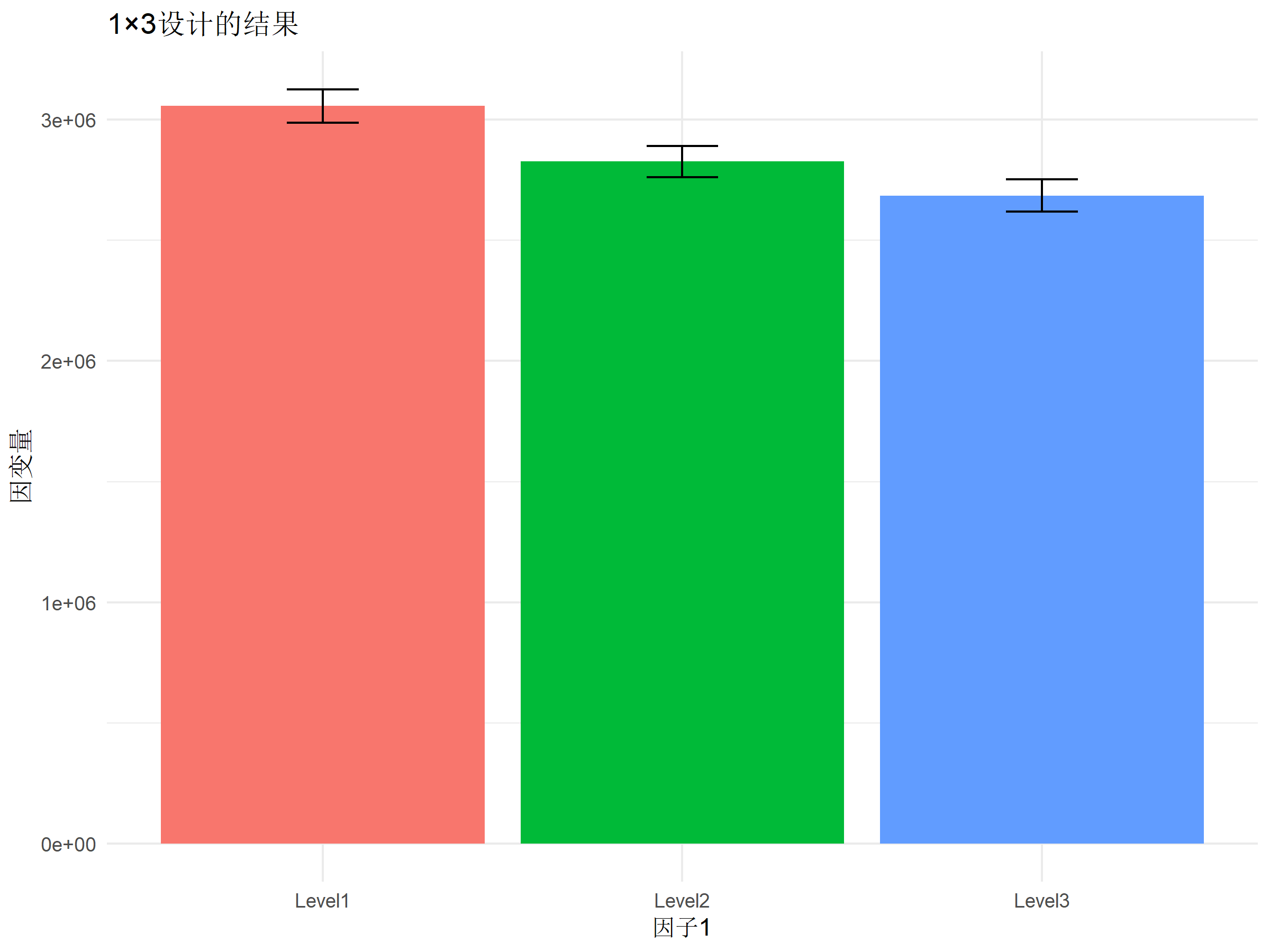

Supplement: Supplementary file 1 [file jemr-18-00033-s001.zip › global/1_reading_time2/result_plot.png]

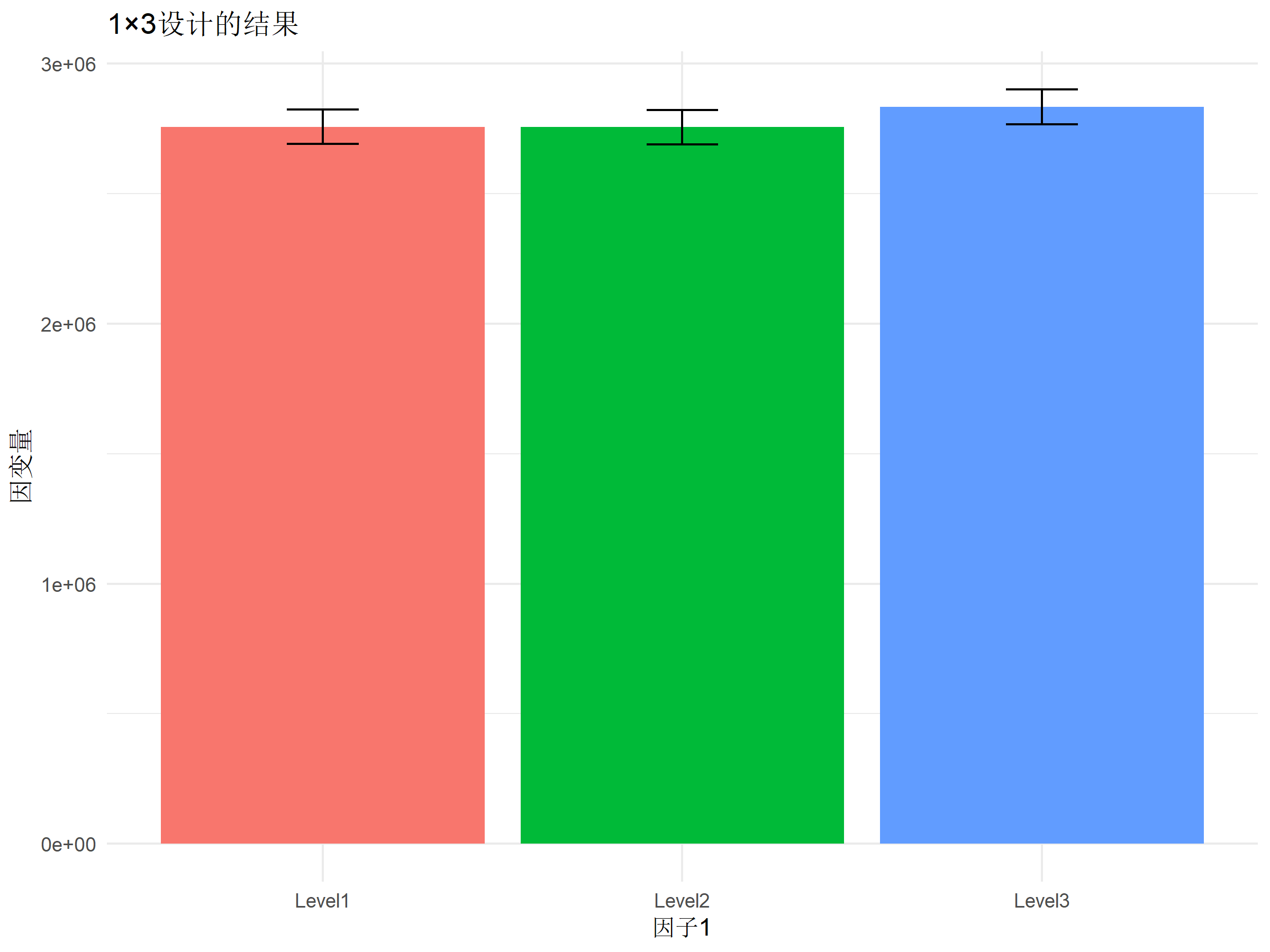

Supplement: Supplementary file 1 [file jemr-18-00033-s001.zip › global/1_reading_time3/result_plot.png]

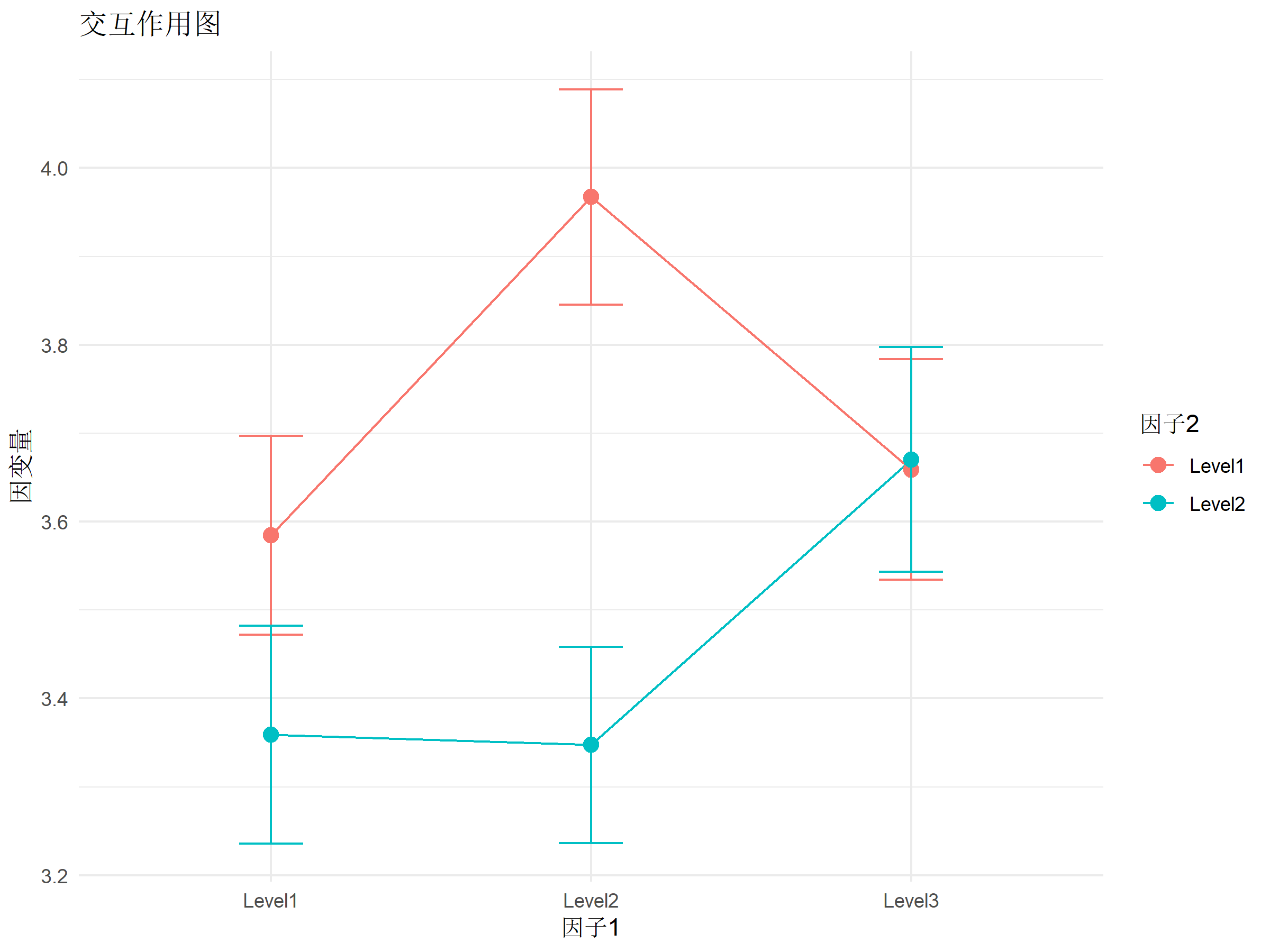

Supplement: Supplementary file 1 [file jemr-18-00033-s001.zip › global/left/interaction_plot.png]

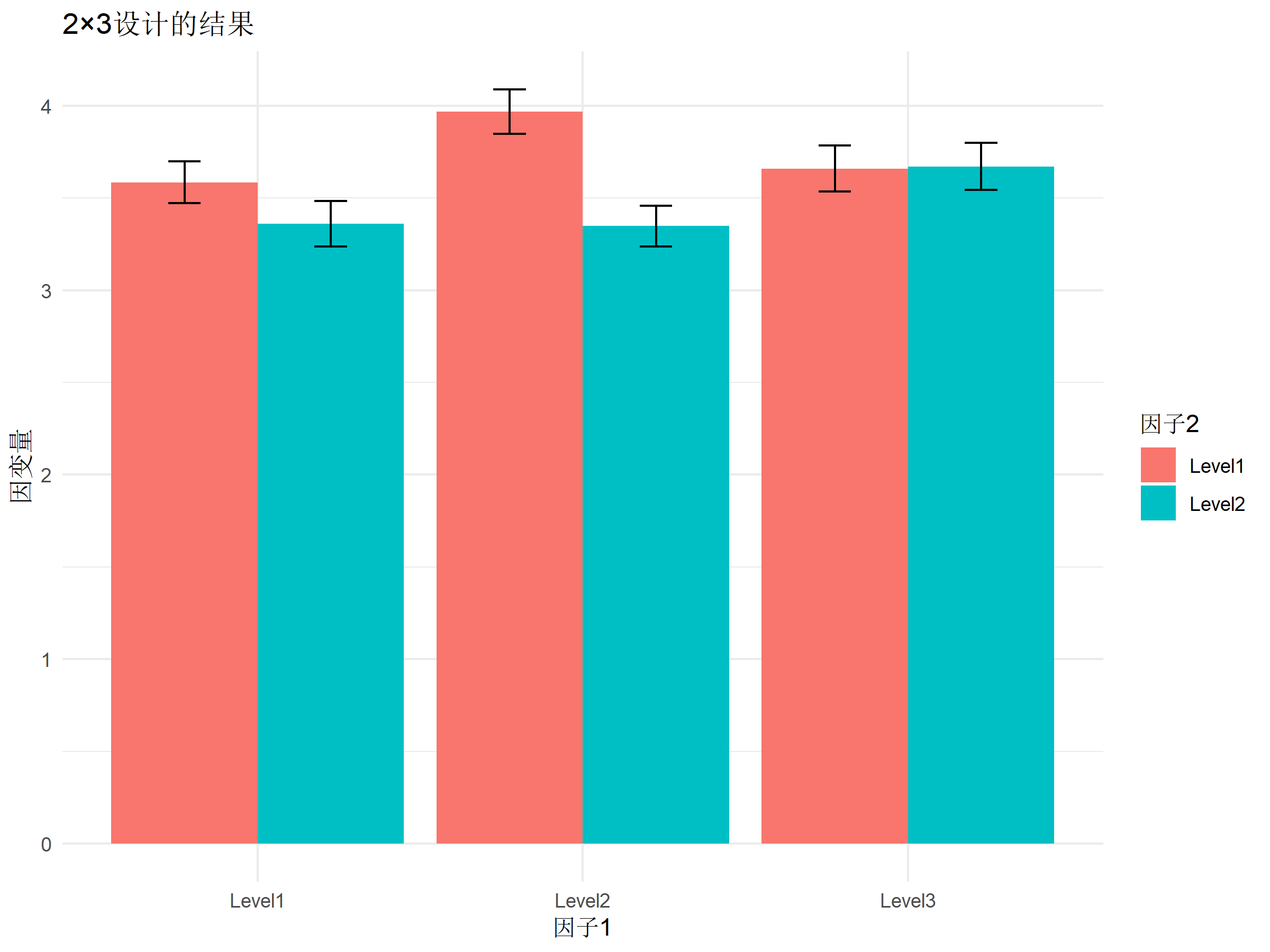

Supplement: Supplementary file 1 [file jemr-18-00033-s001.zip › global/left/result_plot.png]

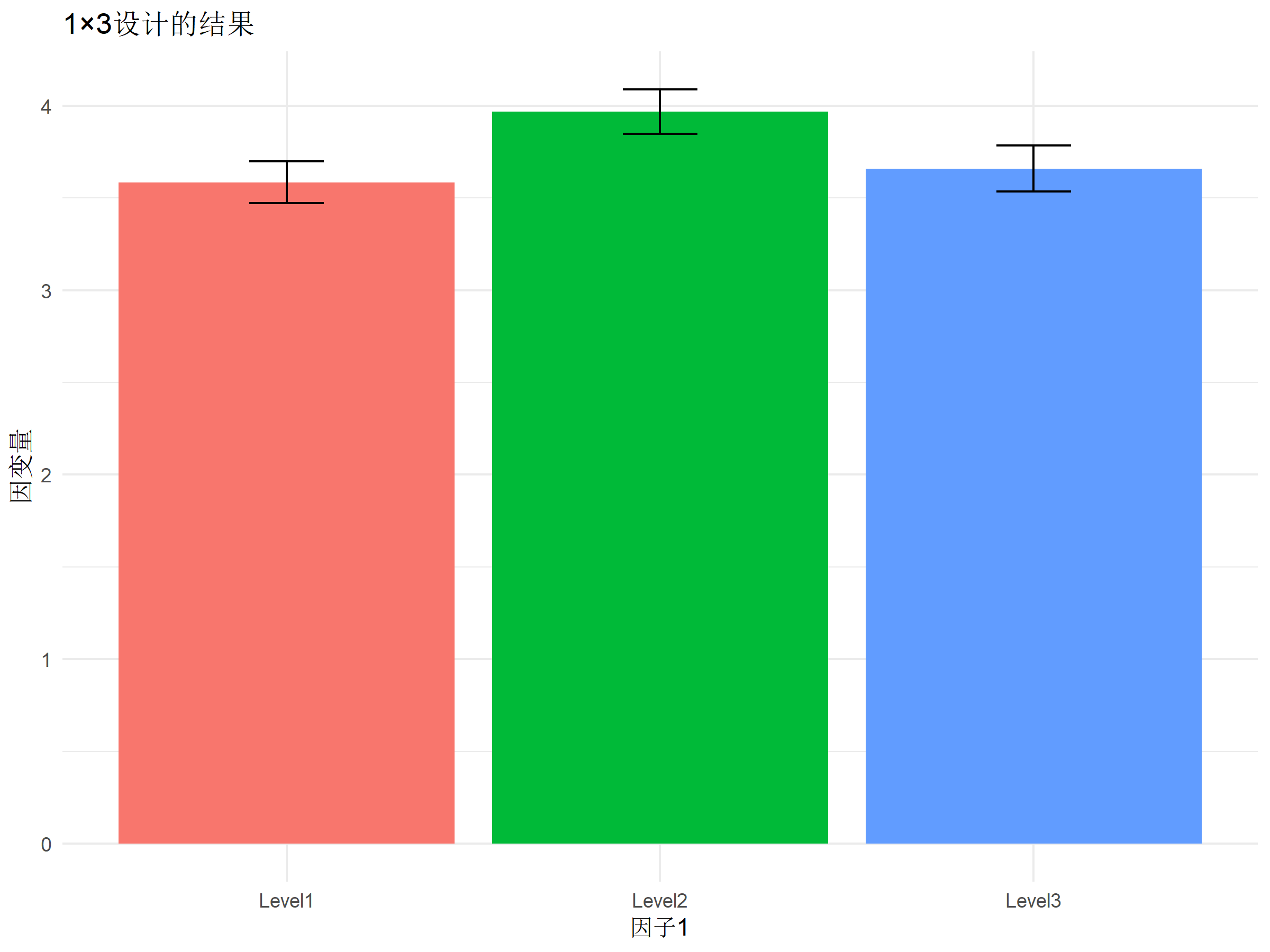

Supplement: Supplementary file 1 [file jemr-18-00033-s001.zip › global/left2/result_plot.png]

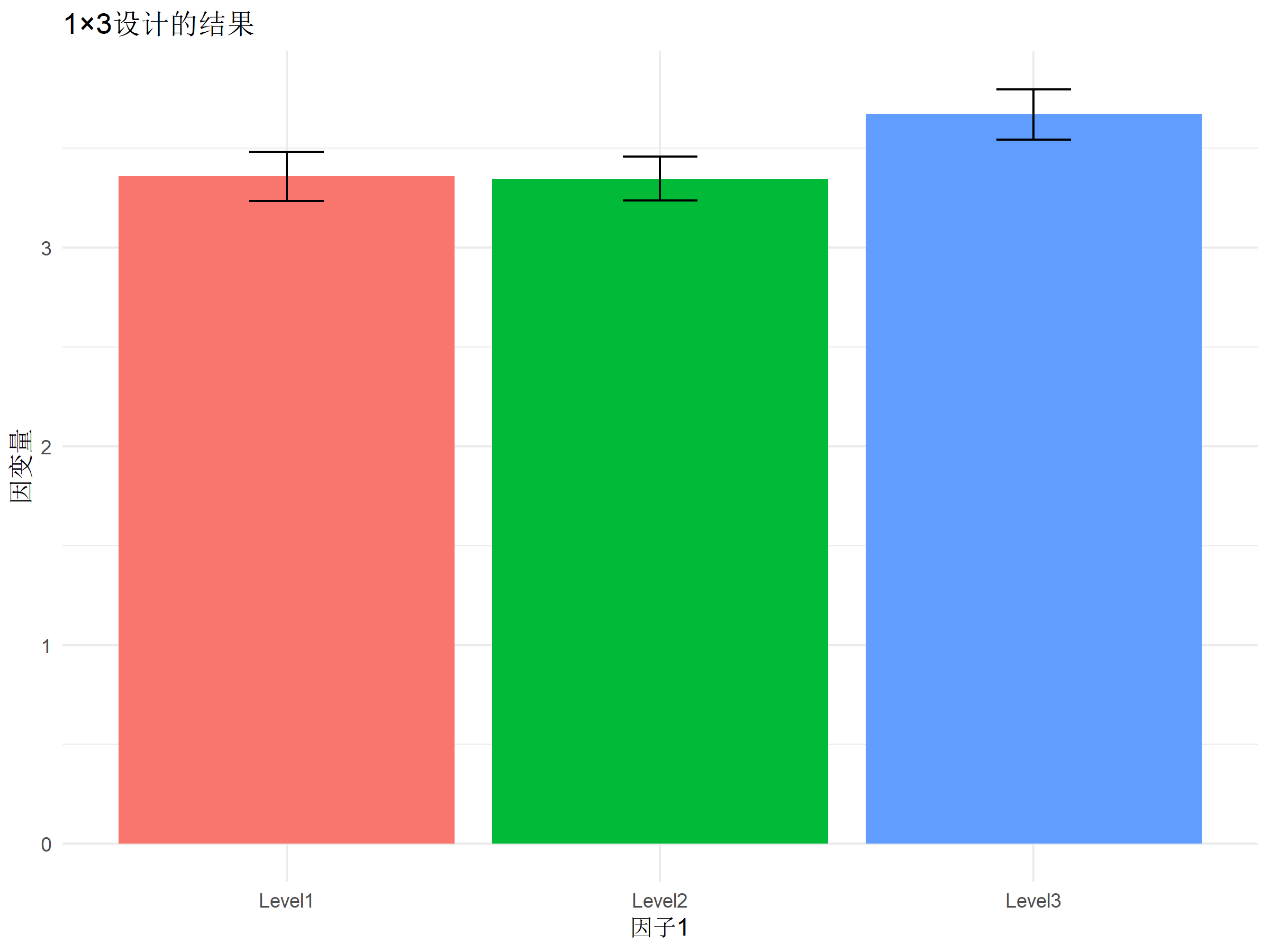

Supplement: Supplementary file 1 [file jemr-18-00033-s001.zip › global/left3/result_plot.png]

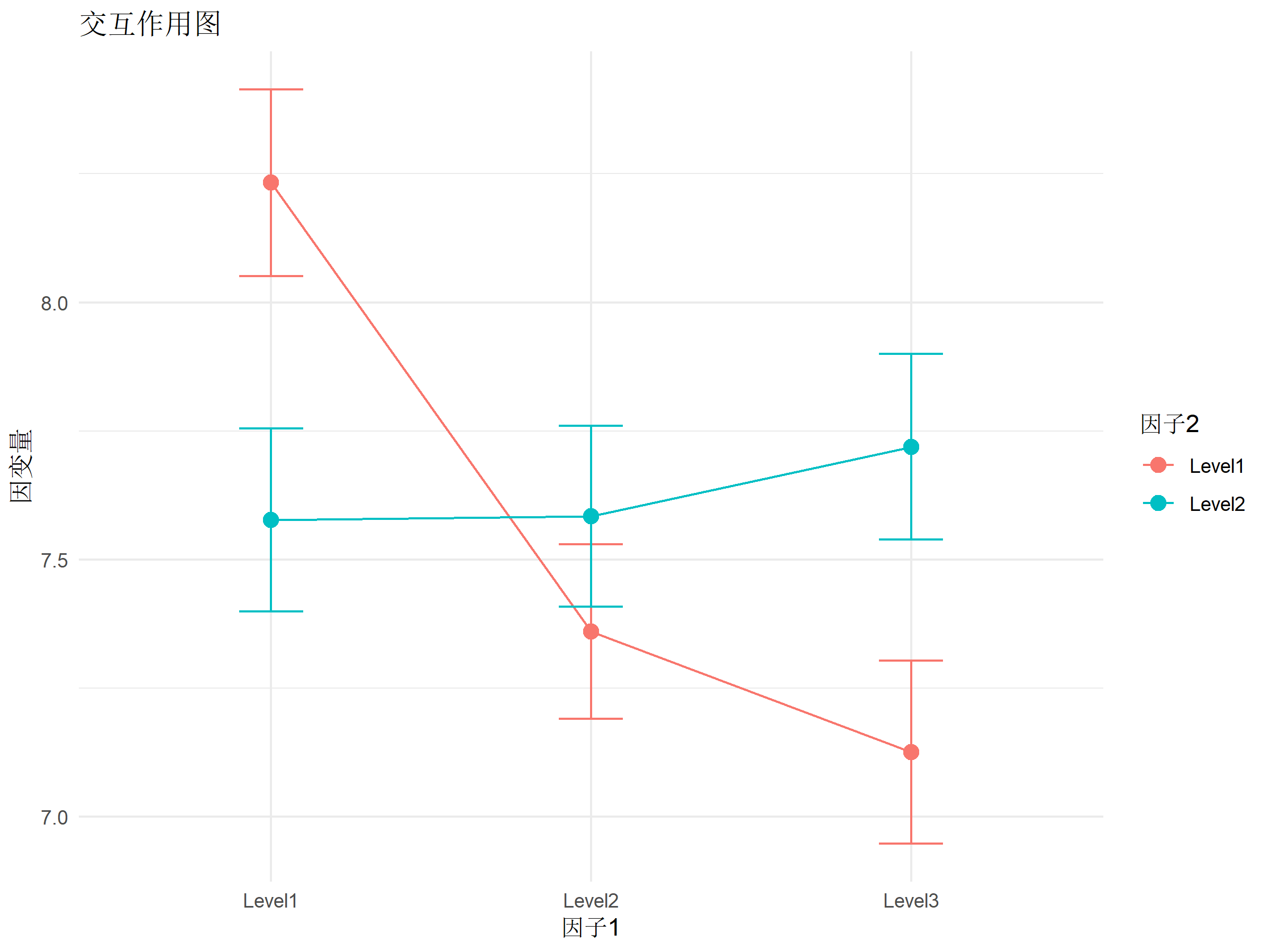

Supplement: Supplementary file 1 [file jemr-18-00033-s001.zip › global/right/interaction_plot.png]

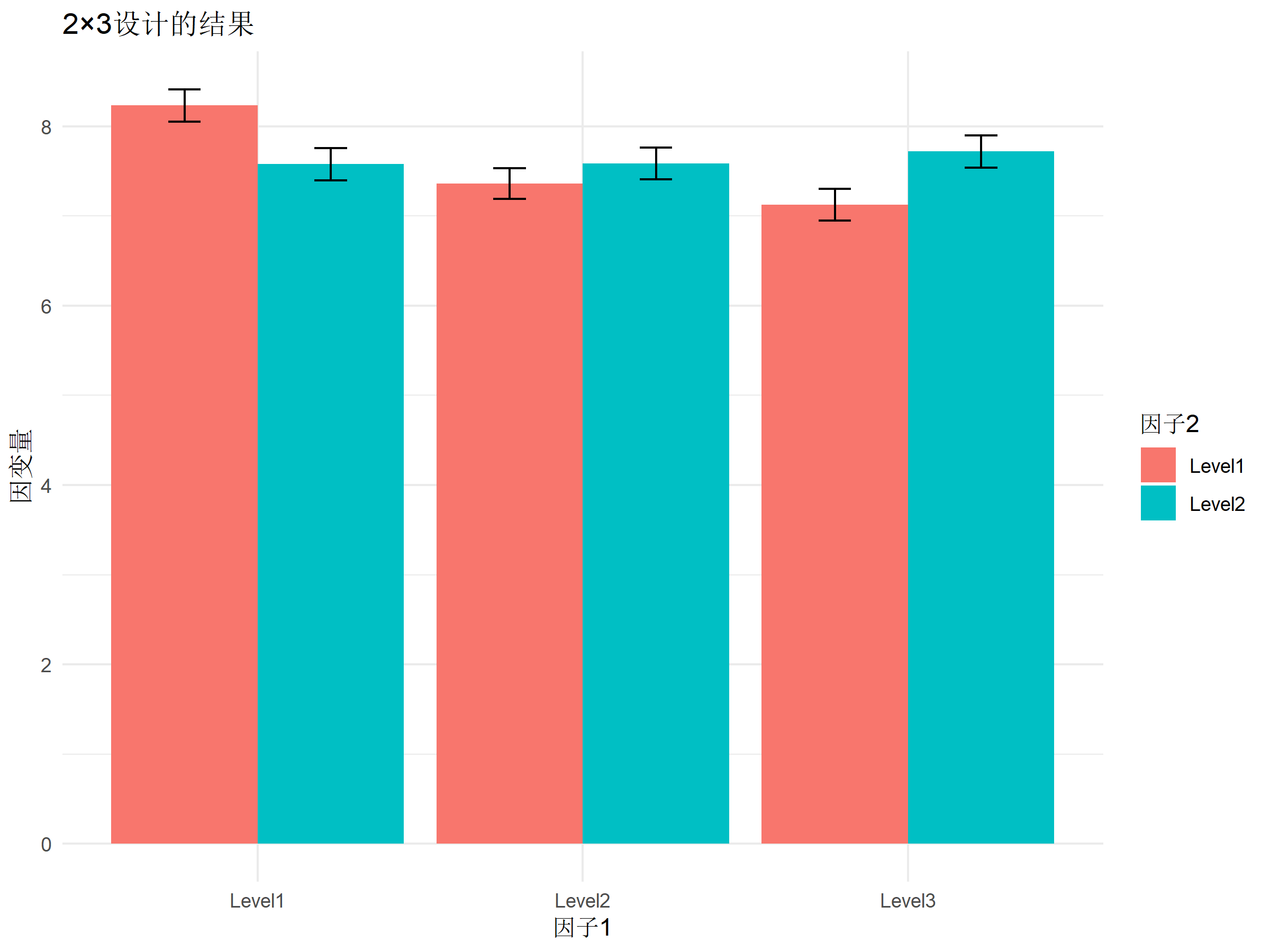

Supplement: Supplementary file 1 [file jemr-18-00033-s001.zip › global/right/result_plot.png]

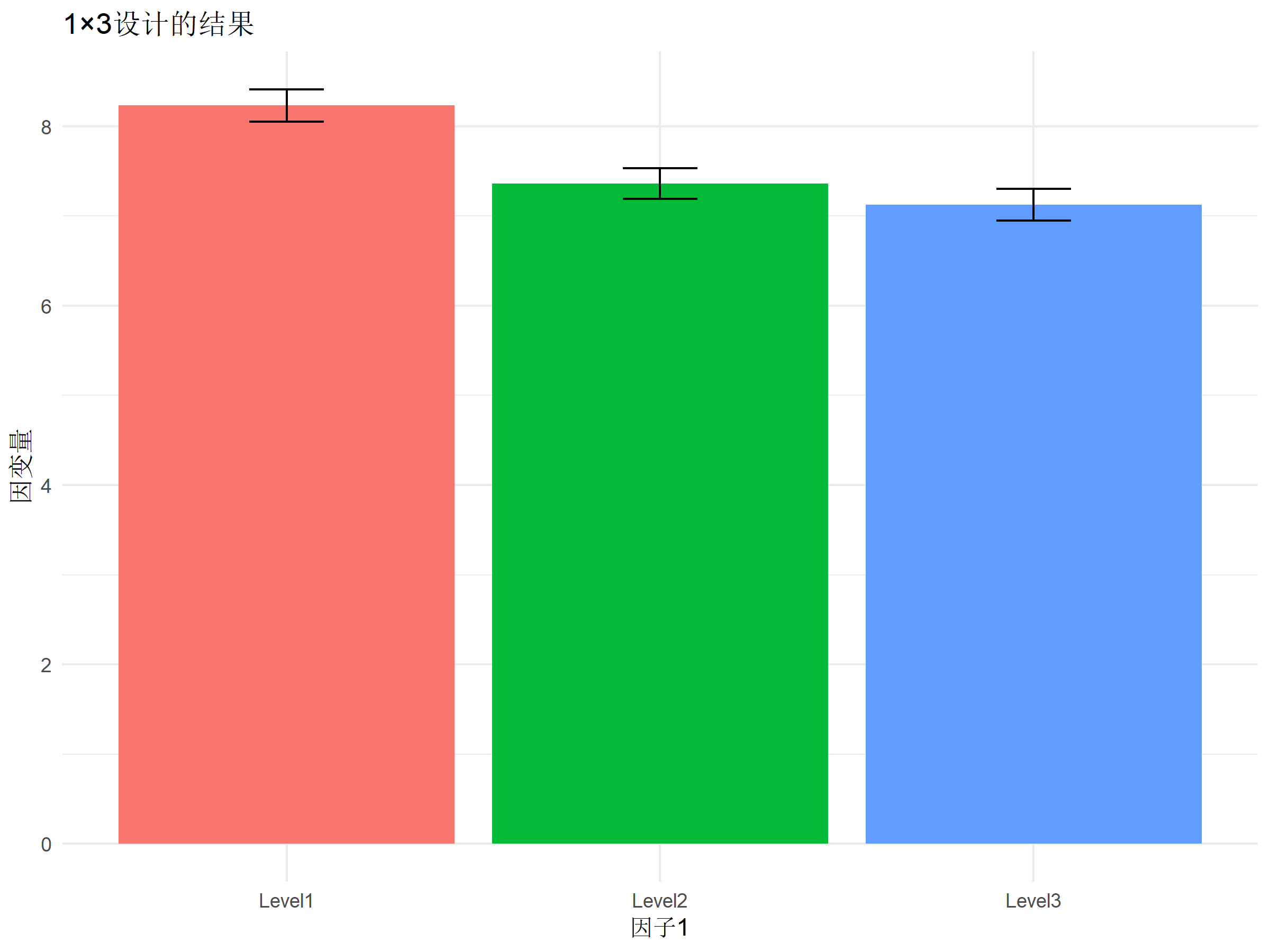

Supplement: Supplementary file 1 [file jemr-18-00033-s001.zip › global/right2/result_plot.png]

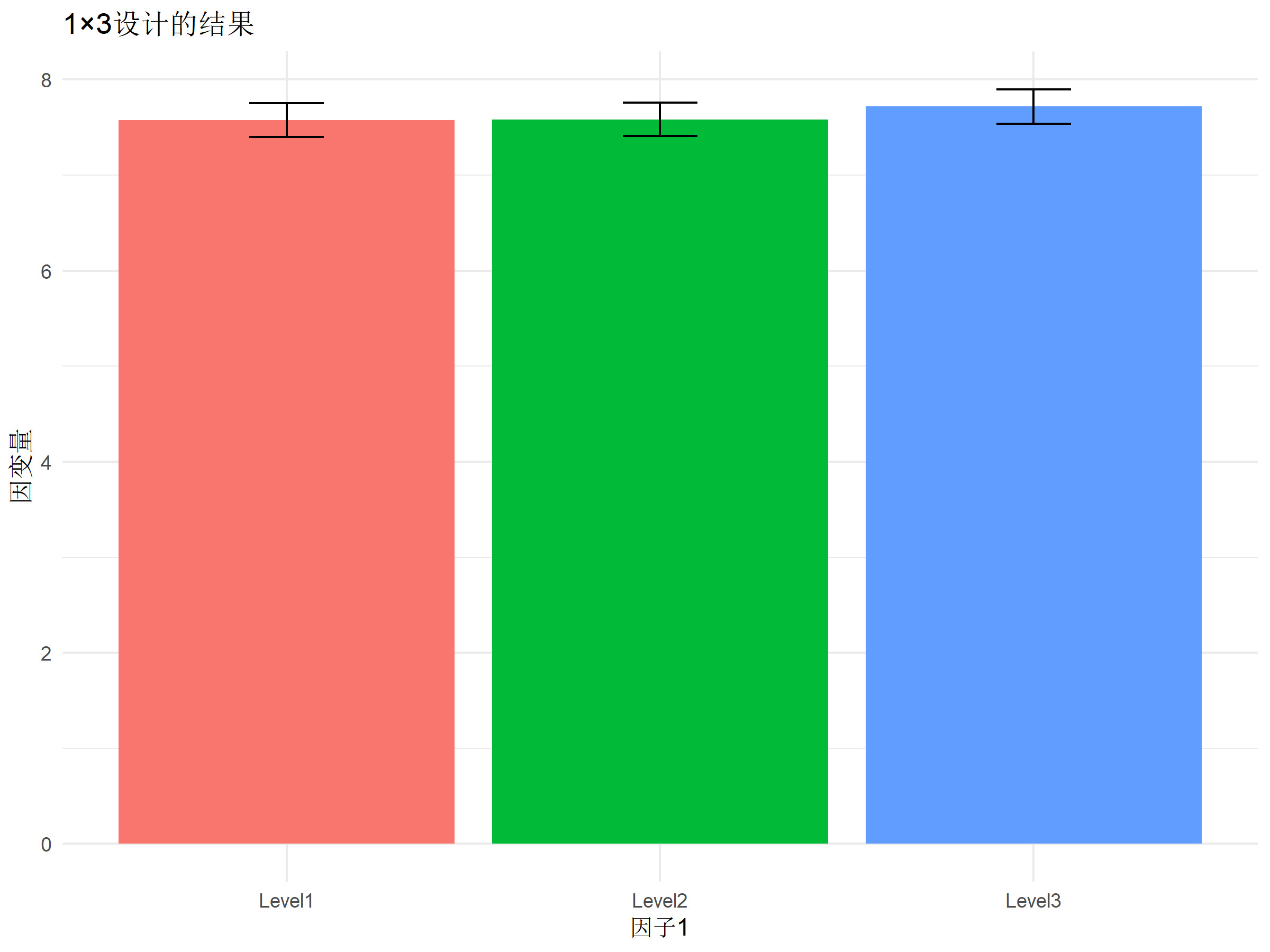

Supplement: Supplementary file 1 [file jemr-18-00033-s001.zip › global/right3/result_plot.png]
